# Supplementary material for: From Anti-PD-1/PD-L1 to CTLA-4 and to MUC1—Is the Better Response to Treatment in Smokers of Cancer Patients Drug Specific?
Source: J Pers Med. 2021 Sep 13;11(9):914. doi: 10.3390/jpm11090914 (PMC8471889; doi:10.3390/jpm11090914)
Supplement: Supplementary file 1 [file jpm-11-00914-s001.zip › jpm-1311393-supplementary.pdf]

Supplementary Table S1. HR values of smokers and non-smoker treated with different drugs for meta-analysis

First author	Ref. No.	ANALYTIC METRIX	first/second Treatment	Drugs	Year	Current/#Patients	Former/#Patients	Never/#Patients	Overall/#Patients	Disease
**PD-1/PD-L1 (NSCLC)**										
**Reck**	(Reck, Rodriguez-Abreu, et al., 2016)	PFS	First line	Pembrolizumab	2016	0·68(0·36-1·31)/65	0·47(0·33-0·67)/216	0·90(0·11-7·59)/24	0·50 (0·37-0·68)/305	NSCLCs
**Mok**	(Mok et al., 2019)-1	OS/TPS≥50%	First line	Pembrolizumab	2019	0·71(0·43-1·16)/116	0·60(0·46-0·80)/352	1·10(0·69-1·75)/131	0·69 (0·56-0·85) /599	NSCLC
**Mok**	(Mok et al., 2019)-2	OS/TPS≥20%	First line	Pembrolizumab	2019	0·80(0·53-1·21)/160	0·65(0·51-0·83)/473	1·26(0·84-1·89)/185	0·77 (0·64-0·92) /818	NSCLC
**Mok**	(Mok et al., 2019)-3	OS/TPS≥1%	First line	Pembrolizumab	2019	0·95(0·70-1·29)/271	0·71(0·59-0·86)/721	1·00(0·73-1·37)/282	0·81 (0·71-0·93) /1274	NSCLC
**Gandhi**	(Gandhi et al., 2018)-1	OS	Multitherapy	Pembrolizumab	2016	0·54(0·41-0·71)/543	-	0·23(0·10-0·54)/73	0·49(0·38-0·64)/616	NSCLC

**Gandhi**	(Gandhi et al., 2018)-2	PFS	Multitherapy	Pembrolizumab	2016	0·54(0·43-0·66)/543	-	0·43(0·23-0·81)/73	0·52(0·43-0·64)/616	NSCLC
**Borghaei**	(Borghaei et al., 2015)	OS	Monotherapy	Nivolumab	2015	0·70(0·56-0·86)/458	-	1·02(0·64-1·161)/118	0·75(0·62-0·91)//582	NSCLC
**Wu**	(Wu et al., 2019)	OS	Second	Nivolumab	2019	0·73 (0·6-0·98)236		0·67(0·4-1·05)/102		NSCLC
**Socinski**	(Socinski et al., 2018)	PFS	First line	Atezolizumab	2018	0·58(0·25-0·85)/585	-	0·80(0·30-1·30)/108	-	NSCLC
**Rittmeyer**	((Rittmeyer et al., 2017)	OS	Monotherapy	Atezolizumab	2017	0·74(0·61-0·88)/694	-	0·71(0·47-1·08)/156	-	NSCLC
**West**	(West et al., 2019) -1	PFS	Multitherapy	Atezolizumab	2019	0·81(0·65-1·02)/403	-	0·55(0·26-1·19)/48	0·79(0·64-0·98)/451	NSCLC
**West**	(West et al., 201	OS	Multitherapy	Atezolizumab	2019	0·64(0·53-0·77)/403	-	0·63(0·35-1·12)/48	0·64(0·54-0·77)/451	NSCLC

	9) - 2									
**Barlesi**	(Barlesi et al., 2018)	OS	Mon other apy	Avelumab	2018	0·83(0·66-1·04)/444	-	1·69(0·97-2·95)/84	-	NSCLC
**Antonia**	(Antonia et al., 2017)	PFS	Second or Third line	Durvalumab	2017	0·59(0·47-0·73)/649	-	0·29(0·15-0·57)/64	-	NSCLC
**Antonia**	(Antonia et al., 2018)	OS	Second or Third line	Durvalumab	2018	0·72(0·56-0·92)/649	-	0·35(0·16-0·76)/64	-	NSCLC
**Carbone**	(Carbone et al., 2017)-1	PFS	First line	Nivolumab	2017	1·03(0·66-1·62)/52	1·14(0·89-1·47)/186	2·51(1·31-4·83)/30	1·19(0·97-1·46)/271	NSCLC
**Carbone**	(Carbone et al., 2017)-2	OS	First line	Nivolumab	2017	1·05(0·63-1·74)/52	1·09(0·84-1·42)/186	1·02(0·54-1·93)/30	1·08(0·87-1·34)/271	NSCLC
**Reck**	(Reck et al., 2019)-1	OS/ABCP vs BCP	multi therapy	Atezolizumab	2019	0·80(0·65-0·98)/641	-	0·66(0·41-1·05)/159	-	NSCLC
**Reck**	(Reck et al., 201	OS/ACP vs BCP	multi therapy	Atezolizumab	2019	0·82(0·66-1·01)/648	-	0·96(0·62-1·49)/154	-	

	9) - 2									
**PD-1/PD-L1 (Other disease)**										
**Bellmunt**	(Bellmunt et al., 2017)	OS		Pembrolizumab	2019	0·32(0·15-0·68)/67	0·71(0·52-0·97)/284	1·06(0·72-1·55)/187	0·73(0·59-0·91)/542	urothelial carcinoma
**Cohen**	(Cohen et al., 2019)	OS		Pembrolizumab	2017	0·71(0·38-1·31)/68	0·78(0·60-1·02)/293	0·90(0·60-1·35)/134	0·80(0·65-0·98)/495	Head and Neck Cancer
**Ferris**	(Ferris et al., 2016)	OS		Nivolumab	2016	0·71(0·52-0·99)/276	-	0·58(0·32-1·06)/70	0·69(0·53-0·91)/361	Head and Neck Cancer
**Escudier**	(Escudier et al., 2017)	OS		Nivolumab	2017	0·79(0·60-1·03)/443	-	0·76(0·56-1·03)/355	-	Advanced Renal Cell Carcinoma
**Powles**	(Powles et al., 2018)-1	OS		Atezolizumab	2017	0·23(0·07-0·81)/30	1·14(0·77-1·70)/136	0·61(0·33-1·12)/67	0·81 (0·59-1·10)/234	urothelial carcinoma
**Powles**	(Powles et al., 2018)-2	OS		Atezolizumab	2017	0·69(0·44-1·06)/120	0·91(0·74-1·10)/546	0·80(0·60-1·06)/262	0·84 (0·72-0·97)/931	urothelial carcinoma

**Motzer**	(Motzer at al., 2019)-1	PFS		Avelumab	2019	0·58(0·42-0·82)/285	-	0·69(0·47-1·01)/274	0·63(0·49-0·81)/560	Advanced Renal Cell Carcinoma
**Motzer**	(Motzer at al., 2019)-2	PFS/Subgroup		Avelumab	2019	0·66(0·503-0·873)/449	-	0·71(0·531-0·951)/433	0·69(0·56-0·84)/886	Advanced Renal Cell Carcinoma
**CTLA-4**										
**Govindan**	(Govindan et al., 2017)	OS		Ipilimumab	2017	0·88(0·73-1·05)/339	-	1·19(0·71-1·99)/44		NSCLC
**Reck**	(Reck et al., 2016)	OS		Ipilimumab	2016	1·09(0·89-1·32)/268	-	1·02(0·80-1·30)/172		ED-SCLC (not NSCLC)
**Hellmann**	(Hellmann et al., 2018)	PFS		Nivolumab plus ipilimumab	2018	0·57(0·42-0·78)/130	-	0·58(0·43-0·77)/9	0·58(0·43-0·77)/139	NSCLC
**Hellmann**	(Hellmann et al., 2019)	OS		Nivolumab plus ipilimumab	2019	0·77(0·64-0·92)/674	-	1·23(0·76-1·98)/107		NSCLC

**Scherpereel**	(Scherpereel et al., 2019)-1	OS/Nivolumab		Nivolumab	2019	1·00(1·00-1·00)/ 34	-	0·90(0·50-2·00)/ 29	total number of patients less than 100	Malignant pleural mesothelioma (not NSCLC)
**Scherpereel**	(Scherpereel et al., 2019)-2	OS/nivolumab plus ipilimumab		nivolumab plus ipilimumab	2019	1·00(1·00-1·00)/ 36	-	1·30(0·60-2·50)/ 26	total number of patients less than 100	
**MUC1**										
**Butts**	(Butts et al., 2014)-1	OS		tecemotide	2013	0·75(0·61-0·92)/762	-	1·51(0·64-3·57)/ 44		
**Butts**	(Butts et al., 2014)-2	OS		tecemotide	2013	1·07(0·83-1·39)/402	-	4·90(0·93-26·00)/ 31		
**Katakami**	(Katakami et al., 2017)	OS		tecemotide	2017	0·97(0·61-1·57)/115	-	1·07(0·30-3·80)/ 17		
**VEGF**										
**Zalcman**	(Zalcman et al., 2016)	OS		bevacizumab	2015	0·81(0·61-1·08)/125	-	0·73 (0·53–1·02)/ 98	0·77 (0·62-0·95)/223	advanced malignant pleural mesothelioma

**Saito**	(Saito et al., 2019)	PFS		bevacizumab	2019	0·63(0·35-1·11)/41	2·94(0·30-28·5)/6	0·54(0·33-0·90)/65	0·63(0·43-0·91)/112	EGFR-positive advanced non-squamous non-small-cell lung cancer
**Scagliotti**	(Scagliotti et al., 2012)-1	Os		bevacizumab	2012	0·944(0·805-1·106)/774	-	0·870(0·606-1·248)/186	0·927(0·801-1·072)/960	advanced non-small-cell lung cancer
**Scagliotti**	(Scagliotti et al., 2012)-2	PFS		bevacizumab	2012	0·813(0·690-0·958)/774	-	0·911(0·650-1·27·)/186	0·825(0·712-0·956)/960	advanced non-small-cell lung cancer
**Seto**	(Seto et al., 2020)	OS		bevacizumab	2020	0·953(0·778-1·168)/236	-	0·734(0·491-1·098)/59	-	Advanced Nonsquamous Non-Small-Cell Lung Cancer
**Baggstrom**	(Baggstrom et al., 2017)-1	PFS		bevacizumab	2017	1·26(0·68-2·34)/25	0·97(0·54-1·76)/76	1·00(1·00-1·00)/5	-	Advanced-Stage IIIB/IV Non-Small Cell Lung Cancer
**Baggstrom**	(Baggstrom et al., 2017)-2	OS		bevacizumab	2017	113(0·59-2·18)/25	1·24(0·67-2·33)/76	1·00(1·00-1·00)/5	-	Advanced-Stage IIIB/IV Non-Small Cell Lung Cancer

**Herbst**	(Herbst et al., 2011)	OS		bevacizumab	2011	1·06 (0·87-1·29)/285	-	0·44(0·21-0·94)/34	0·97 (0·80-1·18)/319	advanced non-small-cell lung cancer
**Johnson**	(Johnson et al., 2013)	PFS		bevacizumab	2013	0·74(0·54-1·01)/129	0·79(0·61-1·03)/178	0·34(0·19-0·61)/66	-	advanced non-small-cell lung cancer
**Zhou**	(Zhou et al., 2015)	PFS		bevacizumab	2015	0·44(0·28-0·69)/130	-	0·34(0·22-0·52)/146	0·40(0·29-0·54)/276	Advanced or Recurrent Nonsquamous Non-Small-Cell Lung Cancer
**Zinner**	(Zinner et al., 2015)-1	PFS		bevacizumab	2015	0·85(0·60-1·10)/336	-	0·60(0·20-2·70)/23	0·80(0·65-1·10)/361	advanced nonsquamous non-small-cell lung cancer
**Zinner**	(Zinner et al., 2015)-2	OS		bevacizumab	2015	1·05(0·85-1·40)/336	-	0·50(0·20-2·40)/23	1·10(0·75-1·40)/361	advanced nonsquamous non-small-cell lung cancer
**Barlesi**	(Barlesi et al., 2014)-1	PFS		bevacizumab	2014	0·62(0·45-0·84)/?	-	0·42(0·24-0·74)/?	-	advanced nonsquamous nonsmall-cell lung cancer
**Barlesi**	(Barlesi et	OS		bevacizumab	2014	0·85(0·50-1·21)/?	-	0·73(0·32-1·66)/?	-	advanced nonsquamous

	al., 201 4)-2									nonsmall-cell lung cancer

Part A. Data collection and risk of bias analysis

PROSPERO registration number: CRD42019146402
Protocol Publication: Medicine MS# MD-D-19-08502_R2
Risk of bias was assessed for gender, age, histology, ECOG performance status, randomization, ethnic groups/geographic, phase of clinical trial, and smoking status, etc.

Supplemental Tabulation s1. Based characterization for bias assessment for PD-1/PD-L1 drugs

First author	Ref. No.	Gender		
		Male/#Patients		Female/#Patients
Barlesi	[2]	0.83(0.64-1.08)/367		1.08(0.74-1.59)/162
Mok	[4]-1	0.68(0.53-0.88)/415		0.78(0.53-1.15)/184
Mok	[4]-2	0.71(0.57-0.88)/568		1.01(0.72-1.41)/250
Mok	[4]-3	0.80(0.68-0.94)/902		0.89(0.68-1.17)/372
Reck	[32]	0.39(0.26-0.58)/187		0.75(0.46-1.21)/118
Gandhi	[33]-1	0.70(0.50-0.99)/363		0.29(0.19-0.44)/253
Gandhi	[33]-2	0.66(0.50-0.87)/363		0.40(0.29-0.54)/253
Borghaei	[37]	0.73(0.56-0.96)/319		0.78(0.58-1.04)/263
Wu	[39]	0.71(0.50-0.90)/397		0.74(0.40-1.26)/107
Carbone	[41]-1	1.05(0.81-1.37)/332		1.36(0.98-1.90)/209
Carbone	[41]-2	0.97(0.74-1.26)/332		1.15(0.79-1.66)/209
Socinski	[42]	0.55(0.42-0.67)/425		0.73(0.55-0.98)/267
Rittmeyer	[43]	0.79(0.64-0.97)/520		0.64(0.49-0.85)/330
West	[44]-1	0.87(0.66-1.15)/400		0.66(0.46-0.93)/279
West	[44]-2	0.67(0.54-0.85)/400		0.59(0.45-0.78)/279
Reck	[45]-1	0.73(0.57-0.93)/479		0.82(0.61-1.12)/321
Reck	[45]-2	0.82(0.64-1.04)/480		0.88(0.65-1.19)/322

Antonia	[46]	0.56(0.44-0.71)/500		0.54(0.37-0.79)/213
Antonia	[47]	0.78(0.59-1.03)/500		0.46(0.30-0.73)/213
Govindan	[18]	0.85(0.71-1.02)/635		1.33(0.84-2.11)/114
Reck	[19]	1.07(0.89-1.28)/643		1.06(0.81-1.37)/311
Hellmann	[20]	0.52(0.36-0.74)/204		0.70(0.41-1.20)/ 95
Hellmann	[21]	0.75(0.61-0.93)/515		0.91(0.69-1.21)/278
Scherpereel	[22]-1	0.80(0.30-2.00)/ 47		1.00(0.30-1.70)/ 16
Scherpereel	[22]-2	0.60(0.30-1.50)/ 53		1.00(0.30-1.70)/ 9

**First author**	**Ref. No.**	ECOG		
		**0**		**1**
Barlesi	[2]	0.73(0.50-1.08)/187		0.99(0.77-1.28)/349
Mok	[4]-1	0.57(0.37-0.86)/187		0.74(0.58-0.95)/412
Mok	[4]-2	0.71(0.49-1.01)/253		0.80(0.65-0.98)/565
Mok	[4]-3	0.77(0.59-1.05)/390		0.83(0.71-0.98)/884
Reck	[32]	0.45(0.26-0.77)/107		0.51(0.35-0.73)/197
Gandhi	[33]-1	0.44(0.28-0.71)/266		0.53(0.39-0.73)/346
Gandhi	[33]-2	0.49(0.35-0.68)/266		0.56(0.43-0.72)/346
Borghaei	[37]	0.64(0.44-0.93)/179		0.80(0.63-1.00)/402
Wu	[39]	1.01(0.50-2.00)/68		0.69(0.52-0.90)/435
Carbone	[41]-1	1.69(1.18-2.42)/178		1.01(0.79-1.30)/362
Carbone	[41]-2	1.11(0.74-1.66)/178		1.02(0.79-1.32)/362
Socinski	[42]	0.55(0.40-0.73)/282		0.64(0.50-0.80)/404
Rittmeyer	[43]	0.78(0.58-1.04)/315		0.68(0.56-0.84)/535
West	[44]-1	0.85(0.59-1.22)/280		0.77(0.58-1.00)/397
West	[44]-2	0.59(0.44-0.78)/280		0.68(0.54-0.86)/397
Reck	[45]-1	0.75(0.53-1.07)/338		0.75(0.59-0.94)/456
Reck	[45]-2	0.85(0.61-1.18)/359		0.84(0.67-1.06)/440
Antonia	[46]	-		-
Antonia	[47]	-		-
Govindan	[18]	0.99(0.73-1.33)/259		0.86(0.70-1.05)/485
Reck	[19]	1.28(0.98-1.69)/284		0.99(0.83-1.18)/668
Hellmann	[20]	0.62(0.38-1.02)/105		0.55(0.38-0.80)/192
Hellmann	[21]	0.66(0.48-0.89)/269		0.89(0.73-1.09)/510
Scherpereel	[22]-1	-		-
Scherpereel	[22]-2	-		-

**First author**	**Ref. No.**	Histology		
		**Squamous**		**Non-Squamous**
Barlesi	[2]	0.70(0.48-1.01)/180		1.02(0.79-1.33)/349
Mok	[4]-1	0.53(0.38-0.75)/221		0.82(0.63-1.07)/378
Mok	[4]-2	0.65(0.49-0.87)/304		0.85(0.68-1.08)/514
Mok	[4]-3	0.75(0.60-0.93)/492		0.86(0.72-1.03)/782
Reck	[32]	0.35(0.17-0.71)/ 56		0.55(0.39-0.76)/249
Gandhi	[33]-1	-		-
Gandhi	[33]-2	-		-
Borghaei	[37]	-		-
Wu	[39]	0.61(0.40-0.90)/200		0.76(0.60-1.05)/304
Carbone	[41]-1	0.83(0.54-1.26)/129		1.29(1.02-1.63)/412
Carbone	[41]-2	0.82(0.54-1.24)/129		1.17(0.91-1.52)/412
Socinski	[42]	-		-
Rittmeyer	[43]	0.73(0.54-0.98)/222		0.73(0.60-0.89)/628
West	[44]-1	-		-
West	[44]-2	-		-
Reck	[45]-1	-		-
Reck	[45]-2	-		-
Antonia	[46]	0.68(0.50-0.92)/326		0.45(0.33-0.59)/387
Antonia	[47]	0.72(0.52-0.99)/326		0.61(0.44-0.86)/387
Govindan	[18]	-		-
Reck	[19]	-		-
Hellmann	[20]	0.63(0.39-1.04)/100		0.55(0.38-0.80)/199
Hellmann	[21]	0.69(0.52-0.92)/236		0.85(0.69-1.04)/557
Scherpereel	[22]-1	-		-
Scherpereel	[22]-2	-		-

**First author**	**Ref. No.**	Age		
		**>=75**	**65-75**	**<65**
Barlesi	[2]	0.98(0.71-1.34)/250		0.84(0.63-1.13)/279
Mok	[4]-1	0.58(0.42-0.80)/271		0.81(0.60-1.08)/328
Mok	[4]-2	0.71(0.54-0.92)/378		0.84(0.65-1.08)/440
Mok	[4]-3	0.82(0.66-1.01)/567		0.81(0.67-0.98)/707
Reck	[32]	0.45(0.29-0.70)/164		0.61(0.40-0.92)/141
Gandhi	[33]-1	0.64(0.43-0.95)/304		0.43(0.21-0.61)/312
Gandhi	[33]-2	0.75(0.55-1.02)/304		0.43(0.32-0.56)/312
Borghaei	[37]	0.90(0.43-1.87)/ 43	0.63(0.45-0.89)/200	0.81(0.62-1.04)/339

Wu	[39]	0.50(0.20-0.76)/127		0.76(0.60-1.00)/377
Carbone	[41]-1	1.21(0.91-1.62)/260		1.17(0.88-1.56)/281
Carbone	[41]-2	1.04(0.77-1.41)/260		1.13(0.83-1.54)/281
Socinski	[42]	0.78(0.42-1.50)/ 64	0.52(0.35-0.67)/248	0.65(0.52-0.80)/375
Rittmeyer	[43]	0.66(0.52-0.83)/397		0.80(0.64-1.00)/453
West	[44]-1	0.78(0.58-1.05)/338		0.79(0.58-1.08)/341
West	[44]-2	0.64(0.50-0.82)/338		0.64(0.50-0.82)/341
Reck	[45]-1	0.94(0.50-1.76)/ 72	0.69(0.49-0.96)/281	0.78(0.60-1.00)/441
Reck	[45]-2	0.83(0.41-1.65)/ 65	0.97(0.71-1.32)/284	0.76(0.59-0.98)/449
Antonia	[46]	0.74(0.54-1.01)/322		0.43(0.32-0.57)/391
Antonia	[47]	0.76(0.55-1.06)/322		0.62(0.44-0.86)/391
Govindan	[18]	0.85(0.51-1.43)/ 71	1.06(0.81-1.37)/298	0.82(0.64-1.04)/380
Reck	[19]	0.70(0.40-1.20)/ 72	1.14(0.87-1.49)/306	1.08(0.90-1.31)/576
Hellmann	[20]	0.42(0.14-1.30)/ 27	0.62(0.40-0.97)/143	0.51(0.34-0.77)/156
Hellmann	[21]	0.92(0.57-1.48)/ 81	0.91(0.70-1.19)/306	0.70(0.55-0.89)/406
Scherpereel	[22]-1	-		-
Scherpereel	[22]-2	-		-

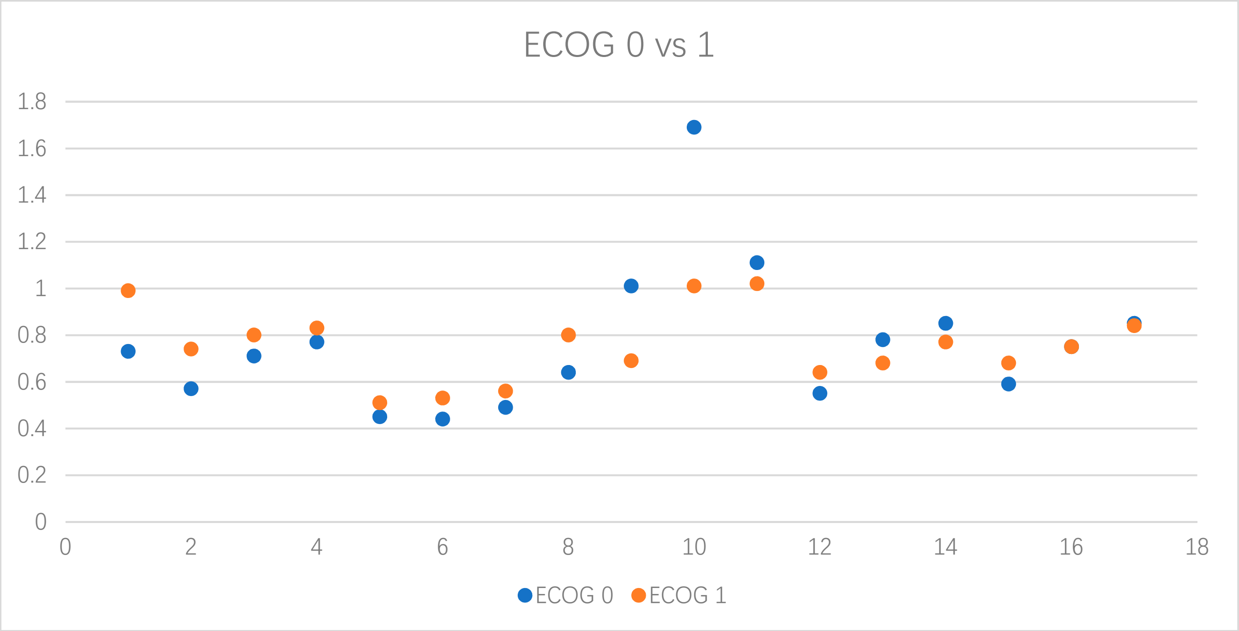

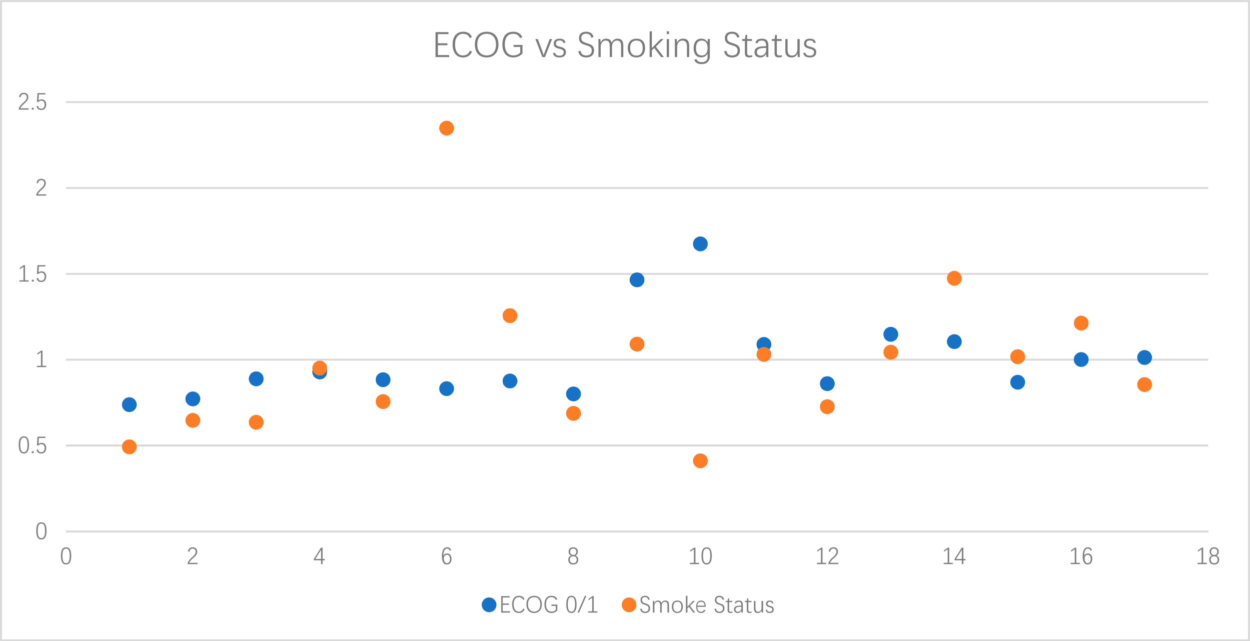

Supplemental Figure s1. No obvious influence of ECOG types on the OS and PFS ratio of patients of smoker and non-smokers.

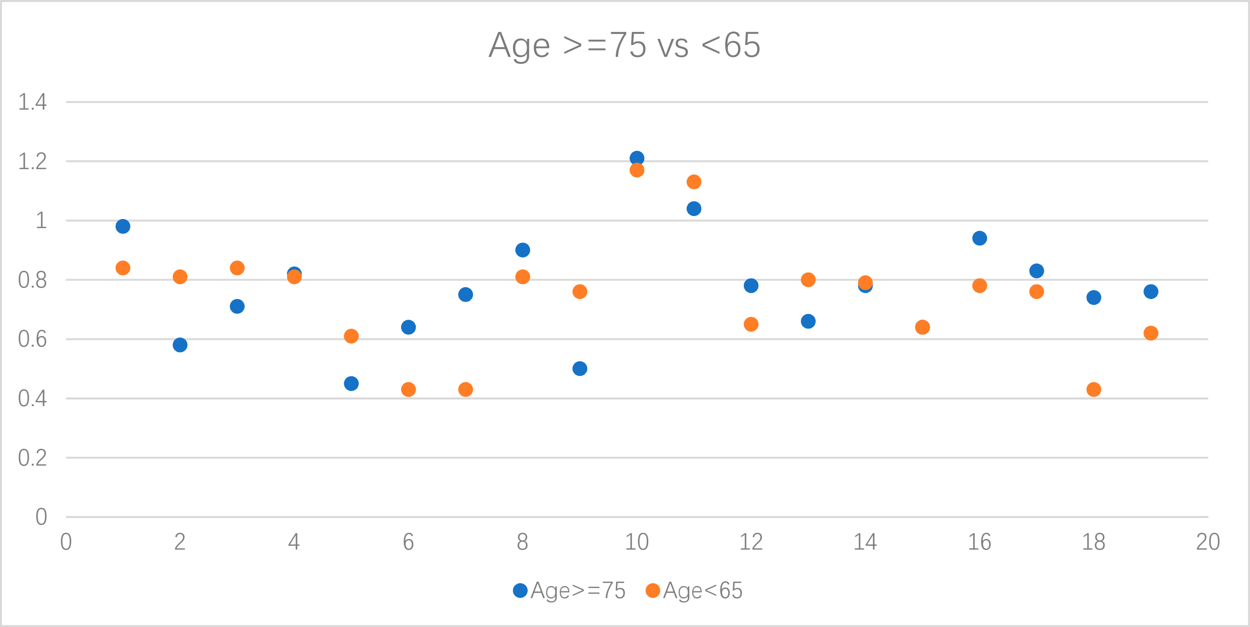

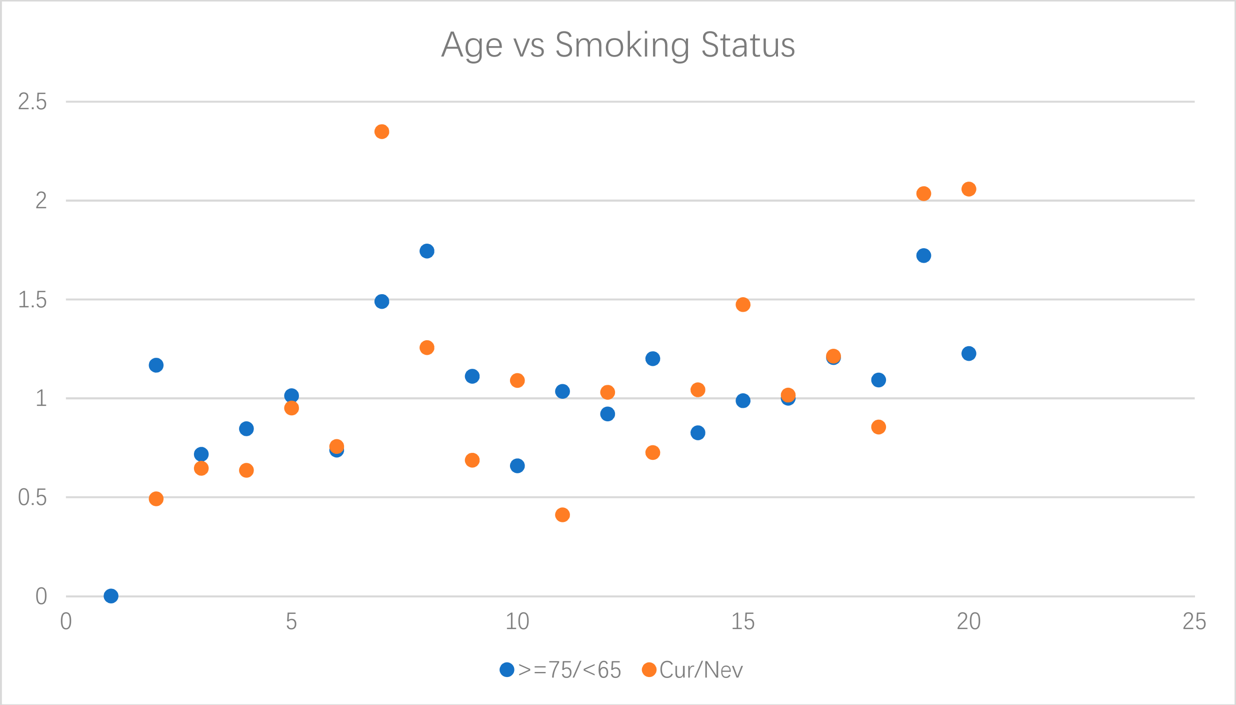

Supplemental Figure s2. Influence of age on the OS and PFS ratio of patients of smoker and non-smokers.

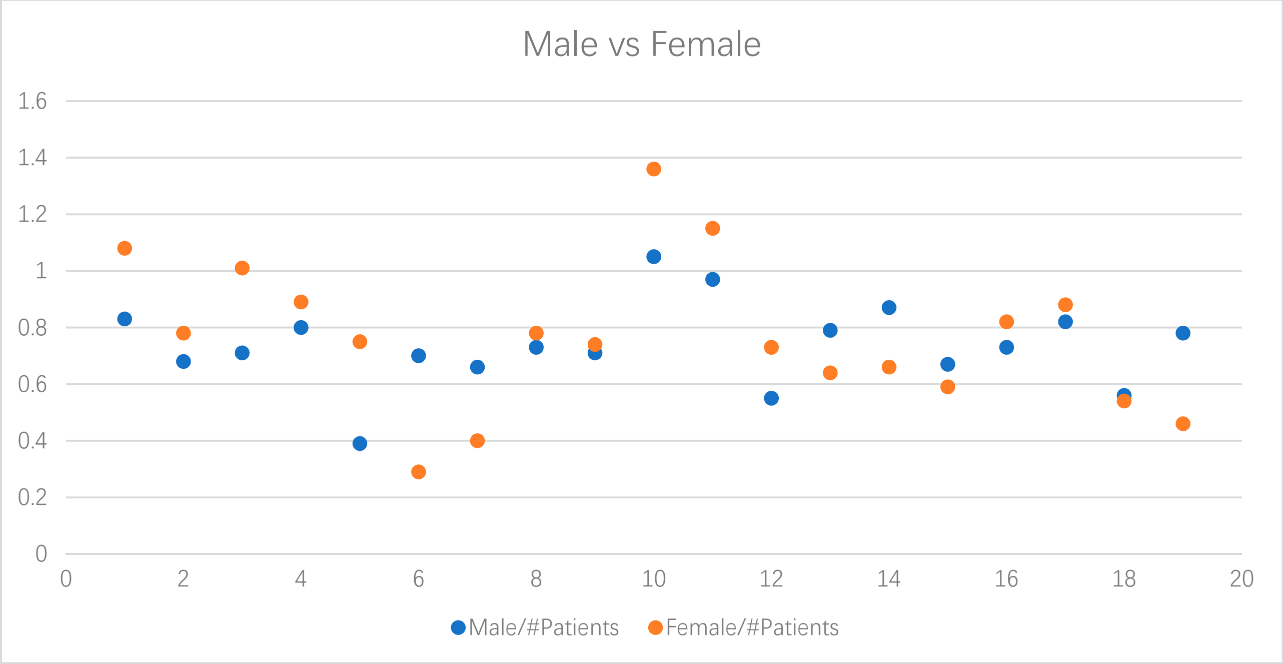

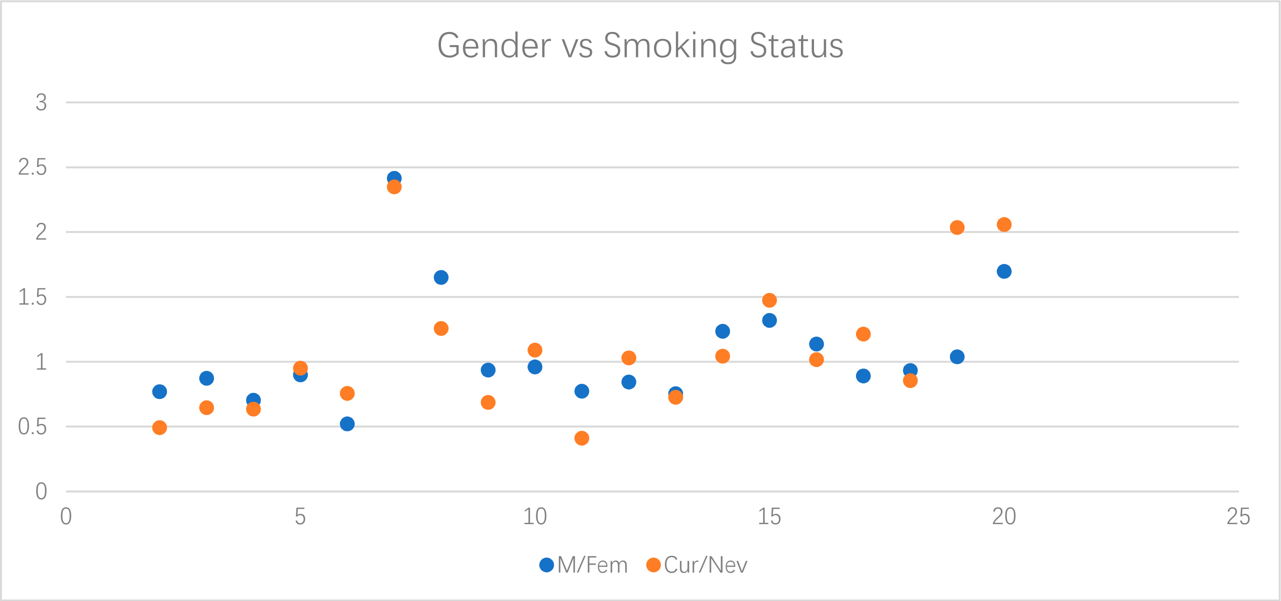

Supplemental Figure s3. No obvious influence of gender on the OS and PFS ratio of patients of smoker and non-smokers.

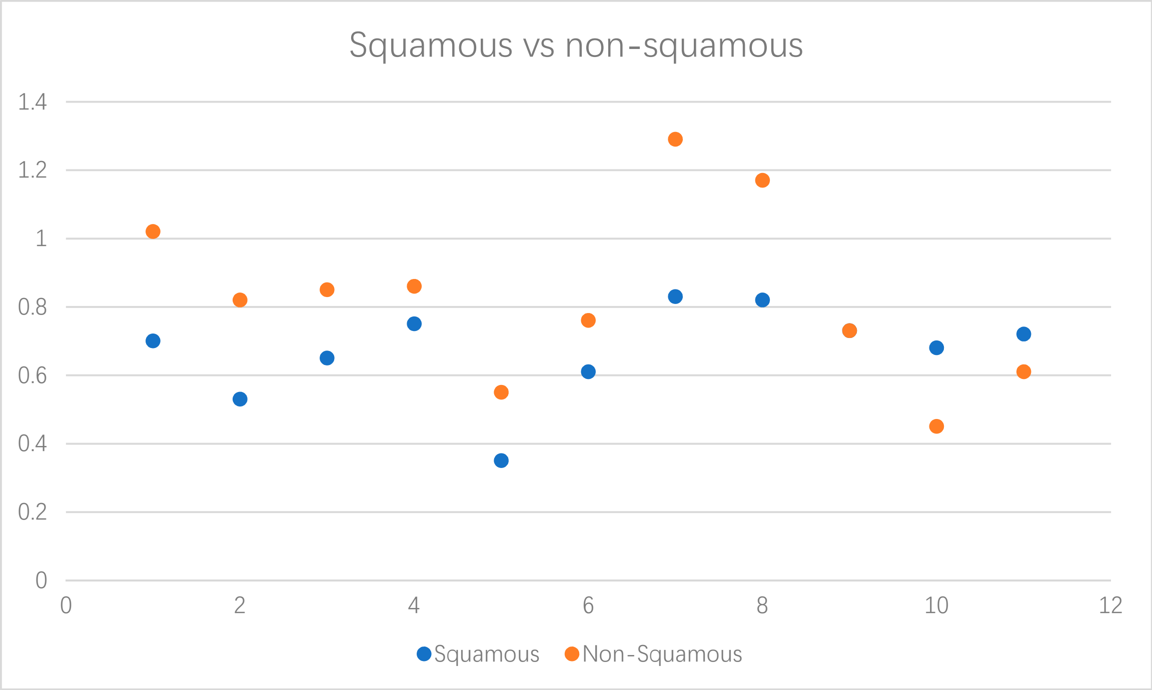

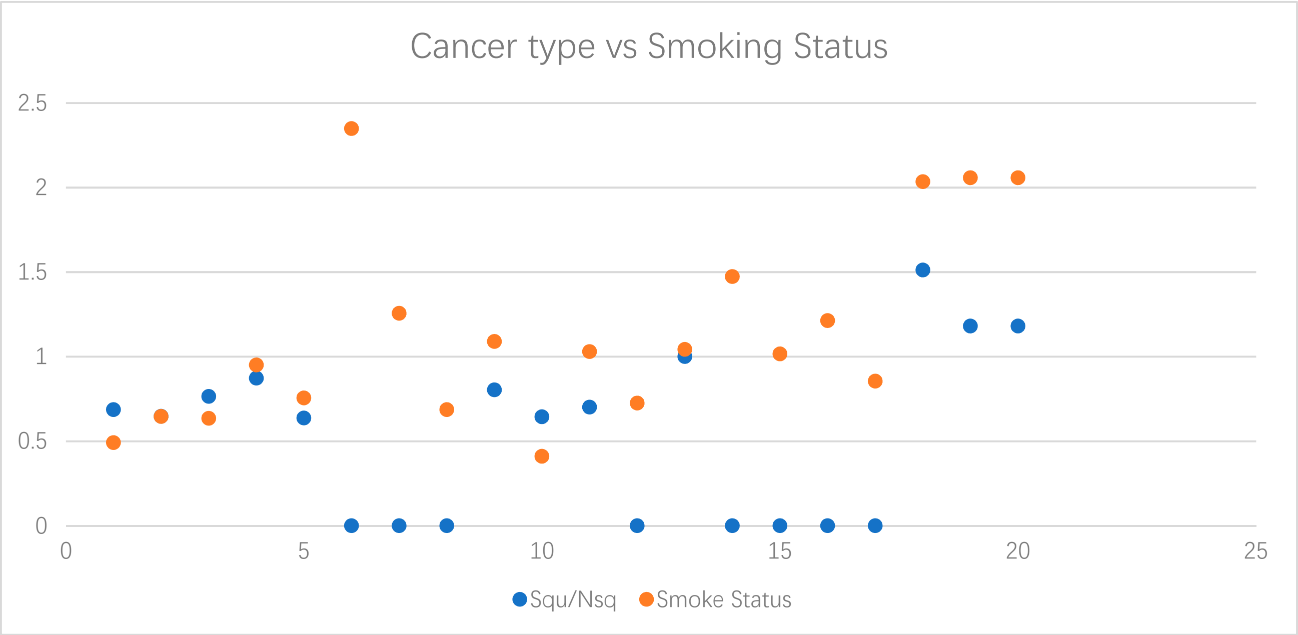

Supplemental Figure s4. No obvious influence of Histology types (Squamous, non-squamous) on the OS and PFS ratio of patients of smoker and non-smokers

Supplemental Tabulation s2. Additional characterization of clinical trials for bias assessment

First author	Ref. No.	ANALYTIC METRIX	first/second	Drugs	year
Barlesi	[2]	OS	Monotherapy	Avelumab	2018
Mok	[4]-1	OS/TPS≥50%	First line	Pembrolizumab	2019
Mok	[4]-2	OS/TPS≥20%	First line	Pembrolizumab	2019
Mok	[4]-3	OS/TPS≥1%	First line	Pembrolizumab	2019
Reck	[32]	PFS	First line	Pembrolizumab	2016
Gandhi	[33]-1	OS	Multitherapy	Pembrolizumab	2016
Gandhi	[33]-2	PFS	Multitherapy	Pembrolizumab	2016
Borghaei	[37]	OS	Monotherapy	Nivolumab	2015
Wu	[39]	OS	Second	Nivolumab	2019
Carbone	[41]-1	PFS	First line	Nivolumab	2017
Carbone	[41]-2	OS	First line	Nivolumab	2017
Socinski	[42]	PFS	First line	Atezolizumab	2018
Rittmeyer	[43]	OS	Monotherapy	Atezolizumab	2017
West	[44]-1	Os	Multitherapy	Atezolizumab	2019
West	[44]-2	PFS	Multitherapy	Atezolizumab	2019
Reck	[45]-1	OS/ABCP vs BCP	multitherapy	Atezolizumab	2019
Reck	[45]-2	OS/ACP vs BCP	multitherapy	Atezolizumab	2019
Antonia	[46]	PFS	Second or Third line	Durvalumab	2017
Antonia	[47]	OS	Second or Third line	Durvalumab	2018
**first author**	**Ref. No.**	**ANALYTIC METRIX**	**Other cancer**	**Drugs**	**year**
Bellmunt	[48]	OS		Pembrolizumab	2019
Cohen	[49]	OS		Pembrolizumab	2017
Ferris	[50]	OS		Nivolumab	2016
Escudier	[51]	OS		Nivolumab	2017
Powles	[52]-1	OS Subgroup		Atezolizumab	2017
Powles	[52]-2	OS		Atezolizumab	2017
Motzer	[53]-1	PFS Subgroup		Avelumab	2019
Motzer	[53]-2	PFS		Avelumab	2019
**first author**	**Ref. No.**	**ANALYTIC METRIX**		**Drugs**	**year**
Govindan	[18]	OS		Ipilimumab	2017
Reck	[19]	OS		Ipilimumab	2016
Hellmann	[20]	PFS		Nivolumab plus ipilimumab	2018

Hellmann	[21]	OS		Nivolumab plus ipilimumab	2019
Scherpereel	[22]-1	OS/Nivolumab		Nivolumab	2019
Scherpereel	[22]-2	OS/nivolumab plus ipilimumab		nivolumab plus ipilimumab	2019
**first author**	**Ref. No.**	**ANALYTIC METRIX**		**Drugs**	**year**
Butts	[25]-1	OS		tecemotide	2013
Butts	[25]-2	OS		tecemotide	2013
Katakami	[26]	OS		tecemotide	2017
**first author**	**Ref. No.**	**ANALYTIC METRIX**		**Drugs**	**year**
Zalcman	[59]	OS		bevacizumab when added to the present standard of care, cisplatin plus pemetrexed	2015
Saito	[60]	PFS		Erlotinib plus bevacizumab versus erlotinib alone	2019
Scagliotti	[61]-1	Os		Sunitinib plus erlotinib	2012
Scagliotti	[61]-2	PFS		Sunitinib plus erlotinib	2012
Seto	[62]	OS		Maintenance Bevacizumab With or Without Pemetrexed	2020
Baggstrom	[63]-1	PFS		Maintenance Sunitinib	2017
Baggstrom	[63]-2	OS		Maintenance Sunitinib	2017
Herbst	[64]	OS		bevacizumab plus erlotinib versus erlotinib alone	2011
Johnson	[65]	PFS		bevacizumab	2013

Zhou	[66]	PFS		Carboplatin/Paclitaxel Plus Bevacizumab	2015
Zinner	[67]-1	PFS		Pac+Cb+Bev	2015
Zinner	[67]-2	OS		Pac+Cb+Bev	2015

Supplemental Tabulation s3. Additional characterization of clinical trials for bias assessment for drugs of anti-PD-1 with anti-CTLA4.

**Category**	first author		Hellmann	Hellmann	Scherpereel
	**Ref. No.**		**[20]**	**[21]**	**[22]-2**
	ANALYTIC METRIX		PFS	OS	OS/nivolumab plus ipilimumab
	first/second				
	Drugs		Nivolumab plus ipilimumab	Nivolumab plus ipilimumab	nivolumab plus ipilimumab
	year		2018	2019	2019
	Current/#Patients		0.57(0.42-0.78)/276	0.77(0.64-0.92)/674	1.00(0.60-1.40)/ 36
Smoking Status	Former/#Patients		-	-	-
	Never/#Patients		0.58(0.43-0.77)/ 23	1.23(0.76-1.98)/107	1.30(0.60-2.50)/ 26
	Overall/#Patients		0.58(0.43-0.77)/299	0.79(0.65-0.96)/793	-
	Male/#Patients		0.52(0.36-0.74)/204	0.75(0.61-0.93)/515	0.60(0.30-1.50)/ 53
Gender					
	Female/#Patients		0.70(0.41-1.20)/ 95	0.91(0.69-1.21)/278	1.00(0.30-1.70)/ 9
	>=75		0.42(0.14-1.30)/ 27	0.92(0.57-1.48)/ 81	-
Age	65-75		0.62(0.40-0.97)/143	0.91(0.70-1.19)/306	
	<65		0.51(0.34-0.77)/156	0.70(0.55-0.89)/406	-
	Squamous		0.63(0.39-1.04)/100	0.69(0.52-0.92)/236	-
Histology					
	Non-Squamous		0.55(0.38-0.80)/199	0.85(0.69-1.04)/557	-
	0		0.62(0.38-1.02)/105	0.66(0.48-0.89)/269	-
ECOG					
	1		0.55(0.38-0.80)/192	0.89(0.73-1.09)/510	-

	Authors		Hellmann MD, Ciuleanu TE, Pluzanski A, Lee JS, Otterson GA, Audigier-Valette C, Minenza E, Linardou H, Burgers S, Salman P, Borghaei H, Ramalingam SS, Brahmer J, Reck M, O'Byrne KJ, Geese WJ, Green G, Chang H, Szustakowski J, Bhagavatheeswaran P, Healey D, Fu Y, Nathan F, Paz-Ares L.	Hellmann MD, Paz-Ares L, Bernabe Caro R, Zurawski B, Kim SW, Carcereny Costa E, Park K, Alexandru A, Lupinacci L, de la Mora Jimenez E, Sakai H, Albert I, Vergnenegre A, Peters S, Syrigos K, Barlesi F, Reck M, Borghaei H, Brahmer JR, O'Byrne KJ, Geese WJ, Bhagavatheeswaran P, Rabindran SK, Kasinathan RS, Nathan FE, Ramalingam SS. Nivolumab plus Ipilimumab in Advanced Non-Small-Cell Lung Cancer	
	Titles		Nivolumab plus Ipilimumab in Lung Cancer with a High Tumor Mutational Burden.	Nivolumab plus Ipilimumab in Advanced Non-Small-Cell Lung Cancer	
	category		NSCLC	NSCLC	

PART B. Article information Questionnaires

Email title: Information regarding your publication: "Journal name, Year, Page numbers".
Email set: Highly important
Email content:
Dear Dr. XXXXX
I am writing to you, on behalf the investigator group on smoking status and response to drug treatment of cancer patients, for potential additional information from your publication titled " XXXXXXX". We would appreciate very much if you could provide the hazard ratio of patients with different smoking status including the range and possibly the P values. Please also let us know whether the data could be a duplicates of other related publications.
The information will be used in a manuscript of review and meta-analysis for cancer drugs. You will be acknowledged for your information in the paper, or be as a co-author if you desire and have time to assist on the manuscript, whatever you prefer.
In considering the days of manuscript preparation, we would appreciate very much if you respond immediate on the availability of such a data and send the data before or on the end of two weeks from the date of this email.
An example of information is provided below for your consideration

Supplemental Tabulation s4. Example of data confirmation with authors.

Drugs	Study (First Author)/analytic Metrix	PD-1 Positive	Current smokers/ #Patients	Former smokers /# Patients	Never smoke /# Patients	Overall/# Patients	Note

**Pembrolizumab**	Reck/ versus Chemotherapy /HR for Disease progression or death [20]	All are PD-L1 expression on at least 50% of tumor cells	0.68 (0.36-1.31)/65	0.47 (0.33-0.67) /216	0.90 (0.11-7.59)/24	0.50 (0.37-0.68)/305	First line treatment / Open-label/Randomly assigned to 305 patients

Email sending days:
Initial sending date: July 18, 2019;
Reminding date: July 28, 2019.
Kindly remind the previous email regarding information in your publication " ".

PART C. Comparison response to treatment between smokers and non-smokers in cancer patients treated with anti-PD-1/PD-L1, CTLA-4 and MUC1 drugs.

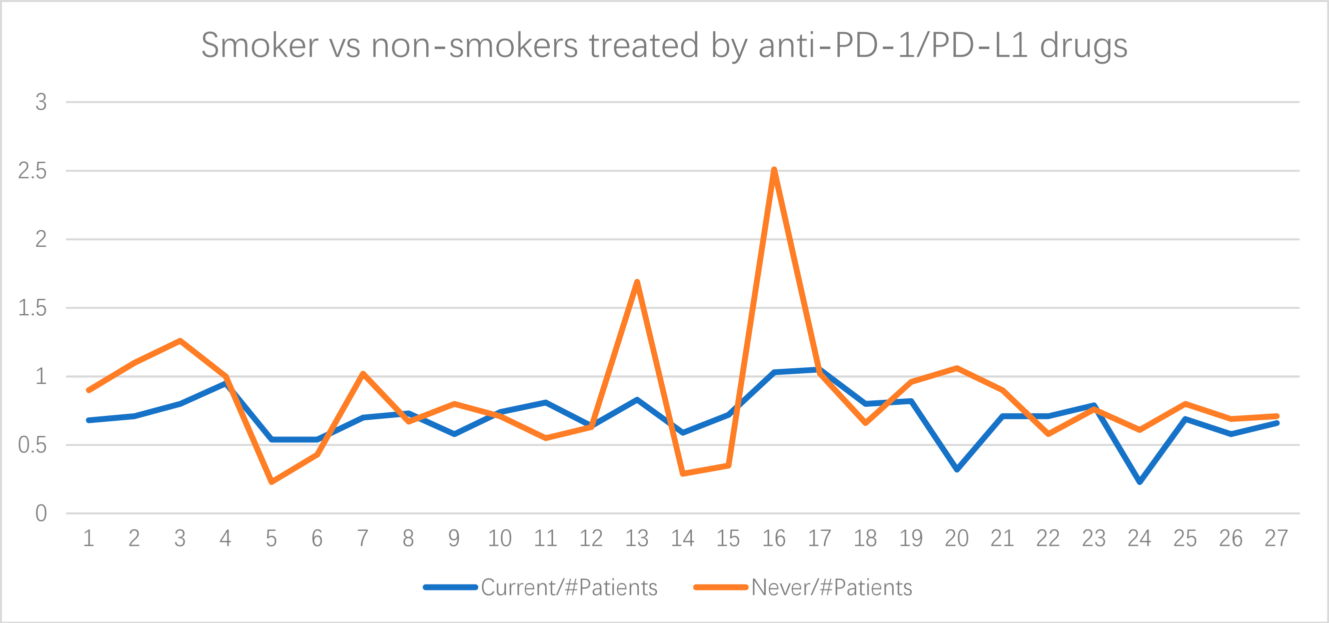

Supplemental Figure s5. Response to treatment by anti-PD-1/PD-L1 drugs between smoker and non-smokers.

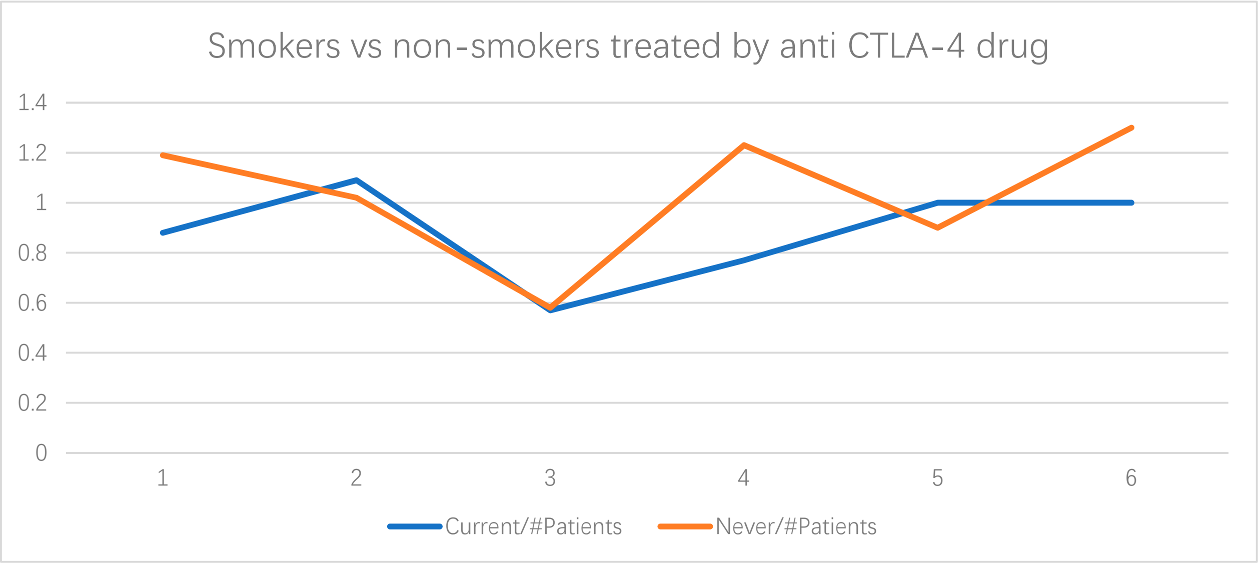

Supplemental Figure s6. Response to treatment by anti-CTLA-4 drug between smoker and non-smokers.

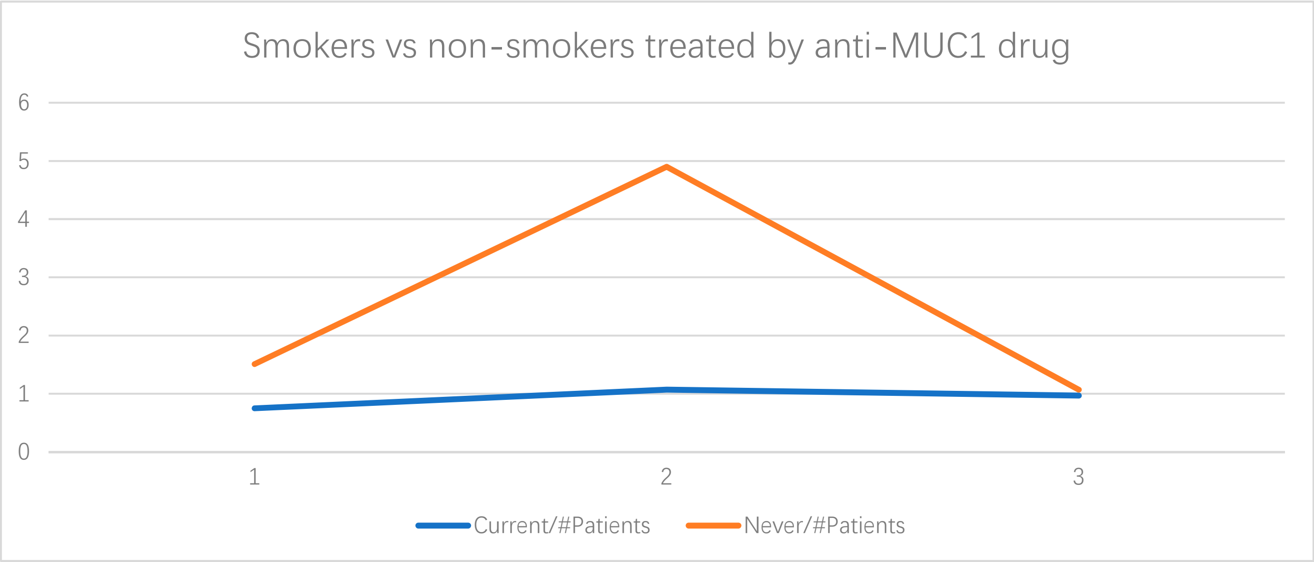

Supplemental Figure s7. Response to treatment by anti-MUC1 drug between smoker and non-smokers.

PART D. Meta-analysis data on subgroups of NSCLC patients treated with anti-PD-1/PD-L1, CTLA-4 and MUC1 drugs

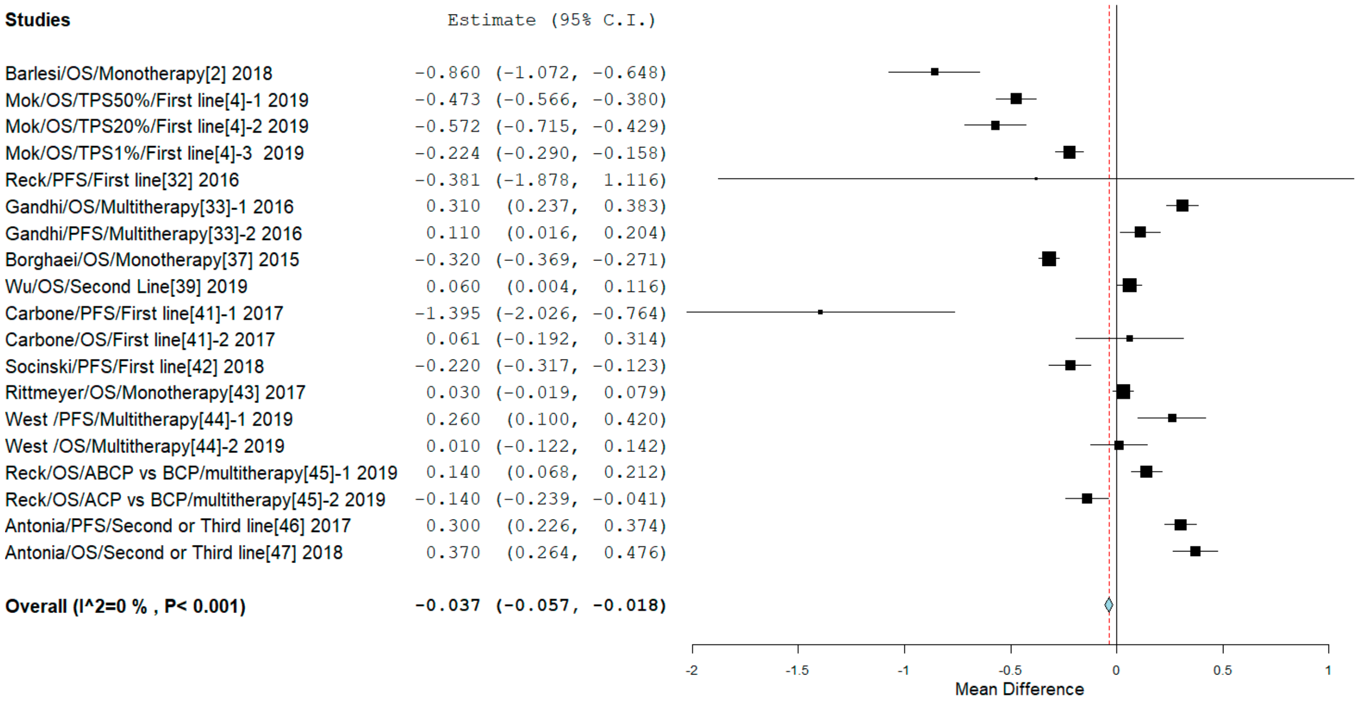

Supplemental Figure s8. Response to treatment by anti-PD-1/PD-L1drugs between smoker and non-smokers using fixed effect (FE) model.

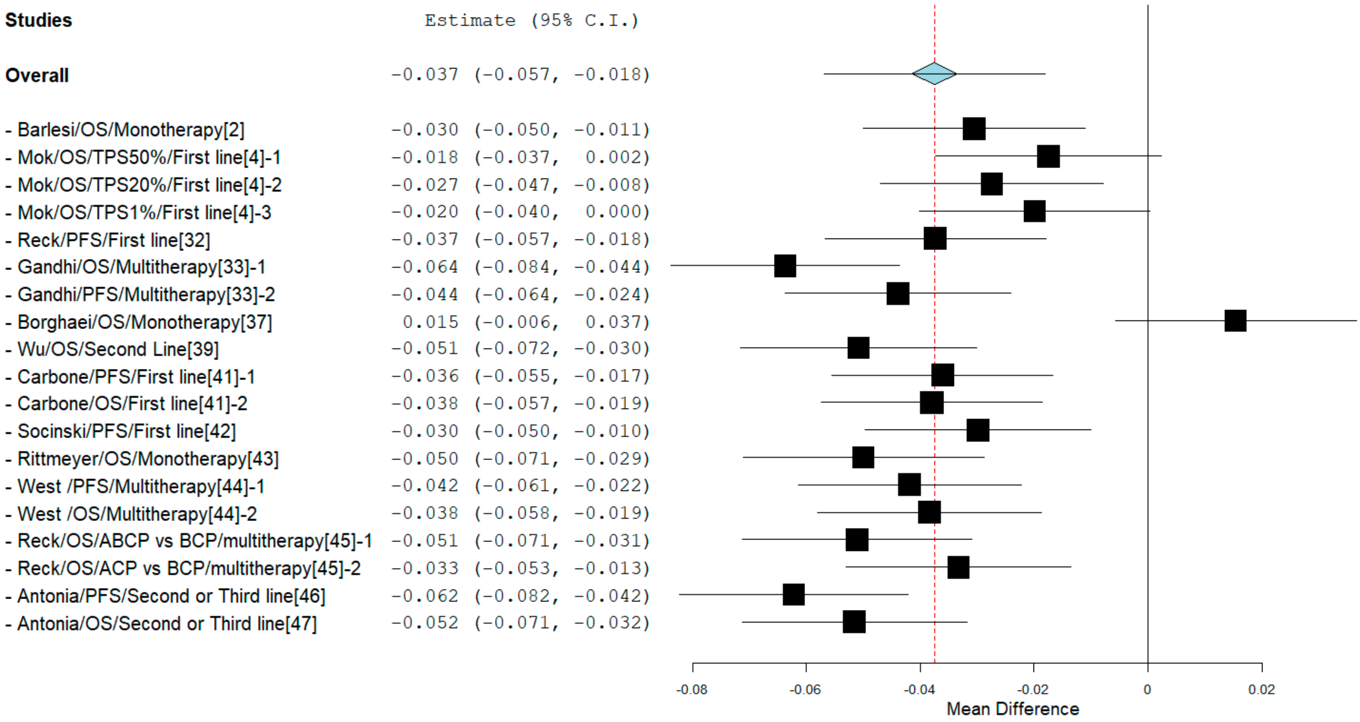

Supplemental Figure s9. Response to treatment by anti-PD-1/PD-L1drugs between smoker and non-smokers using FE leaveoneout model.

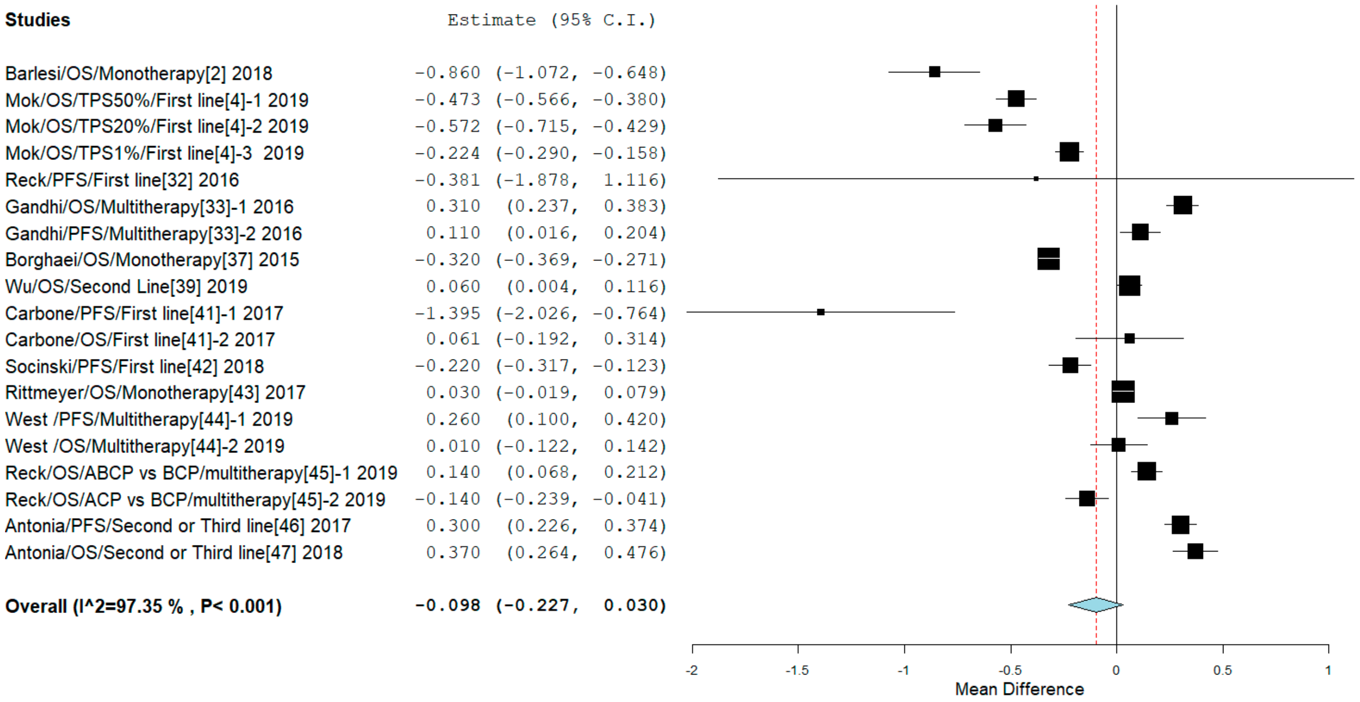

Supplemental Figure s10. Response to treatment by anti-PD-1/PD-L1drugs between smoker and non-smokers using randomized effect (RE) model.

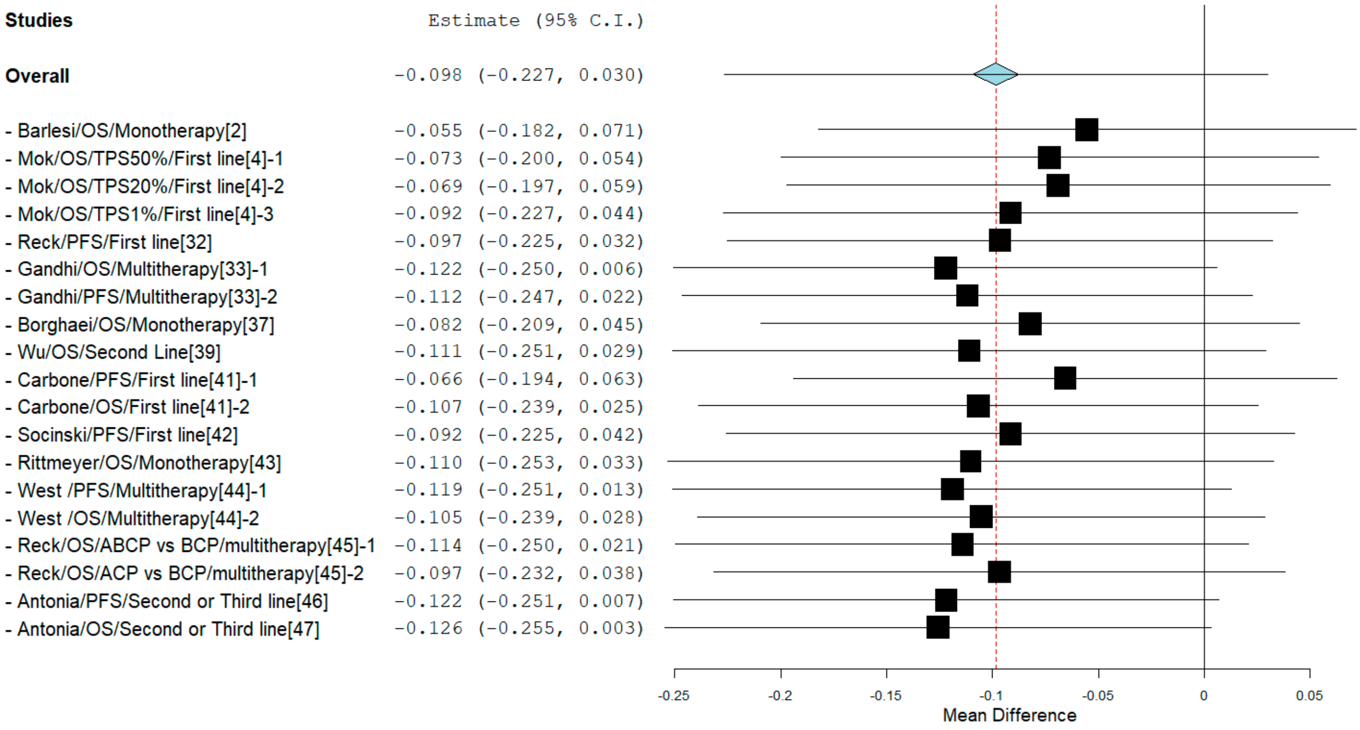

Supplemental Figure s11. Response to treatment by anti-PD-1/PD-L1drugs between smoker and non-smokers using fixed effect leaveoneout model.

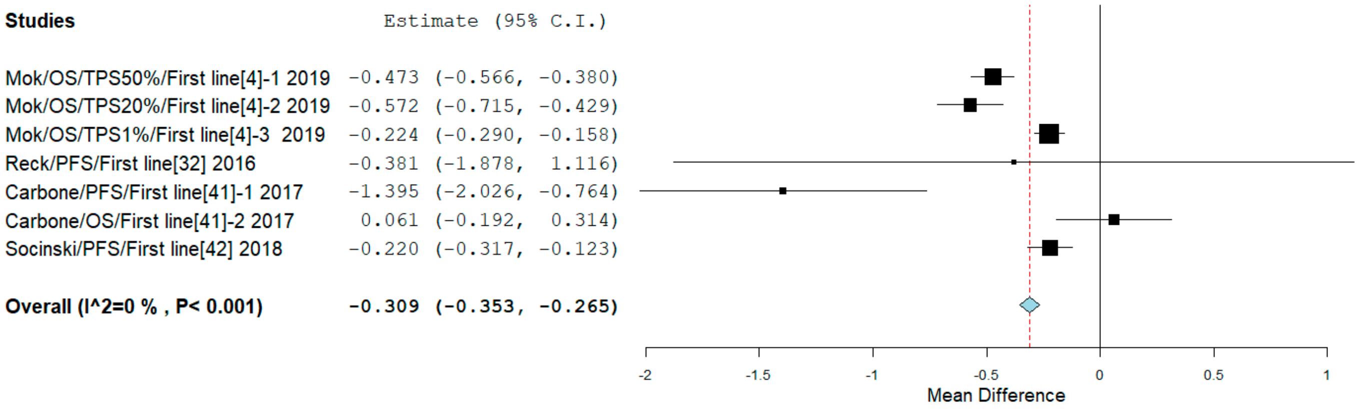

Supplemental Figure s12. Response to treatment between smoker and non-smokers when treated as firs line treatment by anti-PD-1/PD-L1drugs using FE model.

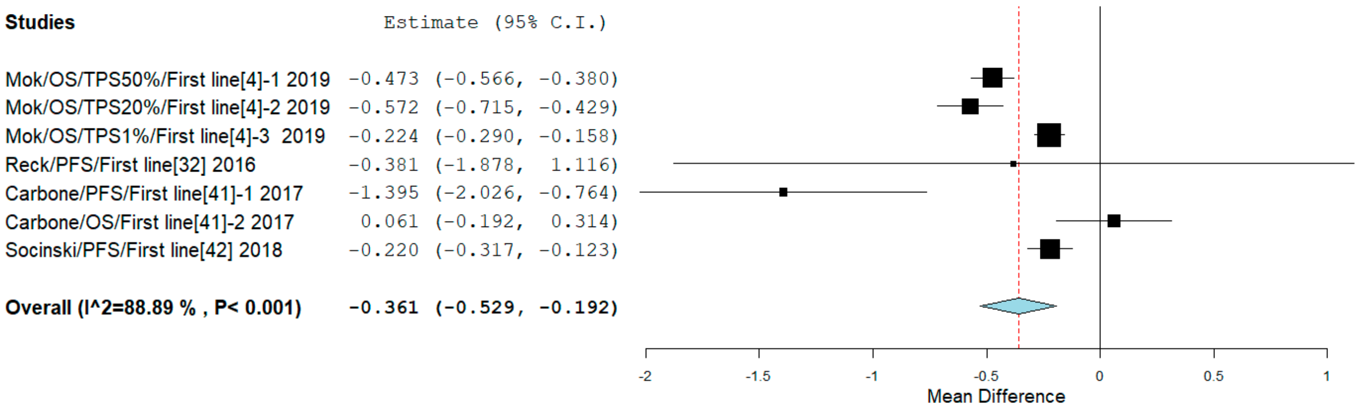

Supplemental Figure s13. Response to treatment between smoker and non-smokers when treated as firs line treatment by anti-PD-1/PD-L1drugs using RE model.

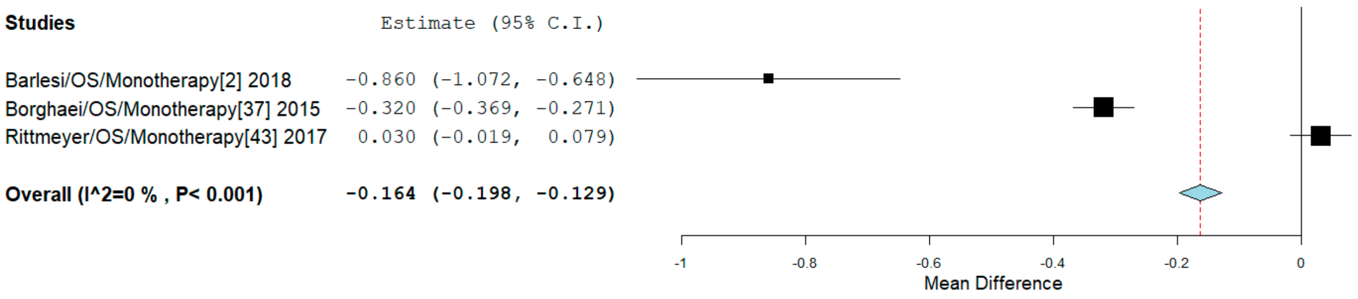

Supplemental Figure s14. Response to treatment between smoker and non-smokers when treated as mon treatment by anti-PD-1/PD-L1drugs using FE model.

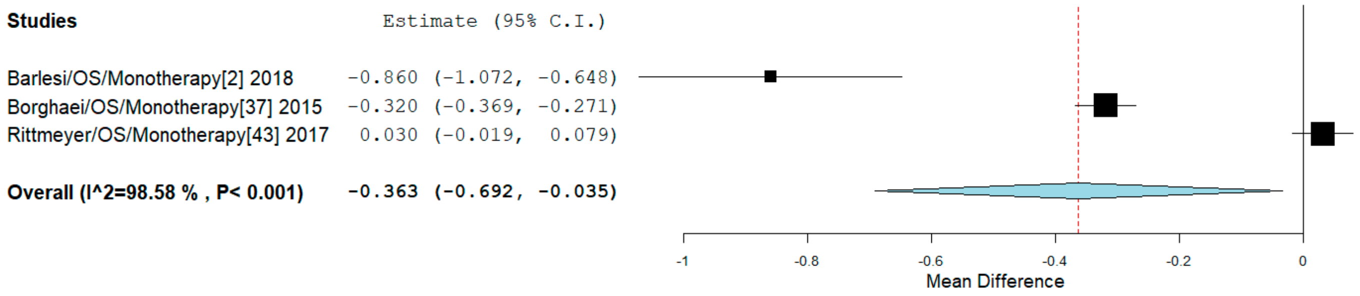

Supplemental Figure s15. Response to treatment between smoker and non-smokers when treated as mono treatment by anti-PD-1/PD-L1drugs using RE model.

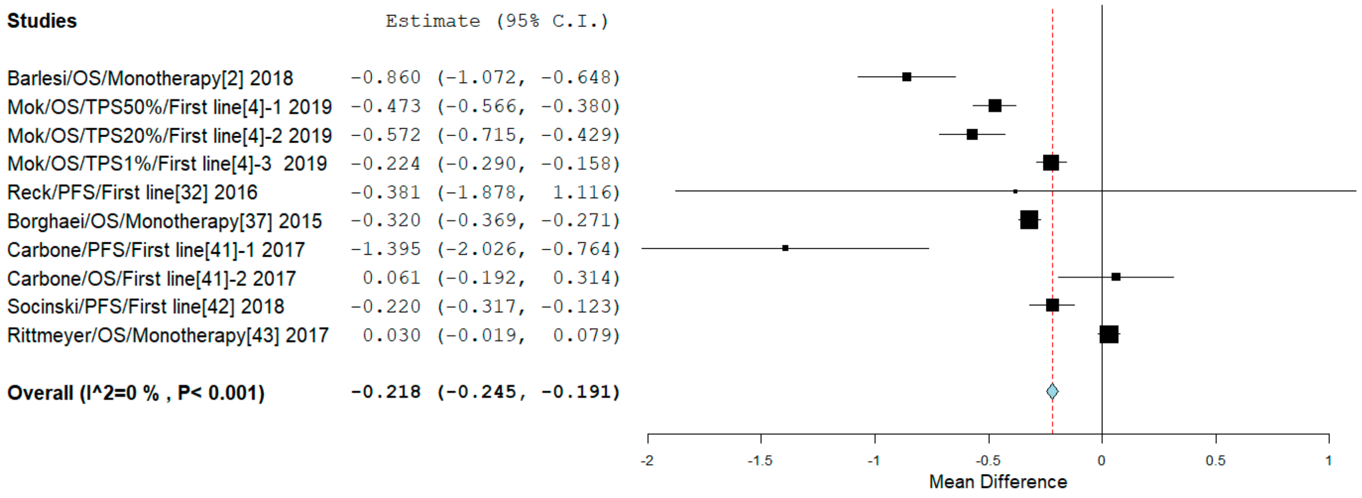

Supplemental Figure s16. Response to treatment between smoker and non-smokers when treated as mono and first line treatment by anti-PD-1/PD-L1drugs using FE model.

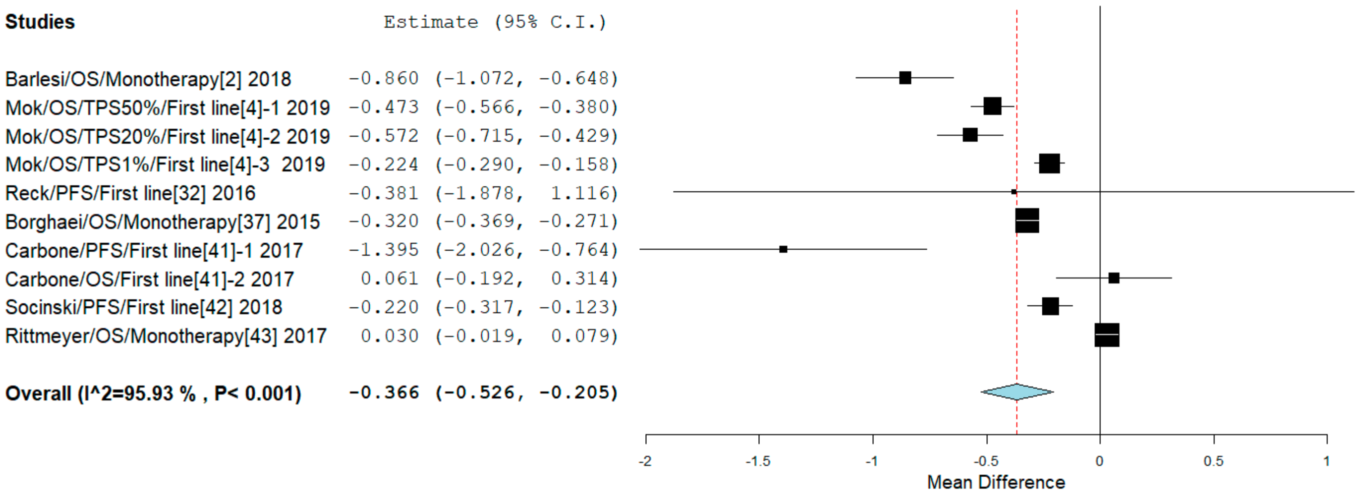

Supplemental Figure s17. **Response to treatment between smoker and non-smokers when treated as second line treatment by anti-PD-1/PD-L1drugs using FE model.**

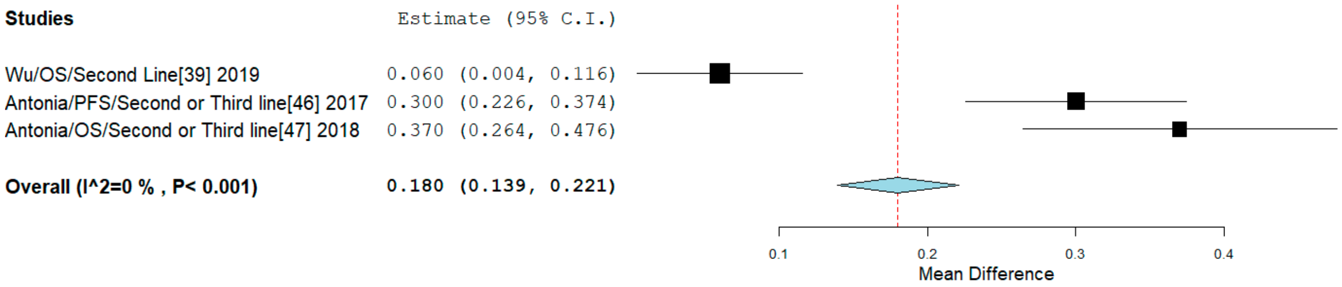

Supplemental Figure s18. **Response to treatment between smoker and non-smokers when treated as second line treatment by anti-PD-1/PD-L1drugs using RE model.**

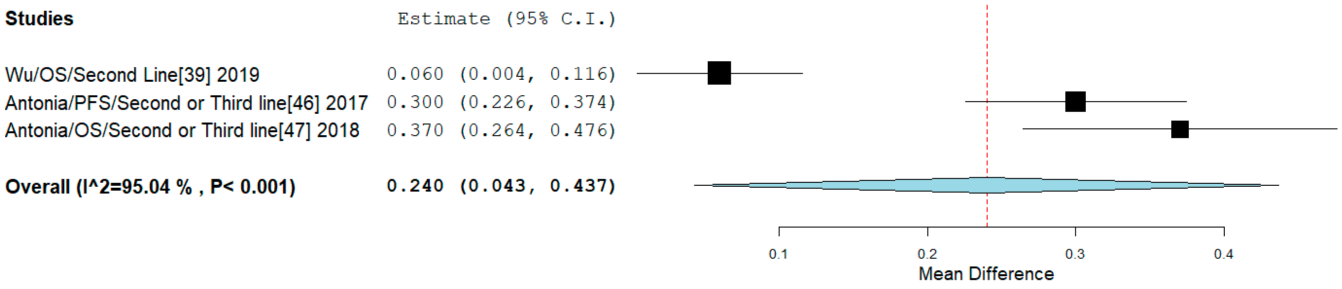

Supplemental Figure s19. Response to treatment between smoker and non-smokers when treated as mono and first line treatment by anti-PD-1/PD-L1drugs using RE model.

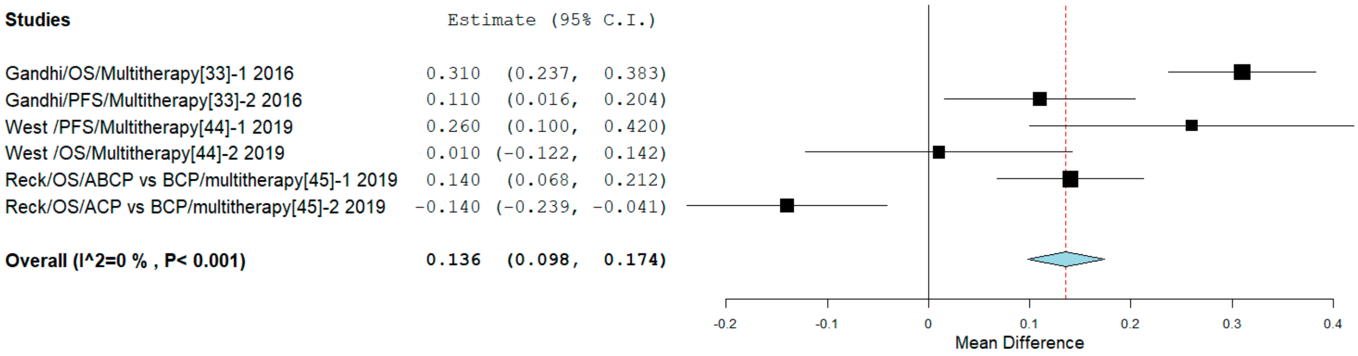

Supplemental Figure s20. Response to treatment between smoker and non-smokers when treated with multiple drugs, anti-PD-1/PD-L1 and other drugs, using FE model.

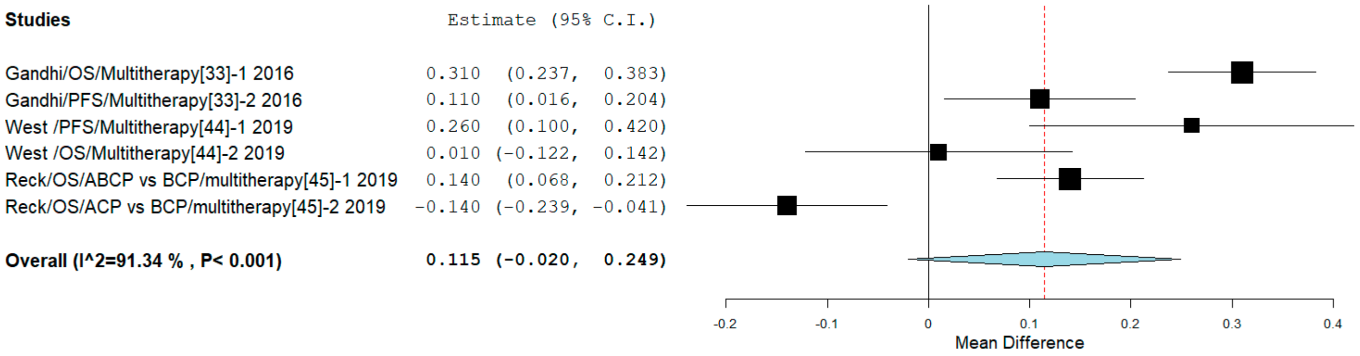

Supplemental Figure s21. Response to treatment between smoker and non-smokers when treated with multiple drugs, anti-PD-1/PD-L1 and other drugs, using RE model.

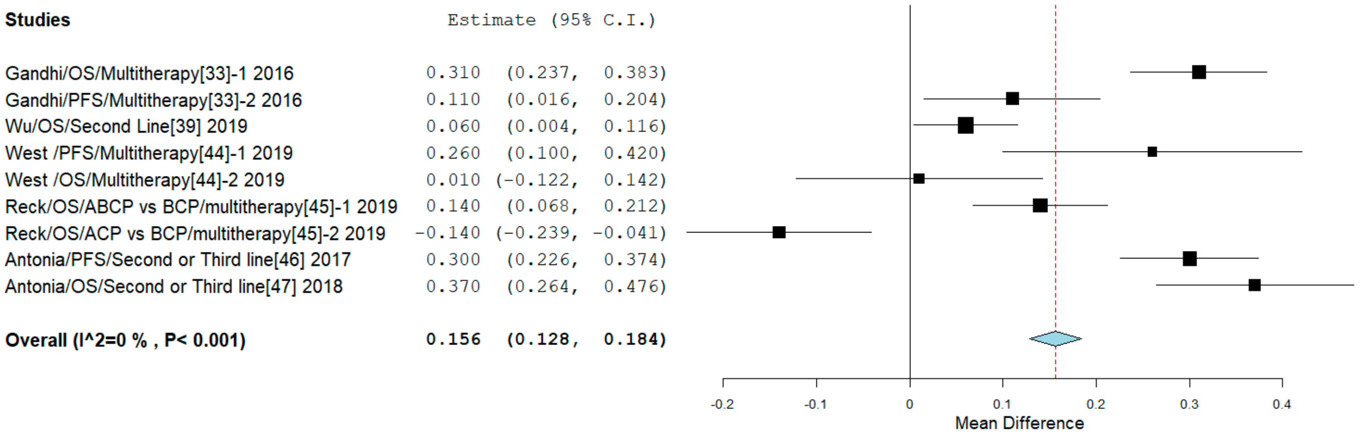

Supplemental Figure s22. Response to treatment between smoker and non-smokers when treated as second line and with multiple drugs, including anti-PD-1/PD-L1 and other drugs, using FE model.

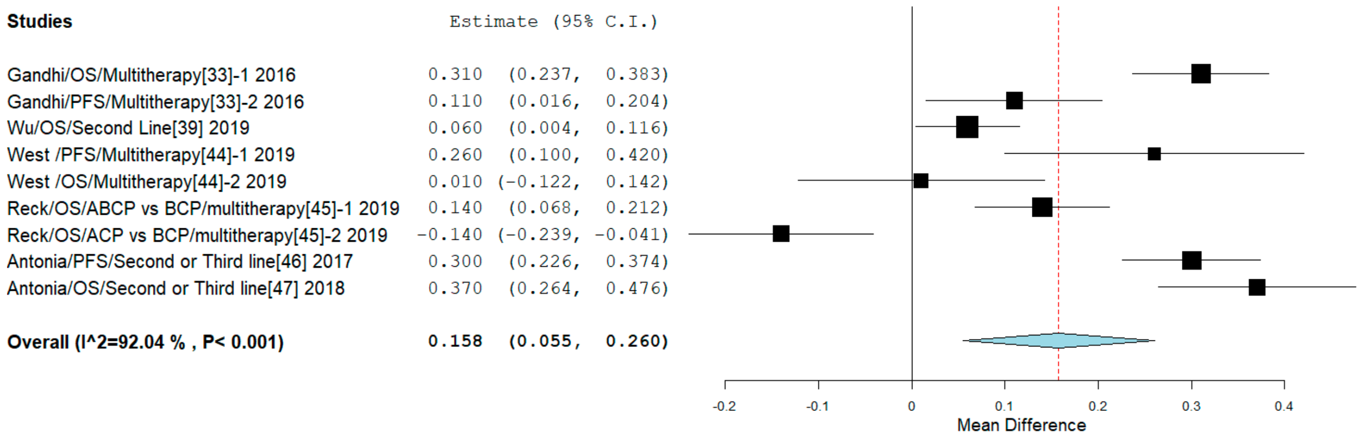

Supplemental Figure s23. Response to treatment between smoker and non-smokers when treated as second lien and with multiple drugs, including anti-PD-1/PD-L1 and other drugs, using RE model.

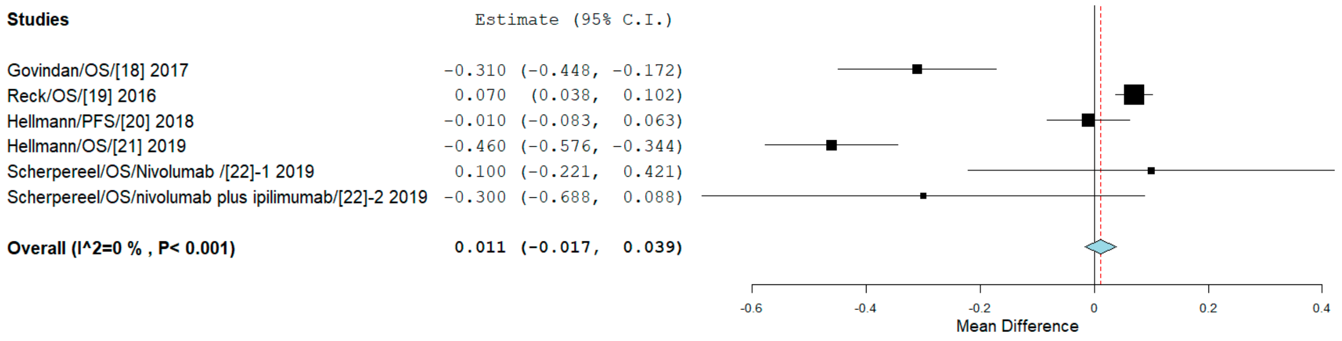

Supplemental Figure s24. Response to treatment between smoker and non-smokers when treated with anti-CTLA-4 drug using FE model.

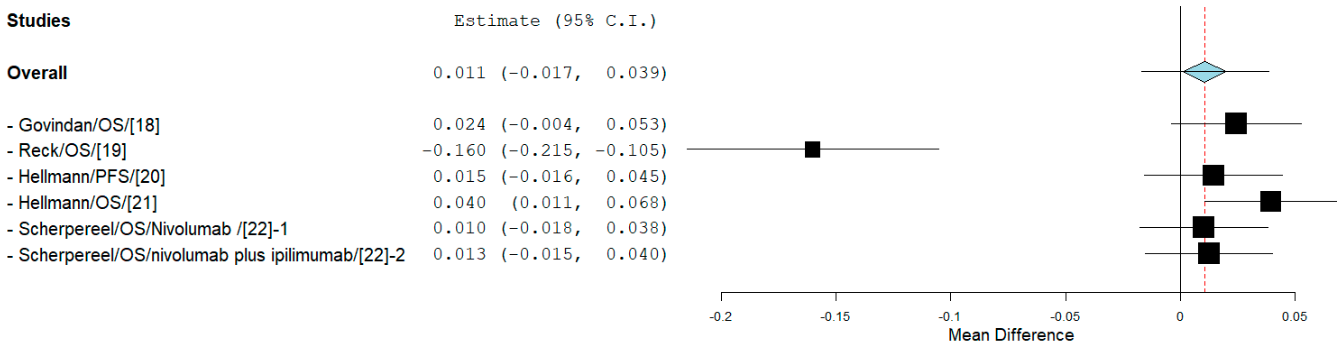

Supplemental Figure s25. Response to treatment between smoker and non-smokers when treated anti-CTLA-4 drug using FE leaveoneout model.

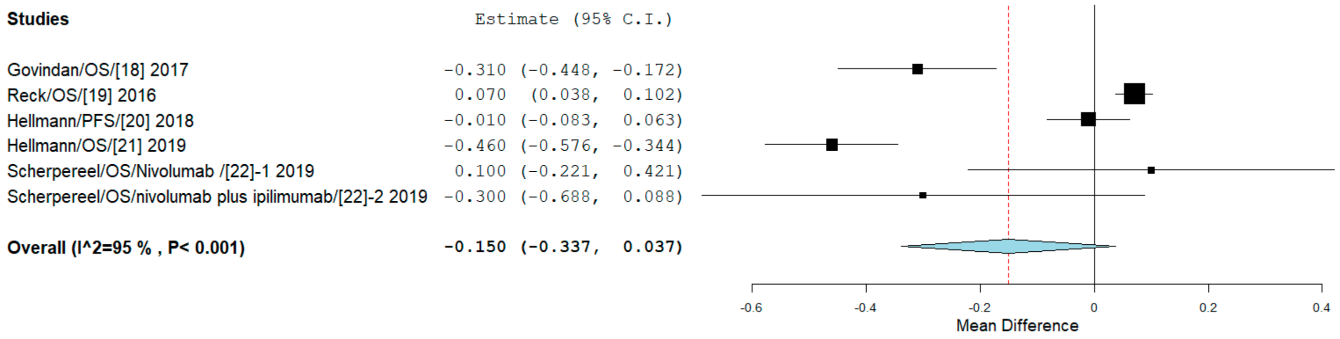

Supplemental Figure s26. Response to treatment between smoker and non-smokers when treated with anti-CTLA-4 drug using RE model.

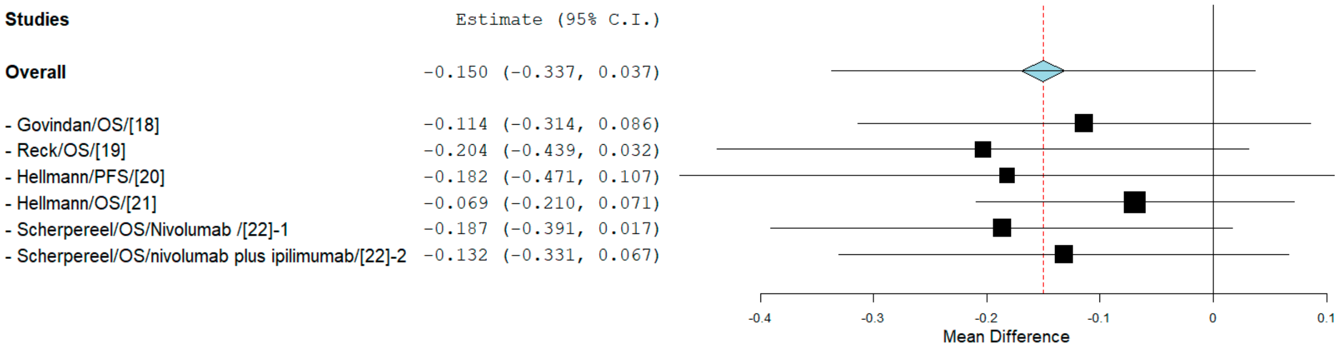

Supplemental Figure s27. Response to treatment between smoker and non-smokers when treated using FE leaveoneout model.

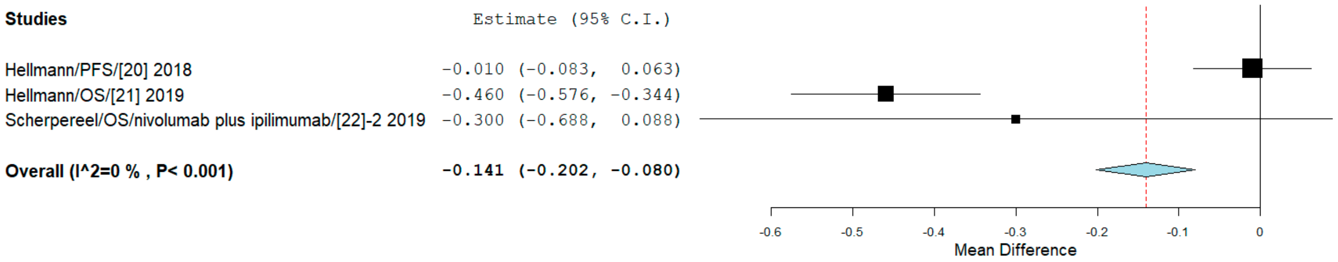

Supplemental Figure s28. Response to treatment between smoker and non-smokers when treated with Nivolumab plus ipilimumab using FE model.

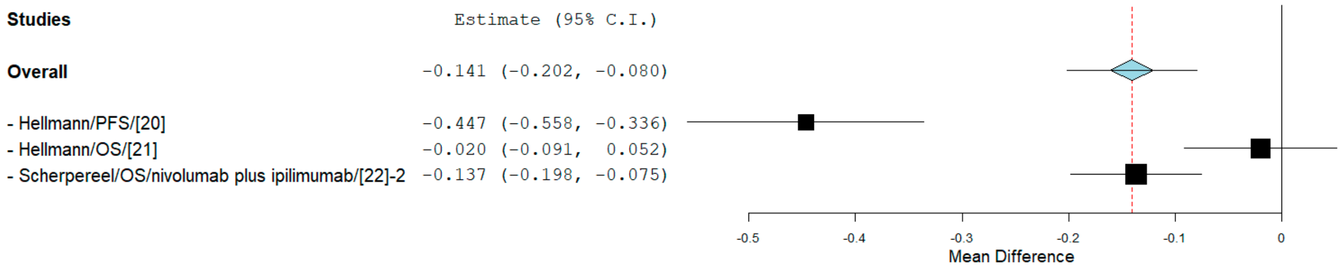

Supplemental Figure s29. Response to treatment between smoker and non-smokers when treated with Nivolumab plus ipilimumab using FE leaveoneout model.

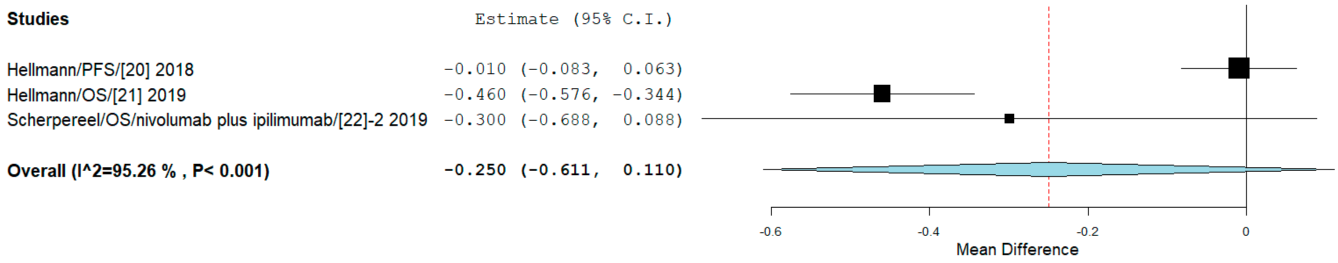

Supplemental Figure s30. Response to treatment between smoker and non-smokers when treated with Nivolumab plus ipilimumab using RE model.

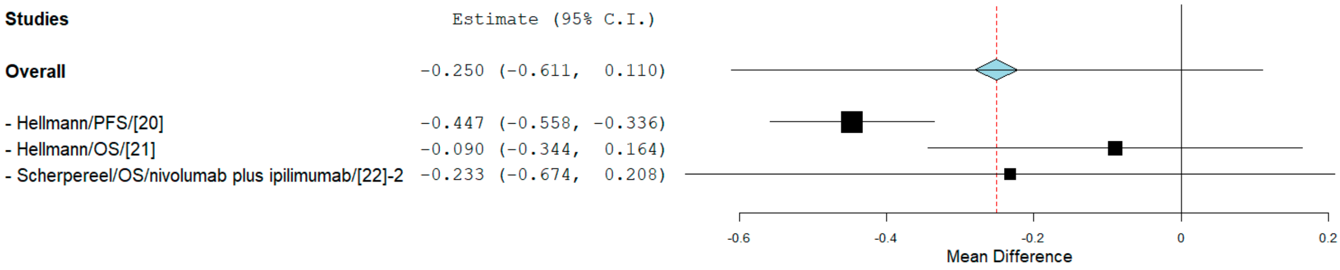

Supplemental Figure s31. Response to treatment between smoker and non-smokers when treated with Nivolumab plus ipilimumab using RE leaveoneout model.

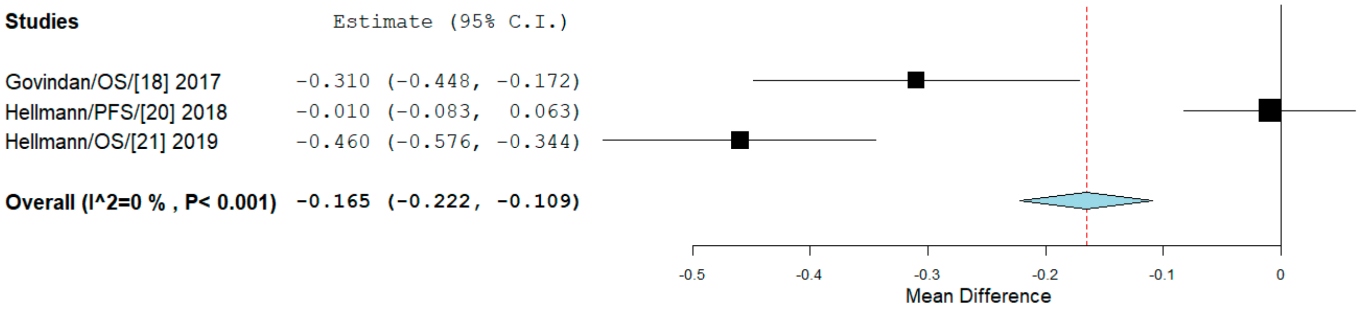

Supplemental Figure s32. Response to treatment between smoker and non-smokers when treated with anti-CTLA-4 drug using FE model in NSCLC patients.

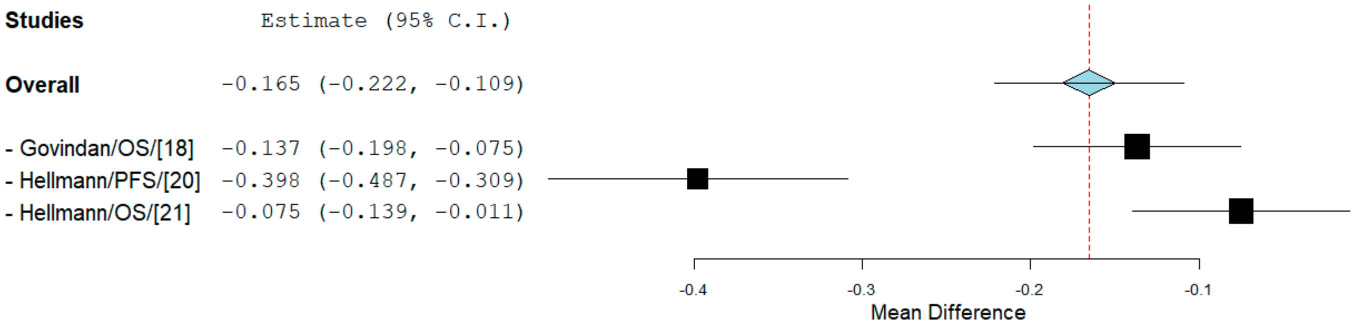

Supplemental Figure s33. Response to treatment between smoker and non-smokers when treated with anti-CTLA-4 drug using RE leaveoneout model in NSCLC patients.

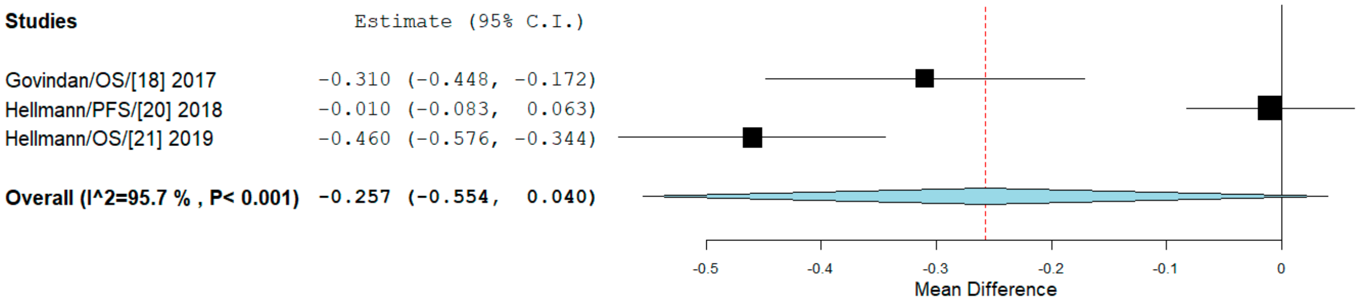

Supplemental Figure s34. Response to treatment between smoker and non-smokers when treated with anti-CTLA-4 drug using RE leaveoneout model in NSCLC patients.

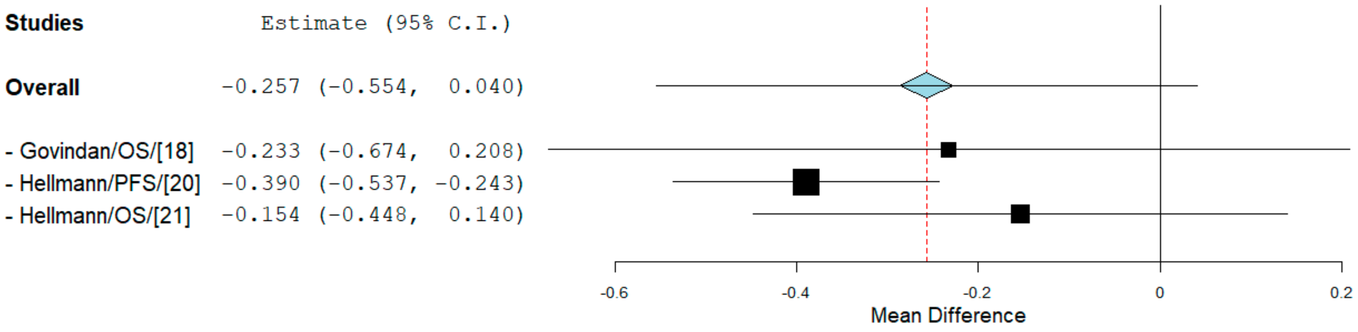

Supplemental Figure s35. Response to treatment between smoker and non-smokers when treated anti-CTLA-4 drug using RE leaveoneout model in NSCLC patients.

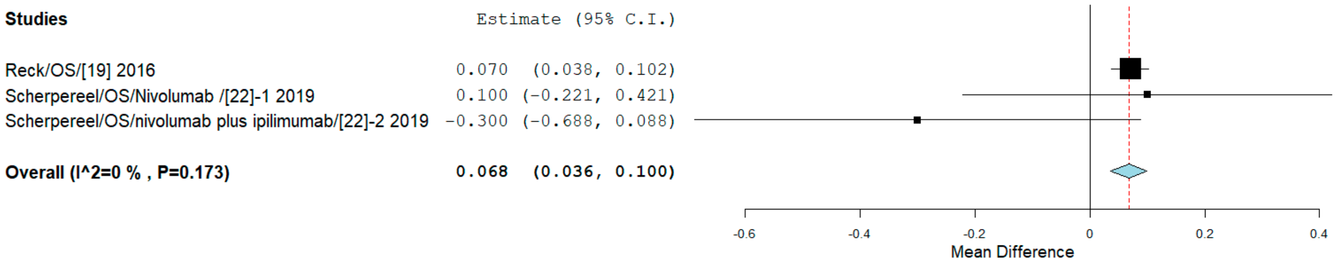

Supplemental Figure s36. Response to treatment between smoker and non-smokers when treated with anti-CTLA-4 drug using FE model in non-NSCLC patients.

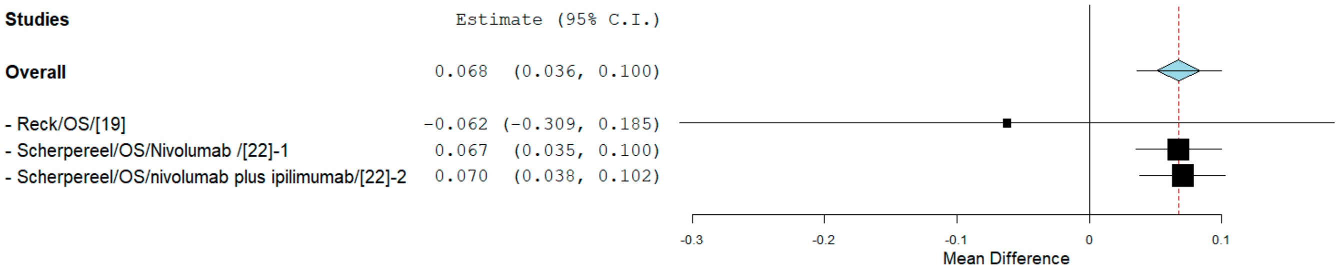

Supplemental Figure s37. Response to treatment between smoker and non-smokers when treated with anti-CTLA-4 drug using FE leaveoneout model in non-NSCLC patients.

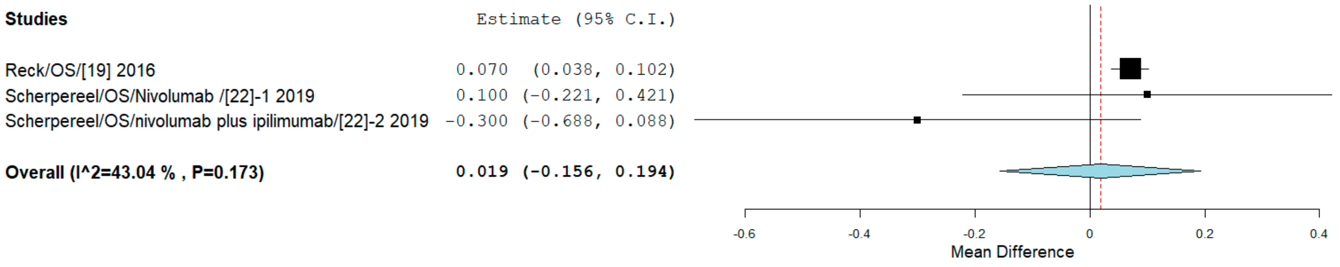

Supplemental Figure s38. Response to treatment between smoker and non-smokers when treated with anti-CTLA-4 drug using RE model in non-NSCLC patients.

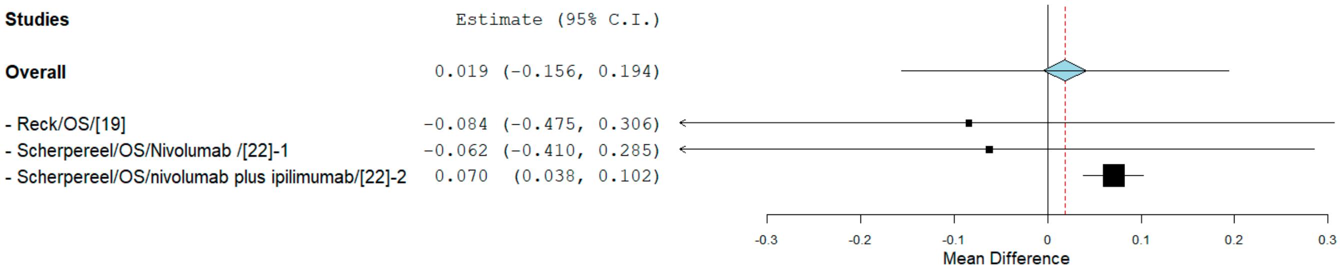

Supplemental Figure s39. Response to treatment between smoker and non-smokers when treated with anti-CTLA-4 drug using RE leaveoneout model in non-NSCLC patients.

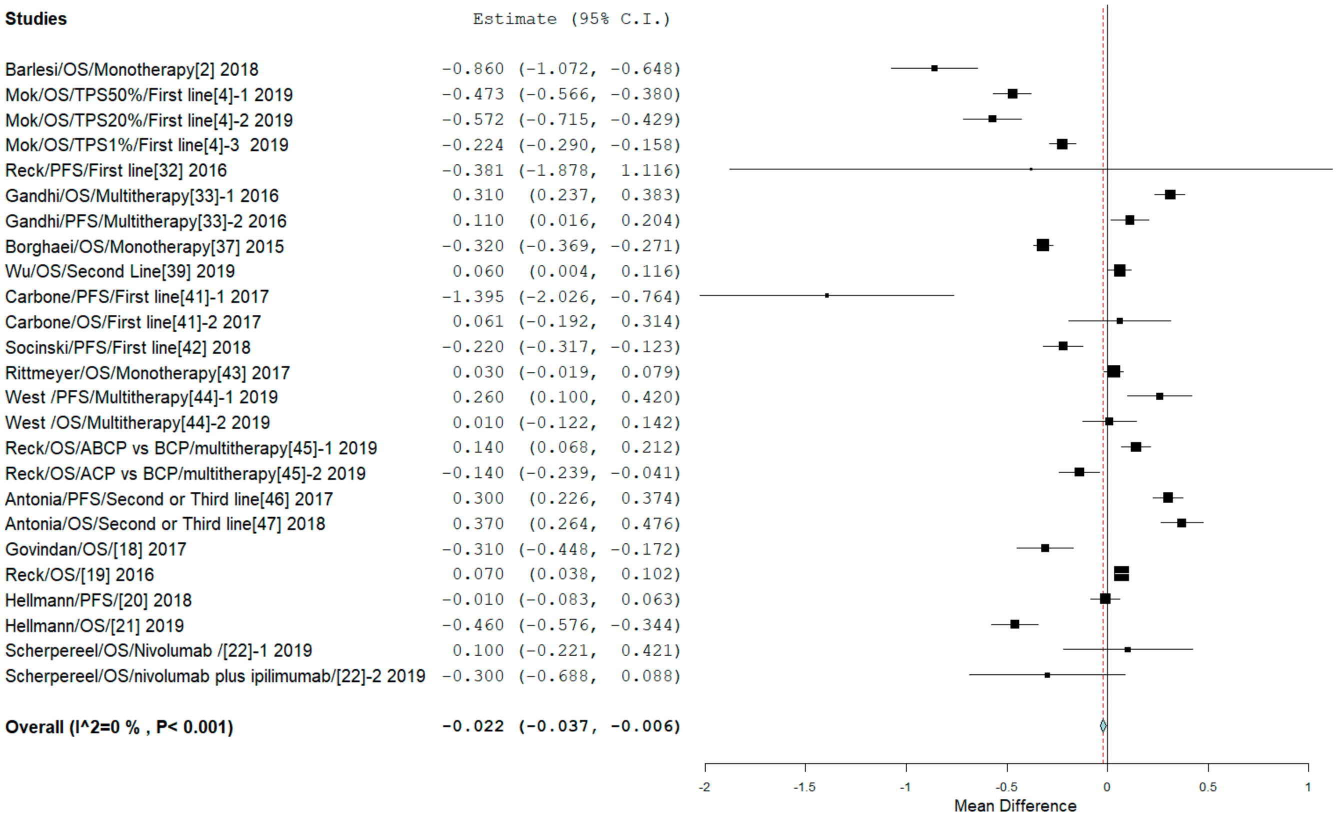

Supplemental Figure s40. Response to treatment between smoker and non-smokers when treated with anti-PD-1 and anti-CTLA-4 drugs using FE model in cancer patients.

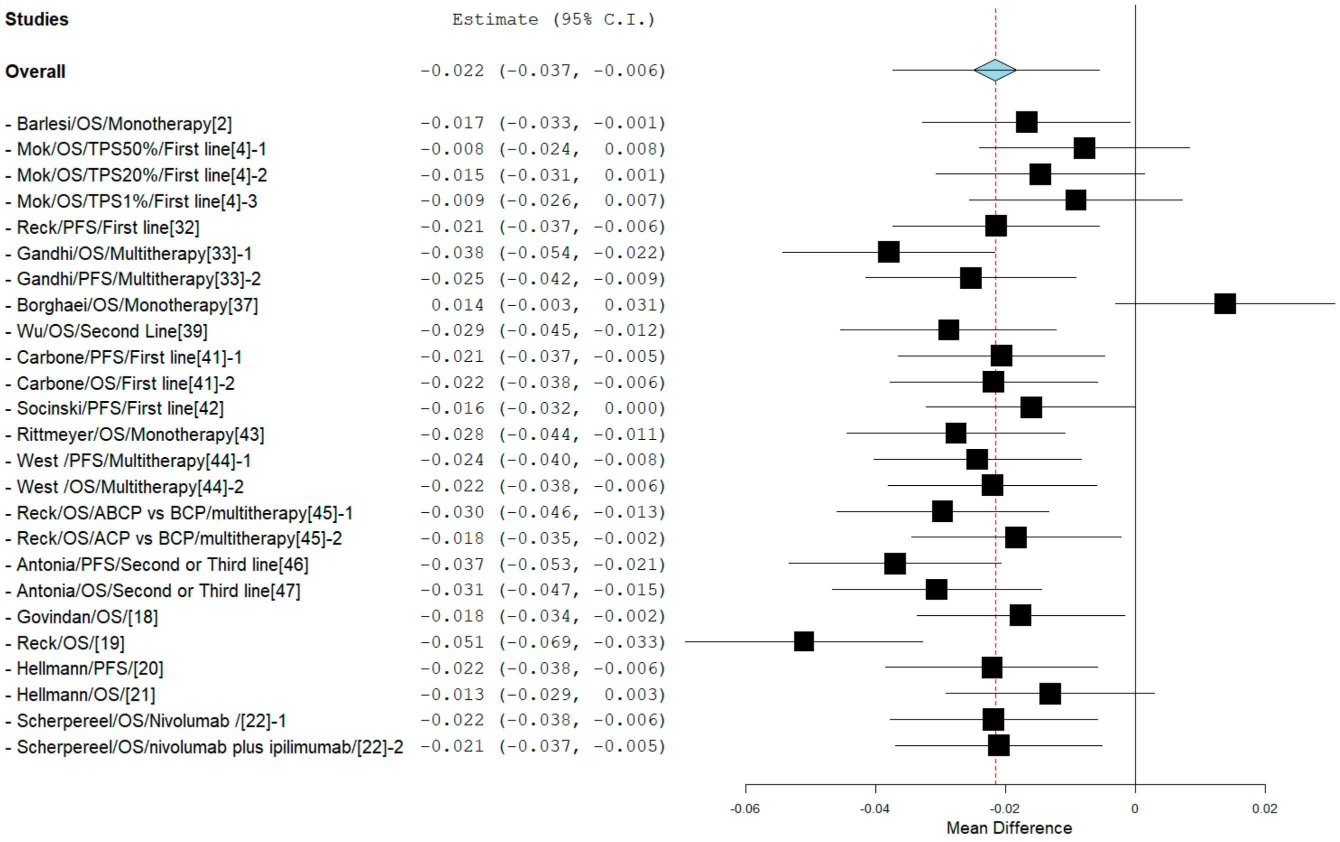

Supplemental Figure s41. Response to treatment between smoker and non-smokers when treated with anti-PD-1 and anti-CTLA-4 drugs using FE leaveoneout model in cancer patients.

Studies	Estimate (95% C.I.)
Barlesi/OS/Monotherapy[2] 2018	-0.860 (-1.072, -0.648)
Mok/OS/TPS50%/First line[4]-1 2019	-0.473 (-0.566, -0.380)
Mok/OS/TPS20%/First line[4]-2 2019	-0.572 (-0.715, -0.429)
Mok/OS/TPS1%/First line[4]-3 2019	-0.224 (-0.290, -0.158)
Reck/PFS/First line[32] 2016	-0.381 (-1.878, 1.116)
Gandhi/OS/Multitherapy[33]-1 2016	0.310 (0.237, 0.383)
Gandhi/PFS/Multitherapy[33]-2 2016	0.110 (0.016, 0.204)
Borghaei/OS/Monotherapy[37] 2015	-0.320 (-0.369, -0.271)
Wu/OS/Second Line[39] 2019	0.060 (0.004, 0.116)
Carbone/PFS/First line[41]-1 2017	-1.395 (-2.026, -0.764)
Carbone/OS/First line[41]-2 2017	0.061 (-0.192, 0.314)
Socinski/PFS/First line[42] 2018	-0.220 (-0.317, -0.123)
Rittmeyer/OS/Monotherapy[43] 2017	0.030 (-0.019, 0.079)
West /PFS/Multitherapy[44]-1 2019	0.260 (0.100, 0.420)
West /OS/Multitherapy[44]-2 2019	0.010 (-0.122, 0.142)
Reck/OS/ABCP vs BCP/multitherapy[45]-1 2019	0.140 (0.068, 0.212)
Reck/OS/ACP vs BCP/multitherapy[45]-2 2019	-0.140 (-0.239, -0.041)
Antonia/PFS/Second or Third line[46] 2017	0.300 (0.226, 0.374)
Antonia/OS/Second or Third line[47] 2018	0.370 (0.264, 0.476)
Govindan/OS/[18] 2017	-0.310 (-0.448, -0.172)
Reck/OS/[19] 2016	0.070 (0.038, 0.102)
Hellmann/PFS/[20] 2018	-0.010 (-0.083, 0.063)
Hellmann/OS/[21] 2019	-0.460 (-0.576, -0.344)
Scherpereel/OS/Nivolumab /[22]-1 2019	0.100 (-0.221, 0.421)
Scherpereel/OS/nivolumab plus ipilimumab/[22]-2 2019	-0.300 (-0.688, 0.088)
**Overall (I^2=96.95 % , P< 0.001)**	**-0.106 (-0.207, -0.006)**

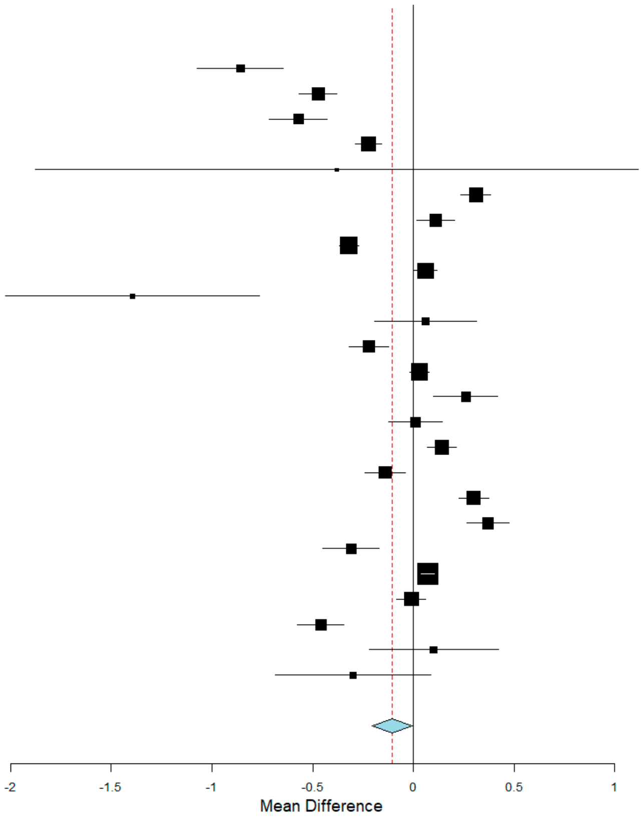

Supplemental Figure s42. Response to treatment between smoker and non-smokers when treated with anti-PD-1 and anti-CTLA-4 drugs using RE model in cancer patients.

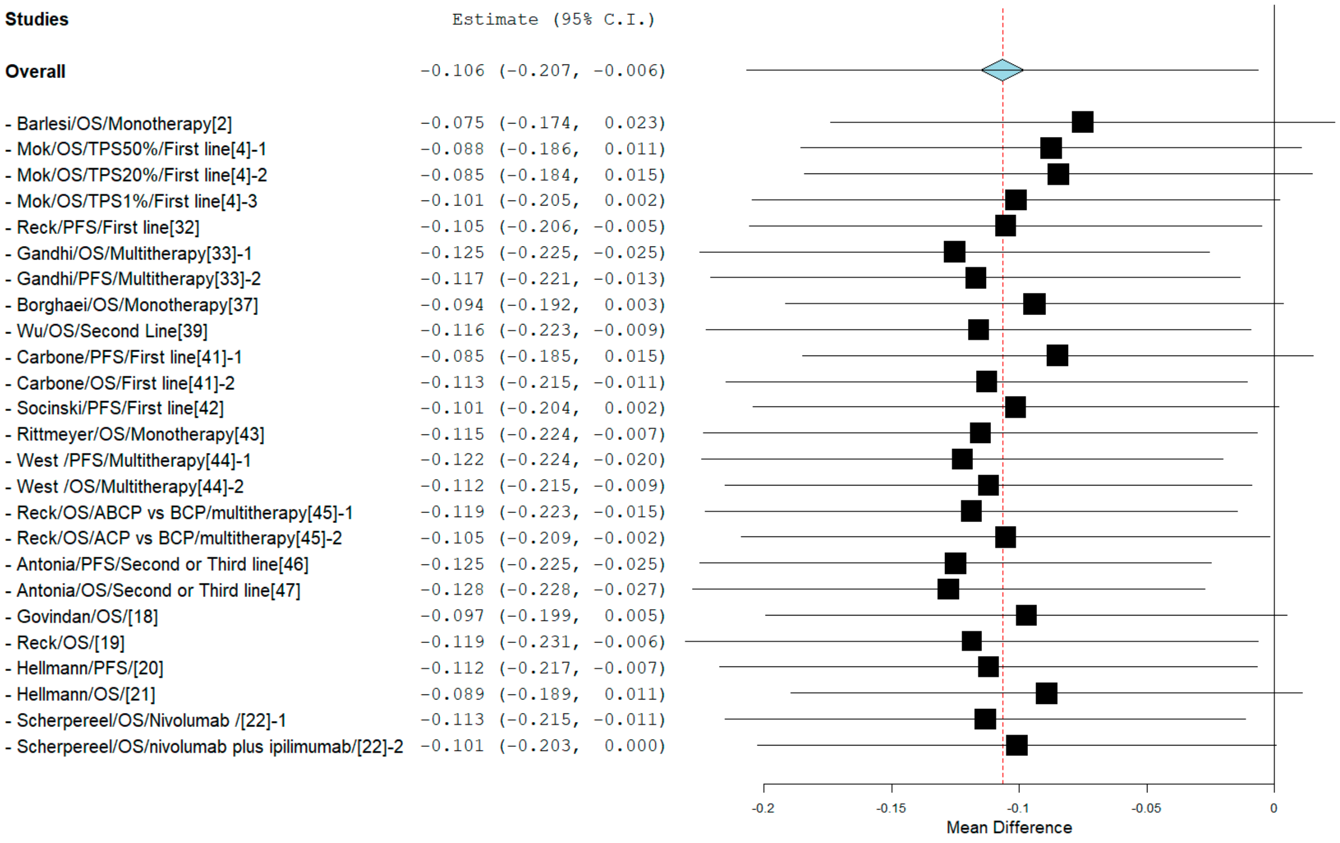

Supplemental Figure s43. Response to treatment between smoker and non-smokers when treated with anti-PD-1 and anti-CTLA-4 drugs using RE leaveoneout model in cancer patients.

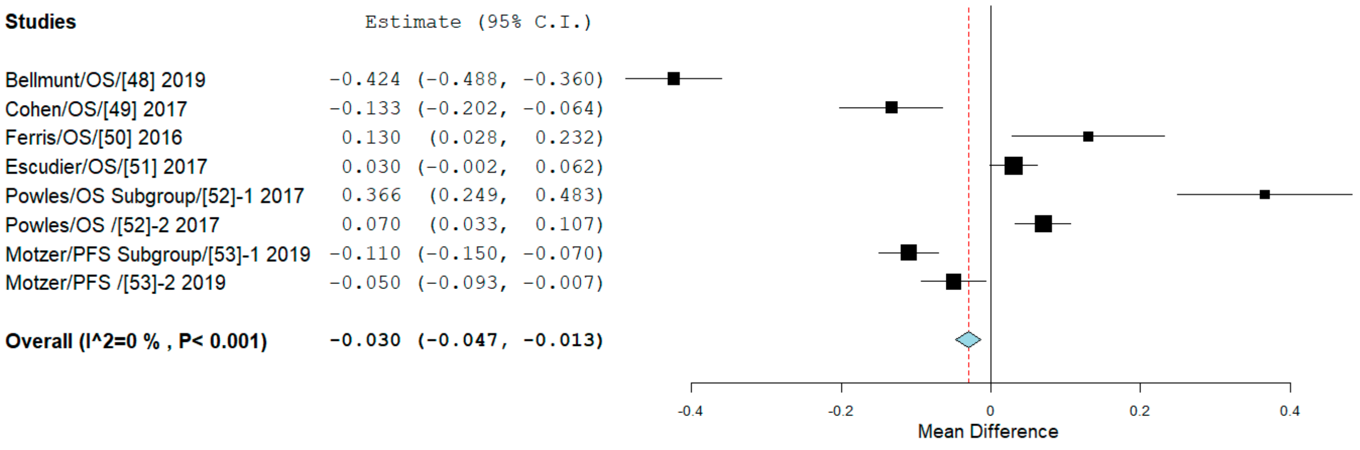

Supplemental Figure s44. Response to treatment between smoker and non-smokers when treated with anti-PD- using FE l model in non-NSCLC patients.

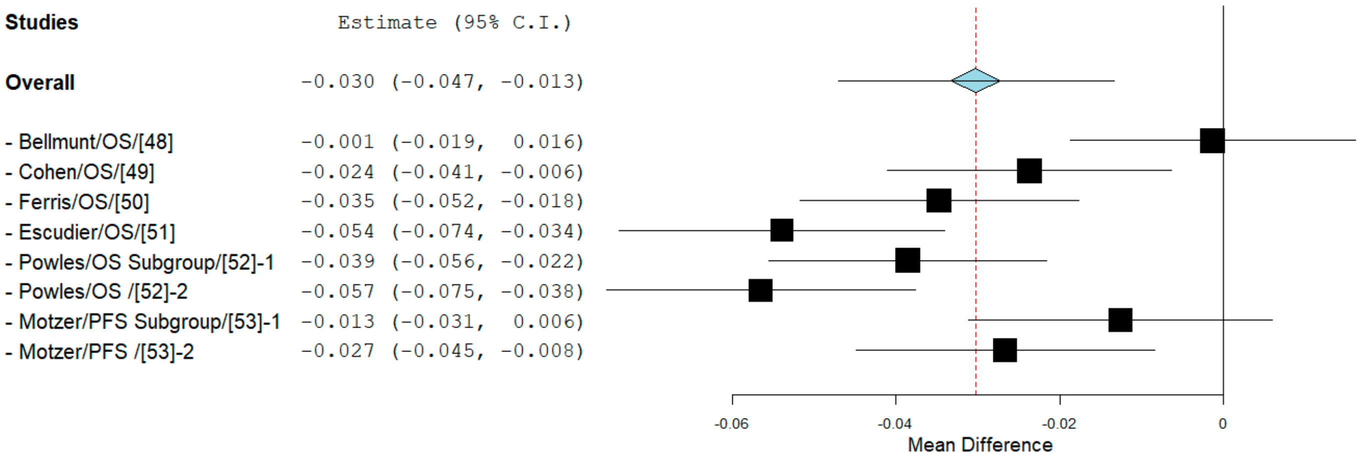

Supplemental Figure s45. Response to treatment between smoker and non-smokers when treated with anti-PD-1 drugs

using FE leaveoneout model in non-NSCLC patients.

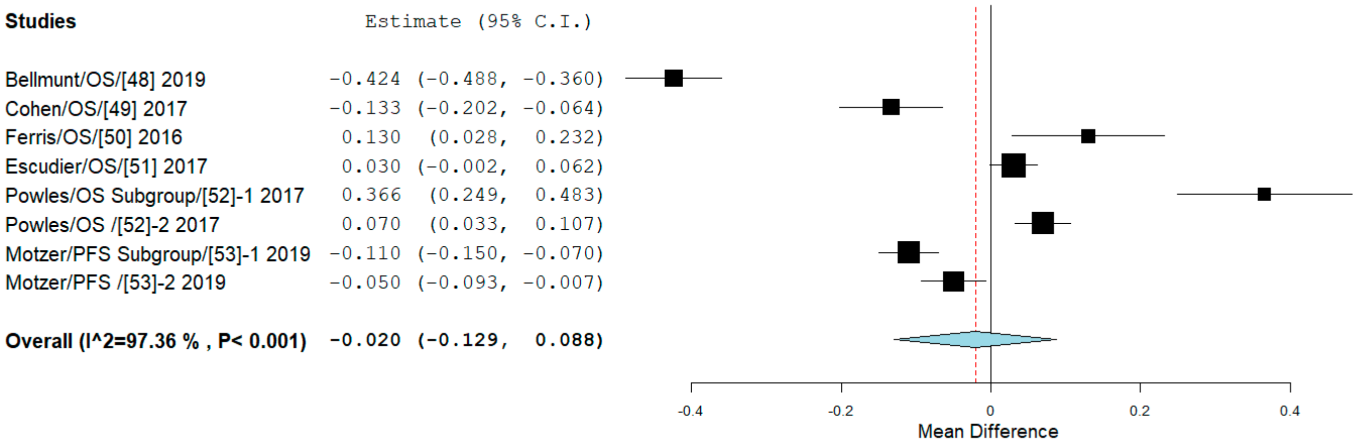

Supplemental Figure s46. Response to treatment between smoker and non-smokers when treated with anti-PD-1 drugs using RE model in non-NSCLC patients.

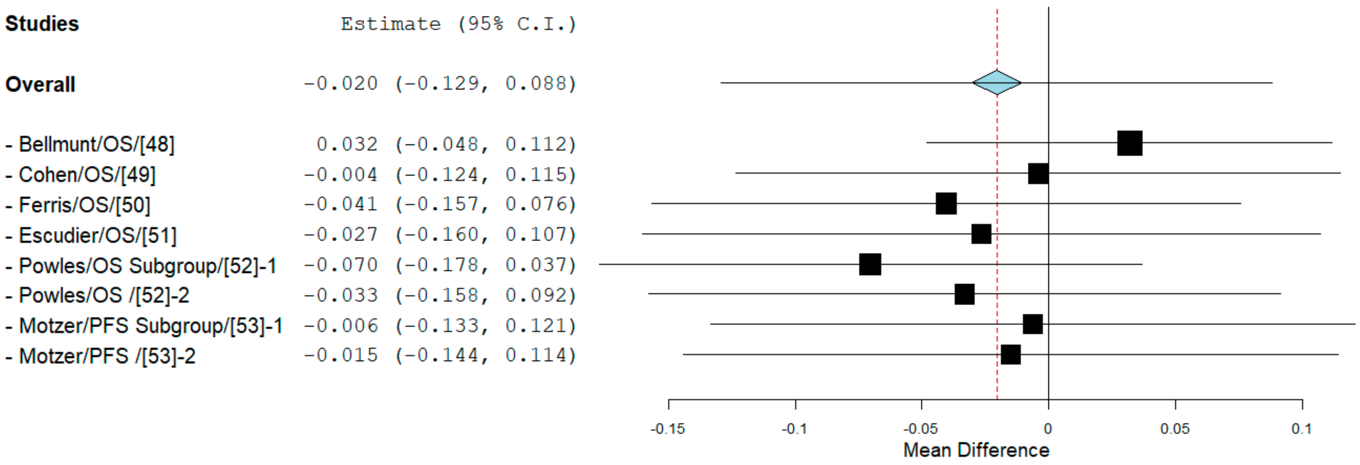

Supplemental Figure s47. **Response to treatment between smoker and non-smokers when treated treated with anti-PD-1 drugs using RE leaveoneout model in non-NSCLC patients.**

Studies	Estimate (95% C.I.)
Barlesi/OS/Monotherapy[2] 2018	-0.860 (-1.072, -0.648)
Mok/OS/TPS50%/First line[4]-1 2019	-0.473 (-0.566, -0.380)
Mok/OS/TPS20%/First line[4]-2 2019	-0.572 (-0.715, -0.429)
Mok/OS/TPS1%/First line[4]-3 2019	-0.224 (-0.290, -0.158)
Reck/PFS/First line[32] 2016	-0.381 (-1.878, 1.116)
Gandhi/OS/Multitherapy[33]-1 2016	0.310 (0.237, 0.383)
Gandhi/PFS/Multitherapy[33]-2 2016	0.110 (0.016, 0.204)
Borghaei/OS/Monotherapy[37] 2015	-0.320 (-0.369, -0.271)
Wu/OS/Second Line[39] 2019	0.060 (0.004, 0.116)
Carbone/PFS/First line[41]-1 2017	-1.395 (-2.026, -0.764)
Carbone/OS/First line[41]-2 2017	0.061 (-0.192, 0.314)
Socinski/PFS/First line[42] 2018	-0.220 (-0.317, -0.123)
Rittmeyer/OS/Monotherapy[43] 2017	0.030 (-0.019, 0.079)
West /PFS/Multitherapy[44]-1 2019	0.260 (0.100, 0.420)
West /OS/Multitherapy[44]-2 2019	0.010 (-0.122, 0.142)
Reck/OS/ABCP vs BCP/multitherapy[45]-1 2019	0.140 (0.068, 0.212)
Reck/OS/ACP vs BCP/multitherapy[45]-2 2019	-0.140 (-0.239, -0.041)
Antonia/PFS/Second or Third line[46] 2017	0.300 (0.226, 0.374)
Antonia/OS/Second or Third line[47] 2018	0.370 (0.264, 0.476)
Govindan/OS/[18] 2017	-0.310 (-0.448, -0.172)
Reck/OS/[19] 2016	0.070 (0.038, 0.102)
Hellmann/PFS/[20] 2018	-0.010 (-0.083, 0.063)
Hellmann/OS/[21] 2019	-0.460 (-0.576, -0.344)
Scherpereel/OS/Nivolumab /[22]-1 2019	0.100 (-0.221, 0.421)
Scherpereel/OS/nivolumab plus ipilimumab/[22]-2 2019	-0.300 (-0.688, 0.088)
Butts/OS concurrent chemoradiotherapy/[25]-1 2013	-0.760 (-1.193, -0.327)
Butts/OS sequential chemoradiotherapy/[25]-2 2013	-3.830 (-8.243, 0.583)
Katakami/OS/[26] 2017	-0.100 (-0.936, 0.736)
**Overall (I^2=0 % , P< 0.001)**	**-0.023 (-0.039, -0.007)**

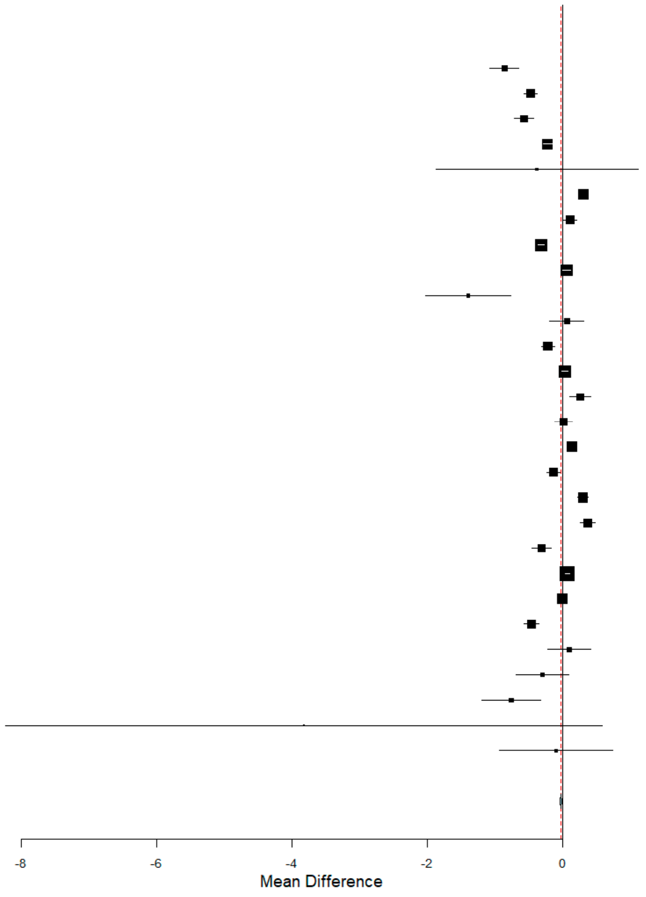

Supplemental Figure s48. Response to treatment between smoker and non-smokers when treated with anti-PD-1, anti-CTLA-4, and anti-MUC1 drugs using FE model in cancer patients.

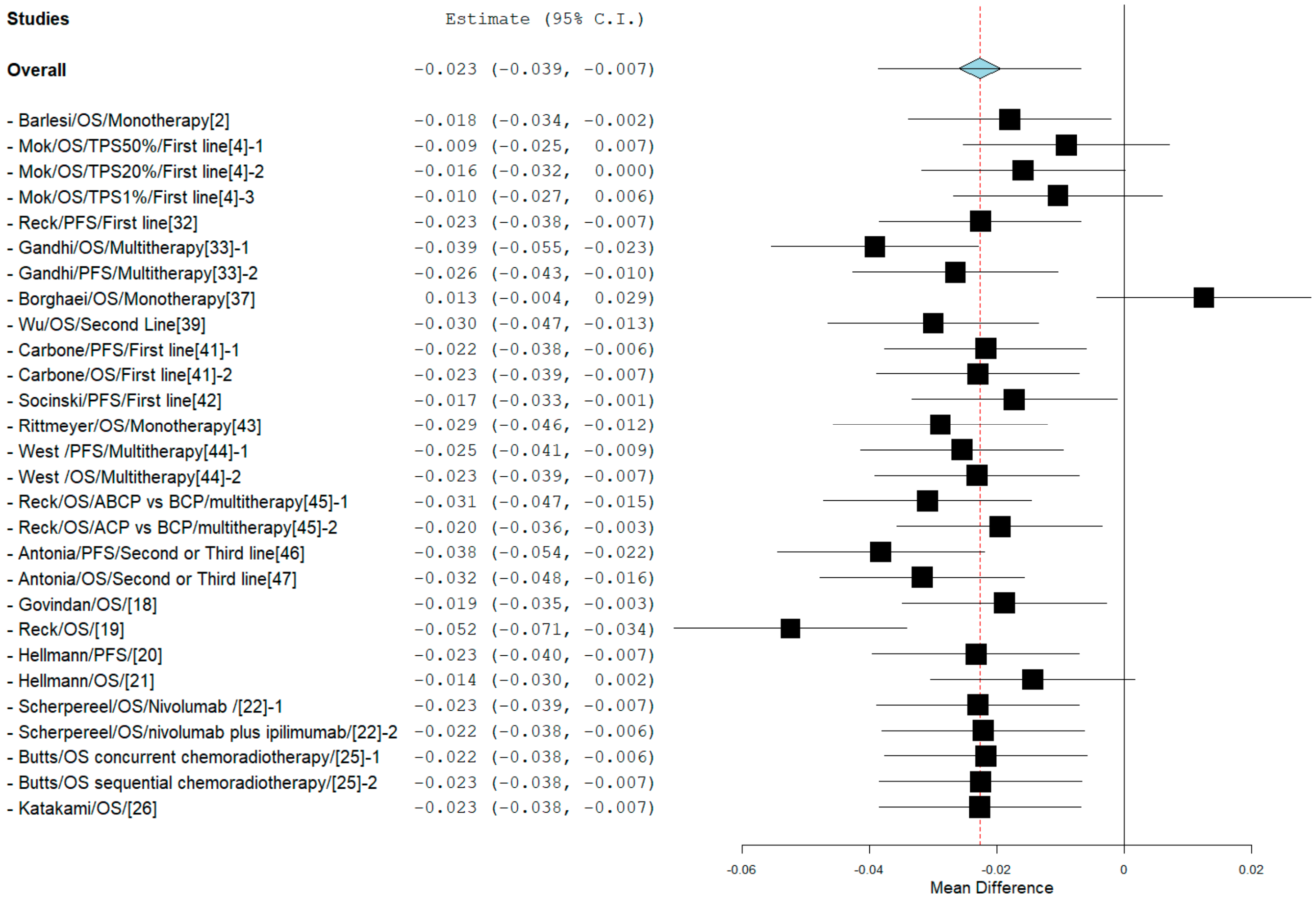

Supplemental Figure s49. Response to treatment between smoker and non-smokers when treated with anti-PD-1, anti-CTLA-4, and anti-MUC1 drugs using FE leaveoneout model in cancer patients.

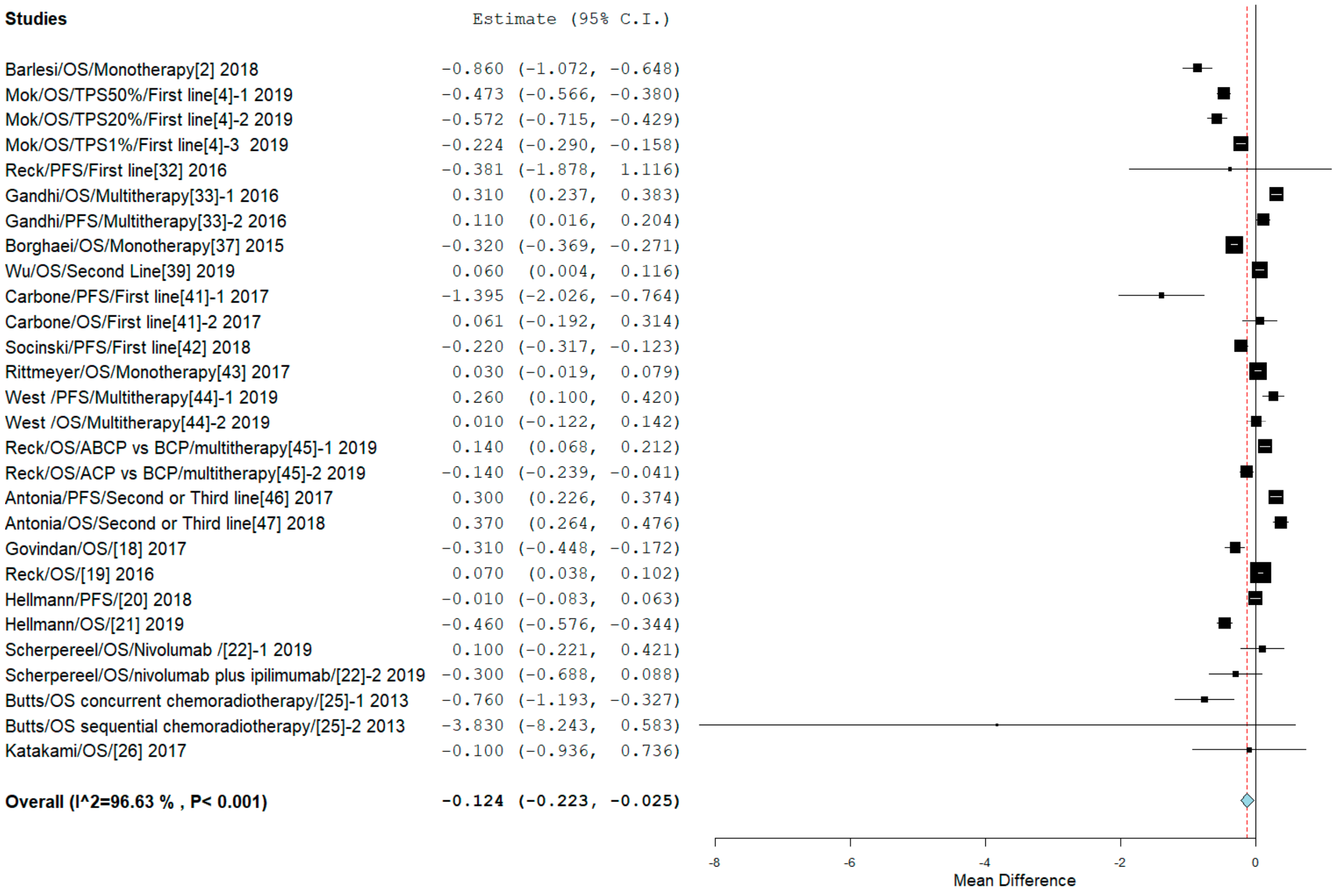

Supplemental Figure s50. Response to treatment between smoker and non-smokers when treated with anti-PD-1 and anti-CTLA-4 drugs using RE model in cancer patients.

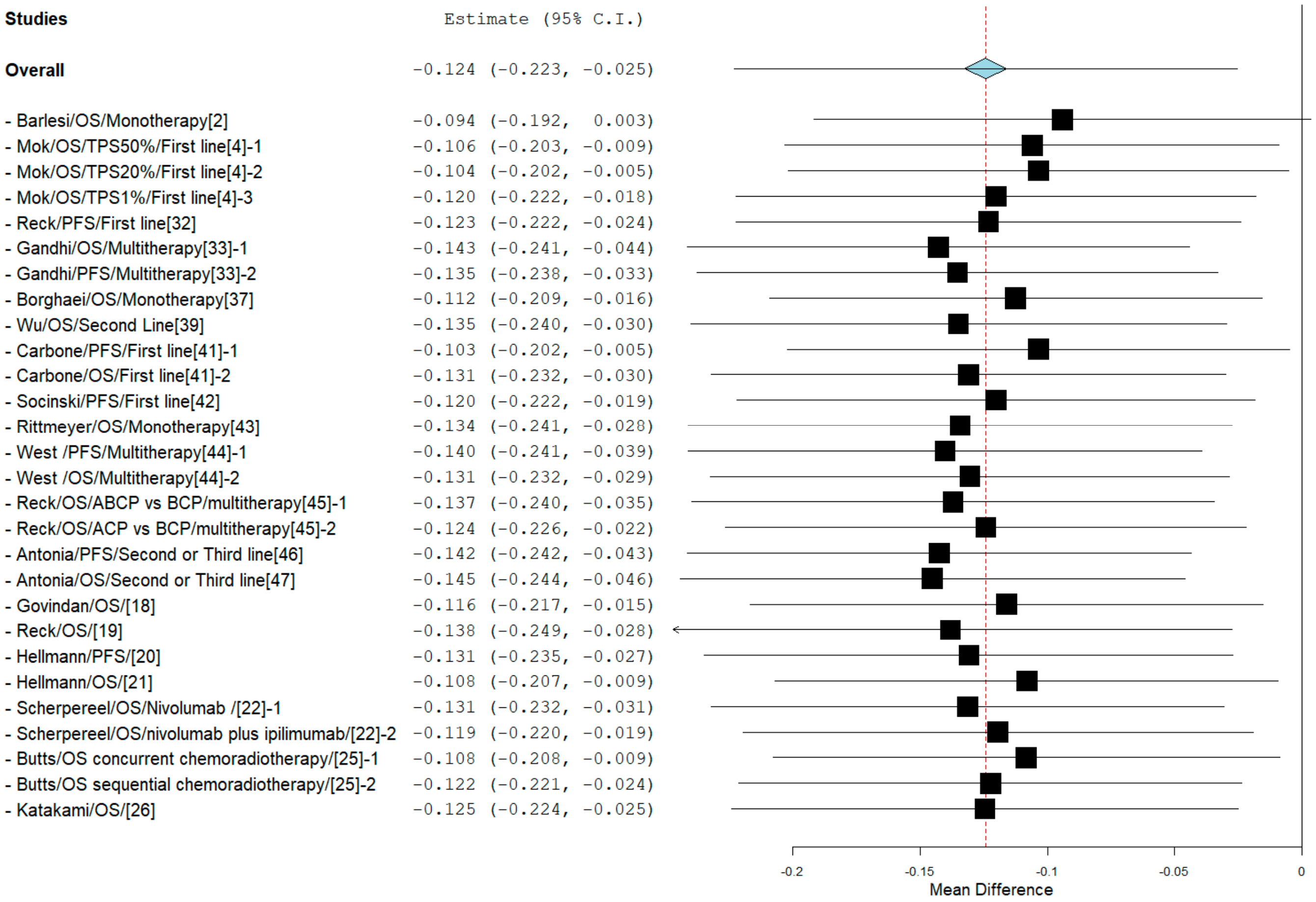

Supplemental Figure s51. **Response to treatment between smoker and non-smokers when treated with anti-PD-1, anti-CTLA-4, and anti-MUC1 drugs using RE leaveoneout model in cancer patients.**

Studies	Estimate (95% C.I.)
Barlesi/OS/Monotherapy[2] 2018	-0.860 (-1.072, -0.648)
Mok/OS/TPS50%/First line[4]-1 2019	-0.473 (-0.566, -0.380)
Mok/OS/TPS20%/First line[4]-2 2019	-0.572 (-0.715, -0.429)
Mok/OS/TPS1%/First line[4]-3 2019	-0.224 (-0.290, -0.158)
Reck/PFS/First line[32] 2016	-0.381 (-1.878, 1.116)
Gandhi/OS/Multitherapy[33]-1 2016	0.310 (0.237, 0.383)
Gandhi/PFS/Multitherapy[33]-2 2016	0.110 (0.016, 0.204)
Borghaei/OS/Monotherapy[37] 2015	-0.320 (-0.369, -0.271)
Wu/OS/Second Line[39] 2019	0.060 (0.004, 0.116)
Carbone/PFS/First line[41]-1 2017	-1.395 (-2.026, -0.764)
Carbone/OS/First line[41]-2 2017	0.061 (-0.192, 0.314)
Socinski/PFS/First line[42] 2018	-0.220 (-0.317, -0.123)
Rittmeyer/OS/Monotherapy[43] 2017	0.030 (-0.019, 0.079)
West /PFS/Multitherapy[44]-1 2019	0.260 (0.100, 0.420)
West /OS/Multitherapy[44]-2 2019	0.010 (-0.122, 0.142)
Reck/OS/ABCP vs BCP/multitherapy[45]-1 2019	0.140 (0.068, 0.212)
Reck/OS/ACP vs BCP/multitherapy[45]-2 2019	-0.140 (-0.239, -0.041)
Antonia/PFS/Second or Third line[46] 2017	0.300 (0.226, 0.374)
Antonia/OS/Second or Third line[47] 2018	0.370 (0.264, 0.476)
Butts/OS concurrent chemoradiotherapy/[25]-1 2013	-0.760 (-1.193, -0.327)
Butts/OS sequential chemoradiotherapy/[25]-2 2013	-3.830 (-8.243, 0.583)
Katakami/OS/[26] 2017	-0.100 (-0.936, 0.736)
**Overall (I^2=0 % , P< 0.001)**	**-0.039 (-0.058, -0.020)**

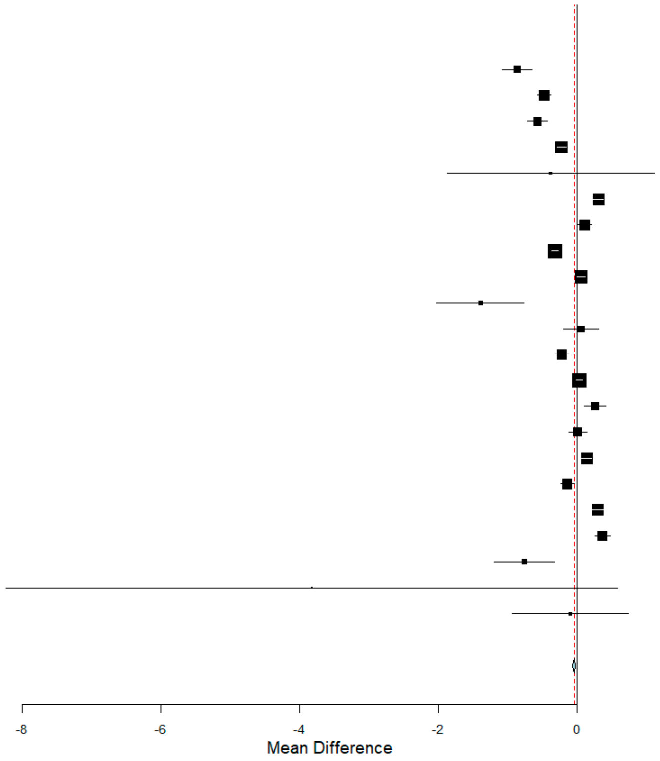

Supplemental Figure s52. Response to treatment between smoker and non-smokers when treated with anti-PD-1 and anti-MUC1 drugs using FE model in cancer patients.

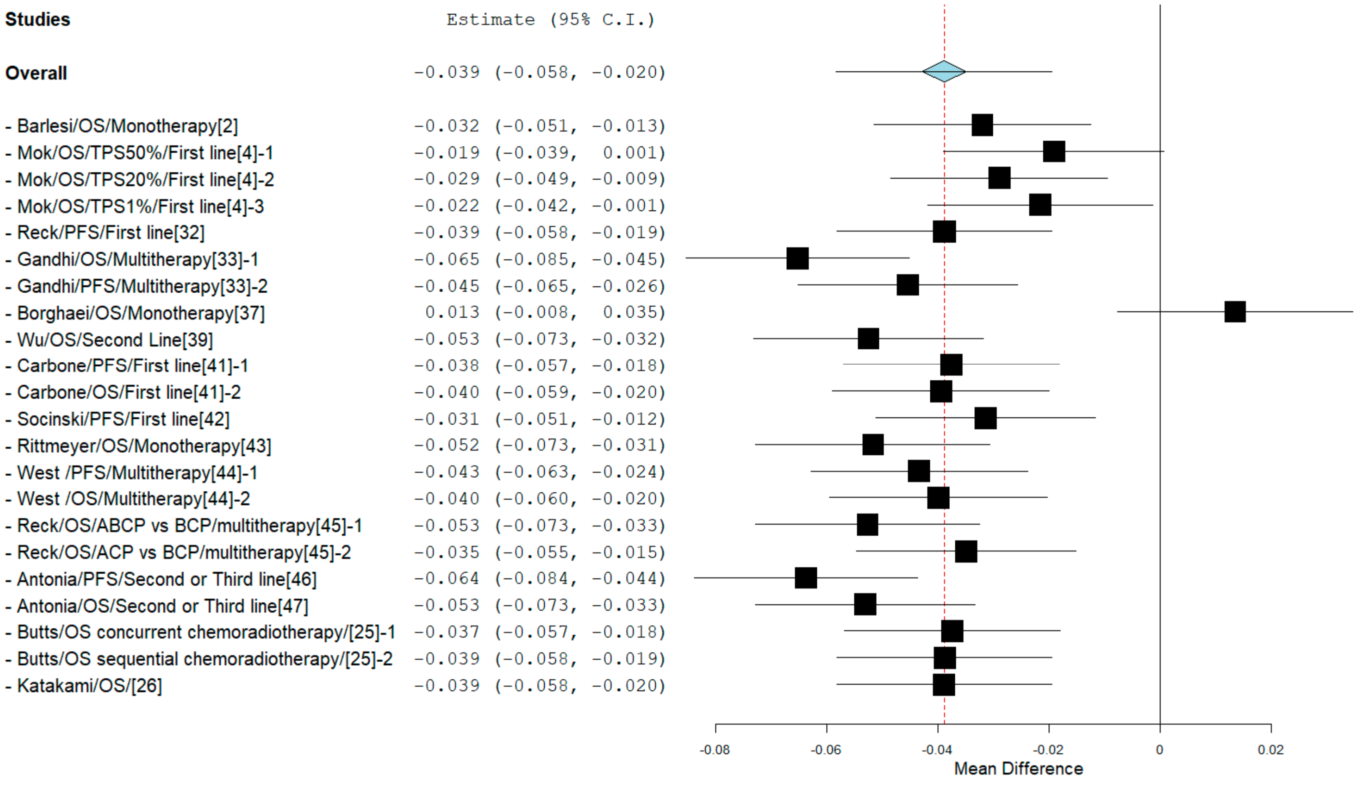

Supplemental Figure s53. Response to treatment between smoker and non-smokers when treated with anti-PD-1 and anti-MUC1 drugs using FE leaveoneout model in cancer patients.

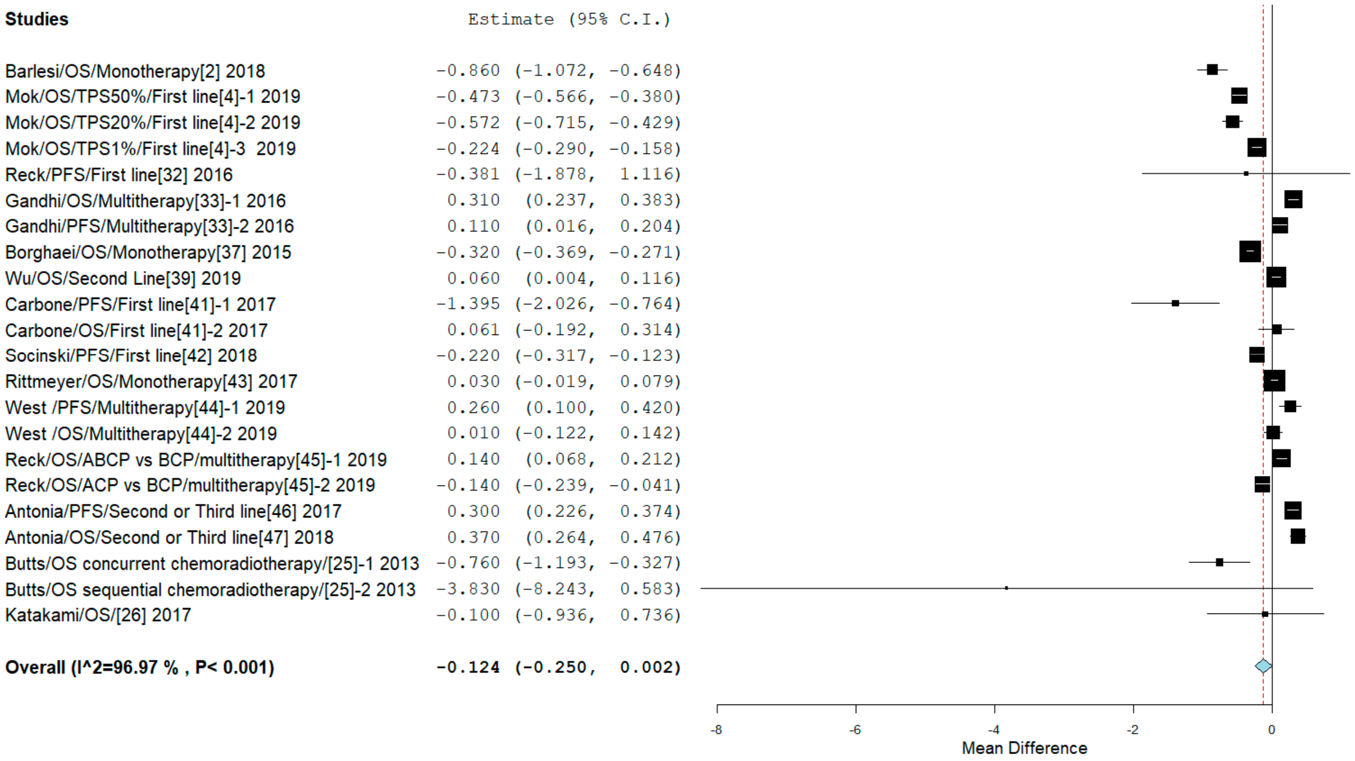

Supplemental Figure s54. Response to treatment between smoker and non-smokers when treated with anti-PD-1 and anti-MUC1 drugs using RE model in cancer patients.

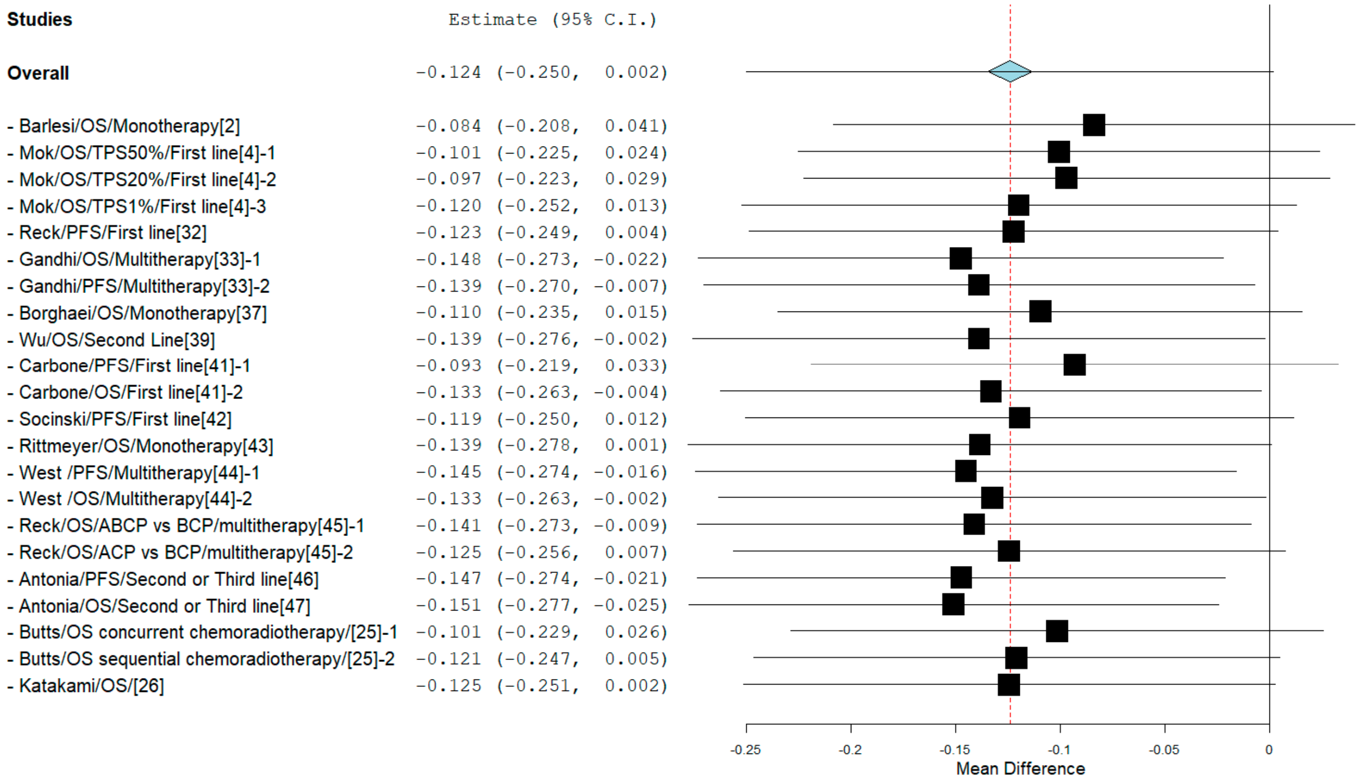

Supplemental Figure s55. Response to treatment between smoker and non-smokers when treated with anti-PD-1 and anti-MUC1 drugs using RE leaveoneout model in cancer patients.

PART E.

GFR inhibitors used in NSCLC with EGFR gene mutations

Response to EGFR inhibitors for non-squamous cell NSCLC

The effects of several drugs that target the epidermal growth factor receptor (EGFR) have been reported in clinical trials. These drugs have been used alone (without chemo) as the first treatment for advanced

NSCLCs that have certain mutations in the *EGFR* gene, or in combination with chemotherapy or other drugs. We first examined their effects on smoker and non-smoker patients to EGFR drugs. The results indicated that smokers showed a smaller responded worse than that of the non-smokers

a. Erlotinib

Erlotinib is an inhibitor of the EGFR tyrosine kinase and has been used in the treatment of NSCLCs. A total of 18 publications were obtained by searching with key words "Erlotinib phase-3" with three recent publications on the clinical trials phase 3 for NSCLC. One contained data on smoking status. A total of 141 publications of clinical trials were obtained by searching with key words "Erlotinib phase-III". Of these, 97 were studies of NSCLC.

Eventually data from 10 trials were collected [33-42] (**Tabulation S5**). Among these 10 studies, in only one trial did nonsmokers (with a relatively small number of 45 patients) have an obviously higher mean HR than the combined sample of current and former smokers in 108 patients. [38]

Median survival, months

Supplemental Tabulation S5. HR of smokers and non-smokers of NSCLC patients treated with EGFR inhibitors.

Study (First Author)/Drug	EGFR Mutation	analytic Metrix	Current/#Patients	Former/#Patients	Never/#Patients	Overall/#Patients
Wu/ combination of chemotherapy and erlotinib [33]	24%	PFS	0.77 (0.54-1.10)/131	0.87 (0.58-1.30) /101	0.40 (0.30—0.54) /219	0.57 (0.47-0.69) /650
Saito/ Erlotinib plus bevacizumab versus erlotinib/ [34]	56%	PFS	0.63 (0.35-1.11) /82	2.95 (0.30-28.5) /13	0.54 (0.33-0.90)/129	0.63/224

Rosell/ Erlotinib versus standard chemotherapy [35]	100%	PFS	0.56 (0.15-2.15) /19	1.05 (0.40-2.74) /34	0.24 (0.15-0.39) /120	0.37(0.25-0.54) /173
Ciuleanu/ erlotinib versus chemotherapy in second-line treatment [36]	67-70%	OS	0.90 (0.81-1.19) /227	1.10 (0.73-1.66) /123	0.86 (0.94-1.51) /74	0.96 (0.78-1.19) /424
Zhou/ Erlotinib versus chemotherapy as first-line [37]	100	PFS	0.21(0.09-0.49) /45		0.14(0.08-0.25) /109	0.16 (0.10-0.26) /154
Zhou/ Erlotinib versus chemotherapy as first-line [38]	100	OS	0.85(0.44-1.64)/109		1.44(0.93-2.24)/45	1.19(0.83-1.71)/154
Cappuzzo/ Erlotinib as maintenance treatment [39]	55-58%	PFS	0.80 (0.67-0.97) /490	0.66 (0.50-0.88) /242	0.56 (0.38-0.81) /152	0.71 (0.62-0.82) /884
		OS	0.88 (0.72-1.08) /493	0.75 (0.56-1.00) /244	0.69 (0.45-1.05) /152	0.81(0.70-0.95) /889
Kelly/ Erlotinib Versus Placebo [40]	16-20%	disease-free surviva l	0.79(0.446-1.406)/111	0.93(0.72 4-1.185)/66 3	0.91(0.596-1.387)/199	0.90(0.741-1.104)/973

Kawaguchi/ erlotinib versus docetaxel as second- or third [41]	All wild type	PFS	1.20(0.91-1.56)/225		1.37(0.83-2.23)/76	1.22(0.97-1.55)/301
Gridelli/ erlotinib followed by second-line cisplatin-gemcitabine [42]	39 positive /236 negative	OS	1.27(1.06-1.54)/603		1.08(0.72-1.61)/157	1.22(1.03-1.44)/760
		PFS	1.24(1.04-1.48)/603		1.01(0.70-1.43)/157	1.19(1.01-1.39)/760
Miller/ Afatinib versus placebo/after failure of erlotinib, gefitinib, or both [43]	62 Positive 31 Negative	OS	0.81 (0.56-1.17) /118 (current and former)	2.19 (0.74-6.48) /13 (existing light)	1.20 (0.90-1.61) /245	1.08 (0.86-1.35) /390
		PFS	0.46 (0.32-0.68) /118	0.30 (0.12-0.71) /27	0.36 (0.28-0.48) /245	0.38 (0.31-0.48) /390
Wu/ Afatinib versus cisplatin plus gemcitabine for first-line [44]	100%	PFS	0.39(0.07-2.41)/12	0.46 (0.22-1.00) /72	0.24 (0.16-0.34) /280	0.28 (0.20-0.39) /364
Soria/ Afatinib versus erlotinib as second line [45]	Not reported	PFS	0.85 (0.72-1.01) 728	0.44(0.14-1.37)/23	0.55 (0.27-1.11)/44	0.81(0.69-0.96)/795
		OS	0.81(0.69-0.96)	0.43(0.16-1.12)/	0.77 (0.37-1.57)/	0.81(0.69-0.95)
Shi/ Icotinib versus gefitinib in previous treated [46]	43-59%	PFS	0.96 (0.71-1.31) /192		0.72 (0.52-1.00) /203	0.83(0.67-1.05)397

Zhang/ Gefitinib versus placebo [47]	>=15%	PFS	0.52 (0.35-0.75) /136	0.36 (0.25-0.51) /160	0.42 (0.33-0.55) /296
Mitsudomi/ Gefitinib versus cisplatin plus docetaxel [48]	100%	PFS	0.575 (0.294-1.123) /54	0.466 (0.297-0.732)/118	0.489 (0.336-0.710) /172
Zhong/ Gefitinib versus vinorelbine plus cisplatin [49]	99-100%	PFS	0.56 (0.27-1.19) /52	0.61 (0.40-0.92) /167	0.58 (0.40-0.83) /222
Soria/ Osimertinib in Untreated EGFR-Mutated [50]	All Mutation	PFS	0.48(0.34-0.68)/199	0.45(0.34-0.59)/357	0.46(0.37-0.57)/556
Mok / Osimertinib or Platinum-Pemetrexed [51]	All mutation	PFS	0.40(0.27-0.62)/136	0.36(0.26-0.49)/283	0.37(0.29-0.48)/419
Ellis/ Dacomitinib compared with placebo [52]	>=24-28% Plus *KRAS* mutation	PFS	0.80(0.66-0.98)	0.51 (0.39-0.67)	-
		OS	1.13 (0.91-1.40) /456	0.74 (0.56-0.98)/264	-
Wu/ Dacomitinib versus gefitinib [53]	Mutation positive	PFS	0.72 (0.49-1.05) /161	0.51 (0.39-0.68) /291	0.58 (0.46-0.73) /452
Ramalingam/ Dacomitinib versus erlotinib previous treated [54]	>=10-14%	PFS (all patients)	0.98 (0.82-1.16) /717	0.76 (0.52-1.13) /161	0.94 (0.80-1.10) /878

		atients with KRAS wild type	1.02 (0.81-1.27)		0.93(0.57-1.50)	1.02 (0.83-1.25)
		OS	1.08 (0.90-1.29) /717		0.85 (0.54-1.34) /161	1.08/878
Thatcher / Necitumumab plus gemcitabine and cisplatin [55]	EGFR expression was high (H-score ≥200) in 374 (38%) of 982 cases	OS	0.85 (0.74-0.98)/781		0.82 (0.52-1.29)/78	0.84 (0.74-0.96) /1093
		PFS	0.85 (0.74-0.98) /776		0.88 (0.55-1.40) /72	0.85 (0.74-0.98) /1093
Paz-Ares / Necitumumab plus pemetrexed and cisplatin [56]	490 patients with high expression	OS	1.05 (0.85-1.30)/423	0.81 (0.59-1.27)/101	1.04 (0.66-1.64) /109	1.01 (0.84-1.01) /633

b.Afatinib (Gilotrif)

Afatinib is a tyrosine kinase inhibitor used to treat NSCLC that has advanced to include metastasized tumors with mutations of EGFR. A total of 6 publications were obtained by searching with key words “Afatinib phase-3”, for clinical trials. Two of them are for NSCLC and contained analysis of the effect of smoking

status. A total of 32 publications were obtained by searching with key words “Afatinib phase-III”, for clinical trials. However, based on the criteria, no additional qualified publications were identified.

In one report by Miller et al, PFS and OS showed a mixture of HR values of current and former smokers in comparison to that of non-smokers [43] while the other study showed that smokers showed higher or similar mean HR values than non-smokers [44] (**Tabulation S5**).

c. Gefitinib (Iressa)

Gefitinib is another EGFR inhibitor, like erlotinib, which interrupts cancer signaling through the EGFR. A total of 20 publications were obtained by searching with key words “Gefitinib phase-3”, for clinical trials. Two of them are for NSCLC and reported the effect of smoking status. A total of 87 publications were obtained by searching with key words “Gefitinib phase-III”, for clinical trials. Three additional studies for NSCLC contained an analysis of smoking status. In four of these, the HR values of smokers were higher than that of non-smokers, [45-48] while in the other study, mean HR was similar between smokers and non-smokers [49] (**Tabulation S5**).

d. Osimertinib

Osimertinib is a third-generation EGFR tyrosine kinase inhibitor. A total of three publications were obtained by searching with key words “Osimertinib phase-3” for clinical trials. Two of them were for NSCLC and contained analysis of smoking status. Searching with “Osimertinib phase-III” for clinical trials resulted in four publications, but no new smoking data were obtained. In these two studies, the mean HR of non-smokers was similar to that of smokers [50, 51] (**Tabulation S5**).

e. Dacomitinib

Dacomitinib is a selective and irreversible inhibitor of EGFR. A total of three reported clinical trials that included analysis of smoking status were obtained by searching with key words “Dacomitinib phase-3” for NSCLC. [52-54] Searching with “Dacomitinib phase-III” for clinical trials resulted in five publications, but no

new smoking data were obtained. In all three studies, mean HR values of smokers are higher than that of non-smokers [52-54] (**Tabulation S5**).

The majority of these trials tested one drug alone or compared one with another. Therefore, these data demonstrated that non-smokers responded to EGFR inhibitors better than smokers.

Response to EGFR inhibitor Necitumumab, for squamous cell NSCLC

Necitumumab has been used along with chemotherapy as the first treatment in people with advanced squamous cell NSCLC. A total of five publications were obtained by searching with key words "Necitumumab phase-3", for clinical trials. Two of them are for NSCLC and include data for smoking status. Searching with "Necitumumab phase-III" for clinical trials resulted publications, but these did not include smoking status data were. In these two studies, the mean HR values between smokers and non-smokers were similar (**Tabulation S5**). [55, 56]

Meta-analysis of EGFR inhibitors used in NSCLC with EGFR gene mutations

We next analyzed separately the HR values from patients treated with EGFR (**Tabulation s6**). Together, the non-smokers had a mean HR of 0.027 which was lower

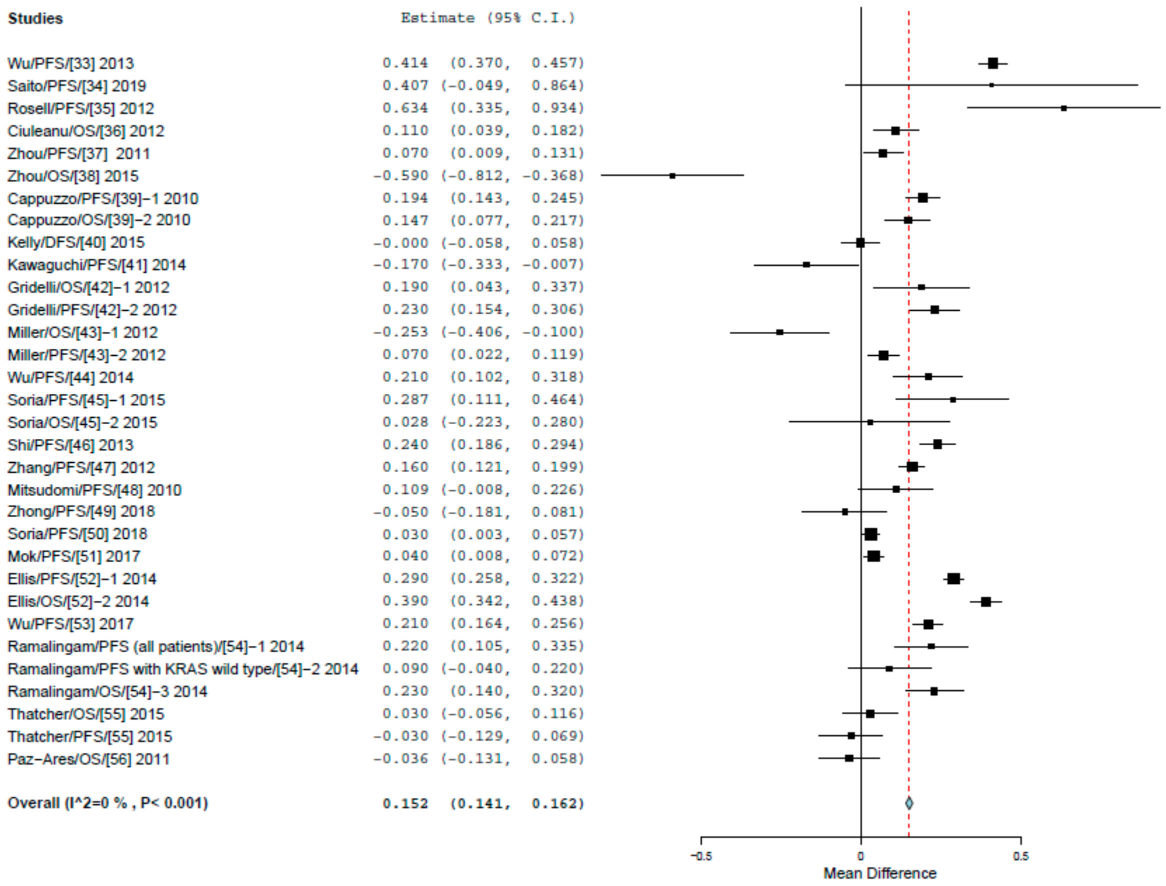

Supplemental Figure s56. Smokers vs non-smokers in response to treatment with EGFR drugs with fixed effect model (FE)

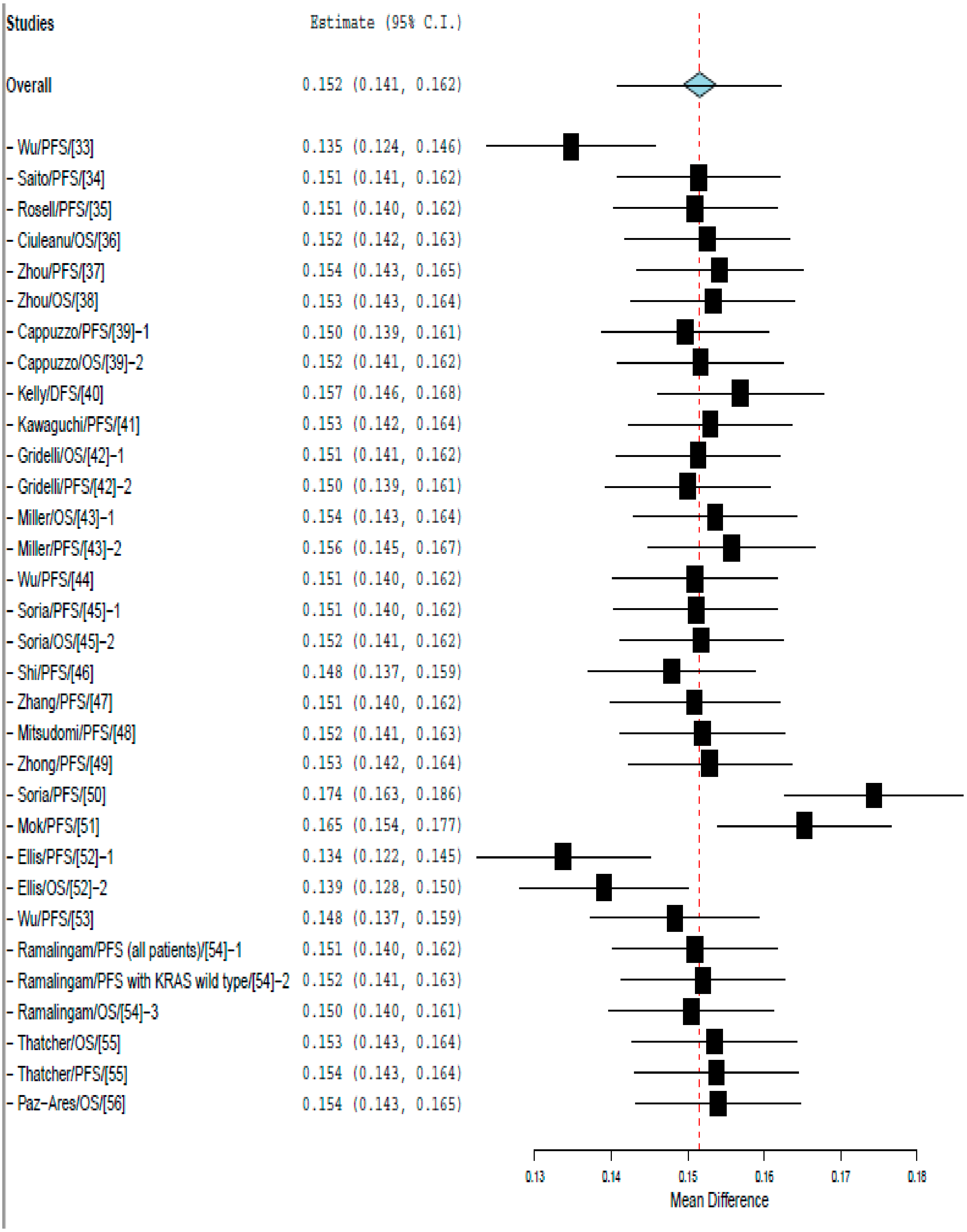

Supplemental Figure s57. Smokers vs non-smokers in response to treatment with EGFR drugs with FE

leaveoneout model

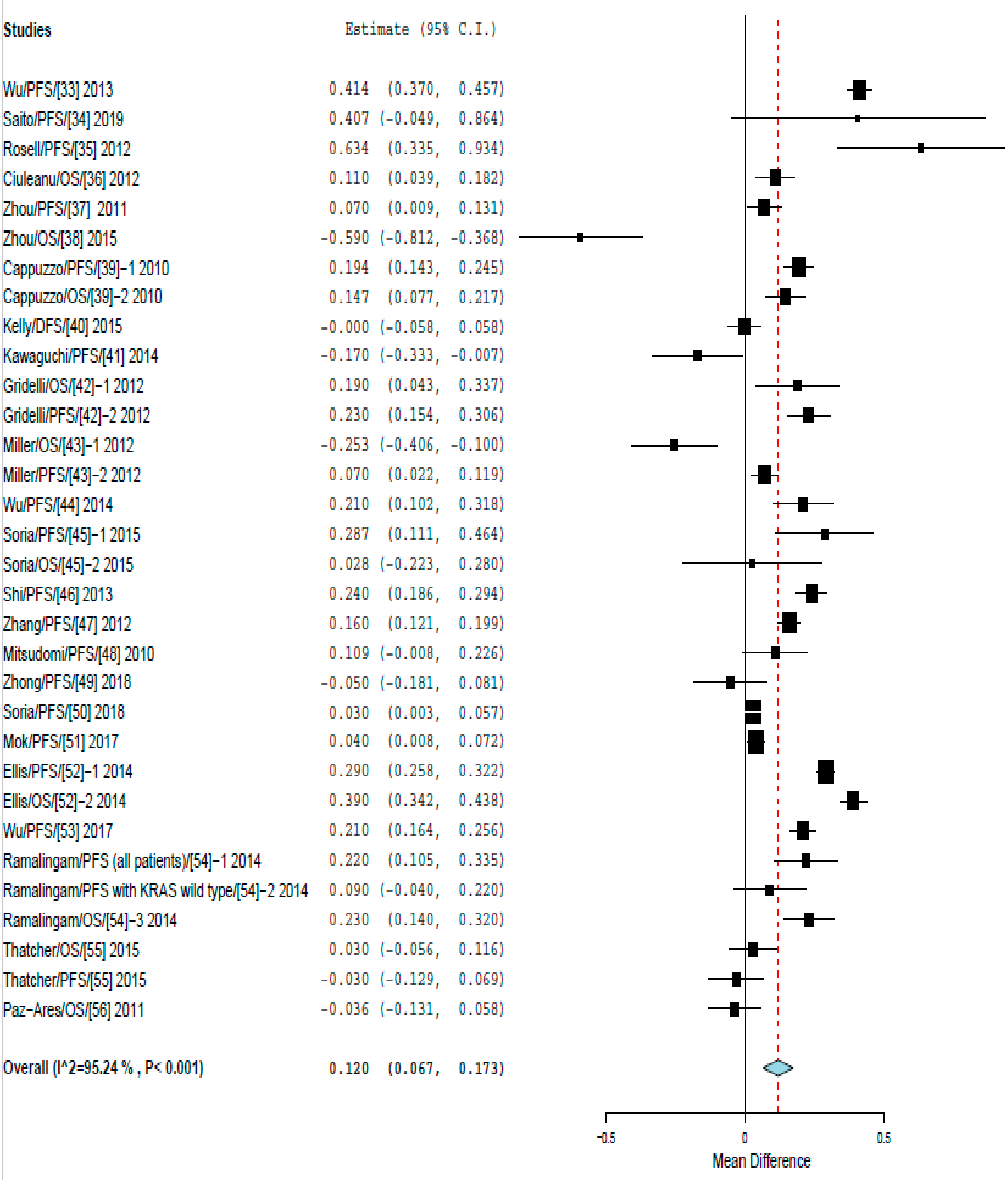

Supplemental Figure s58. Smokers vs non-smokers in response to treatment with EGFR drugs with random

effect (RE) model

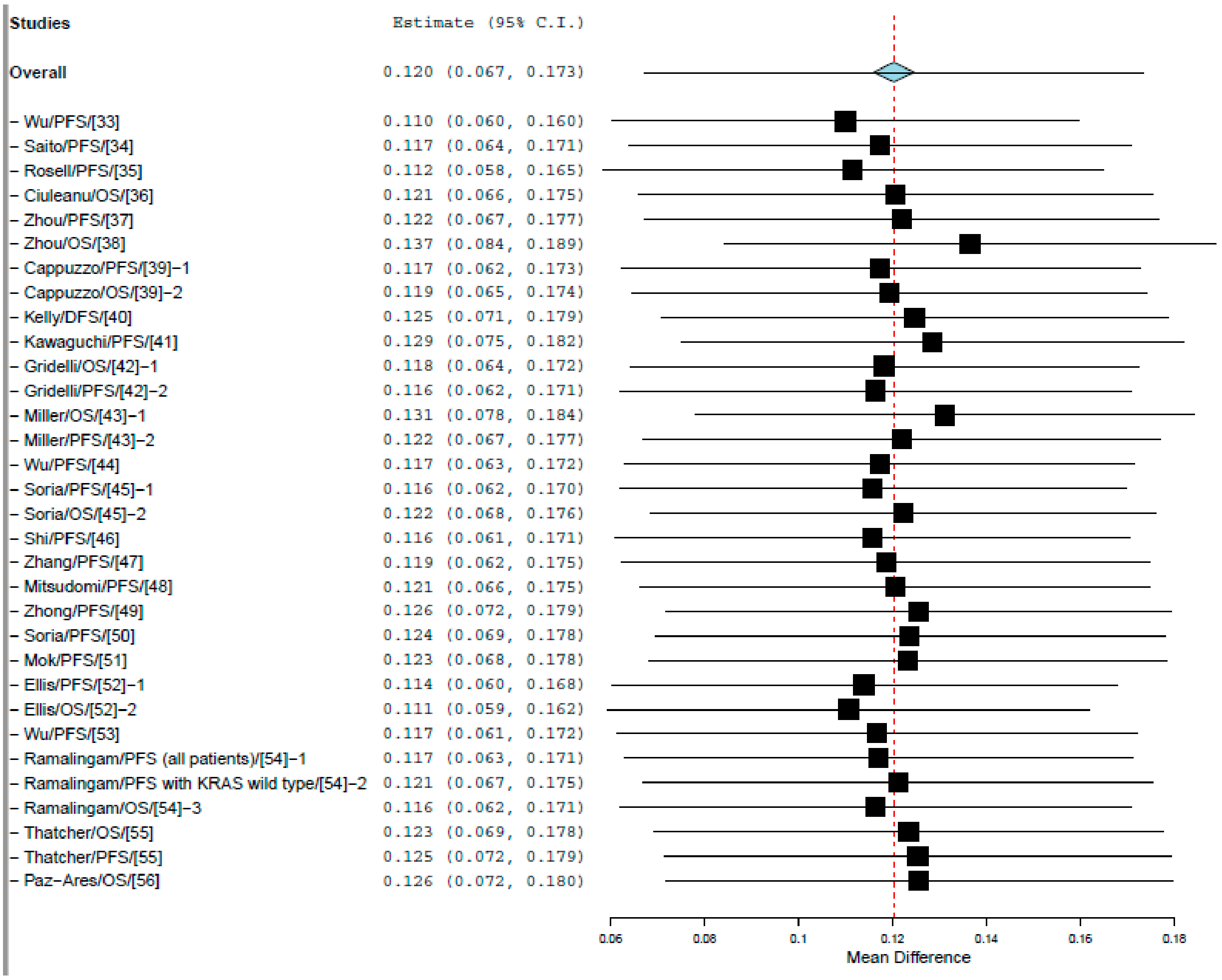

Supplemental Figure s59. Smokers vs non-smokers in response to treatment with EGFR drugs with RE leaveoneout model

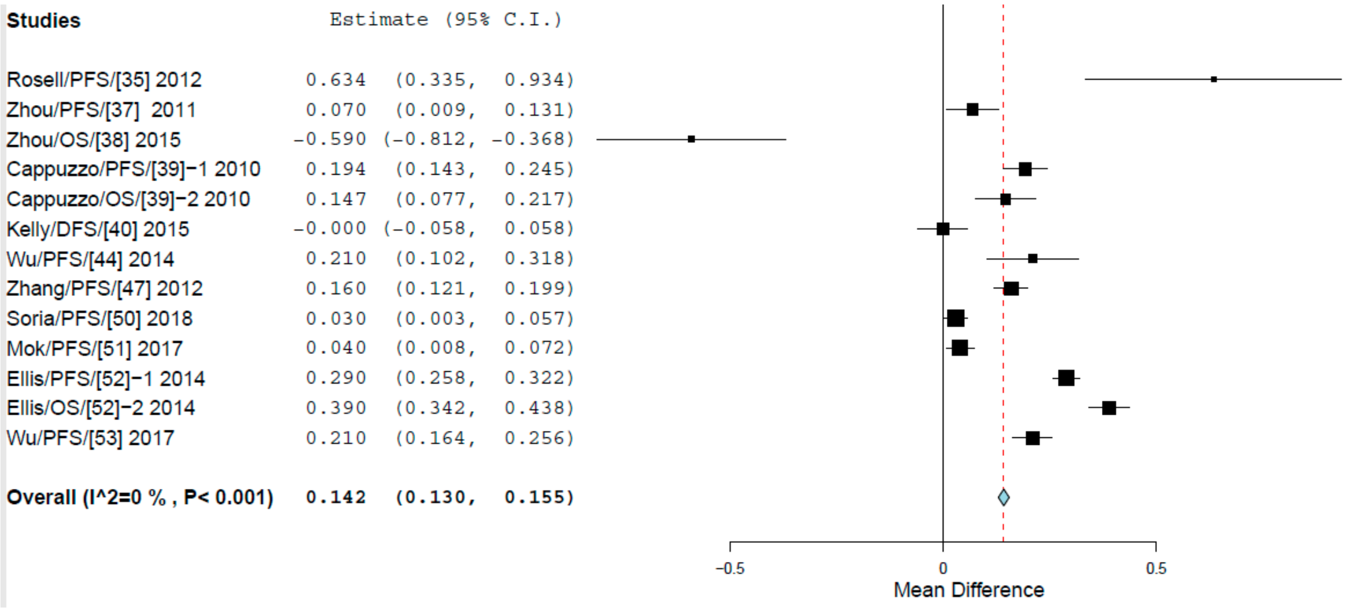

Supplemental Figure s60. Smokers vs non-smokers in response to EGFR drugs as the first-line treatment with fixed effect model (FE)

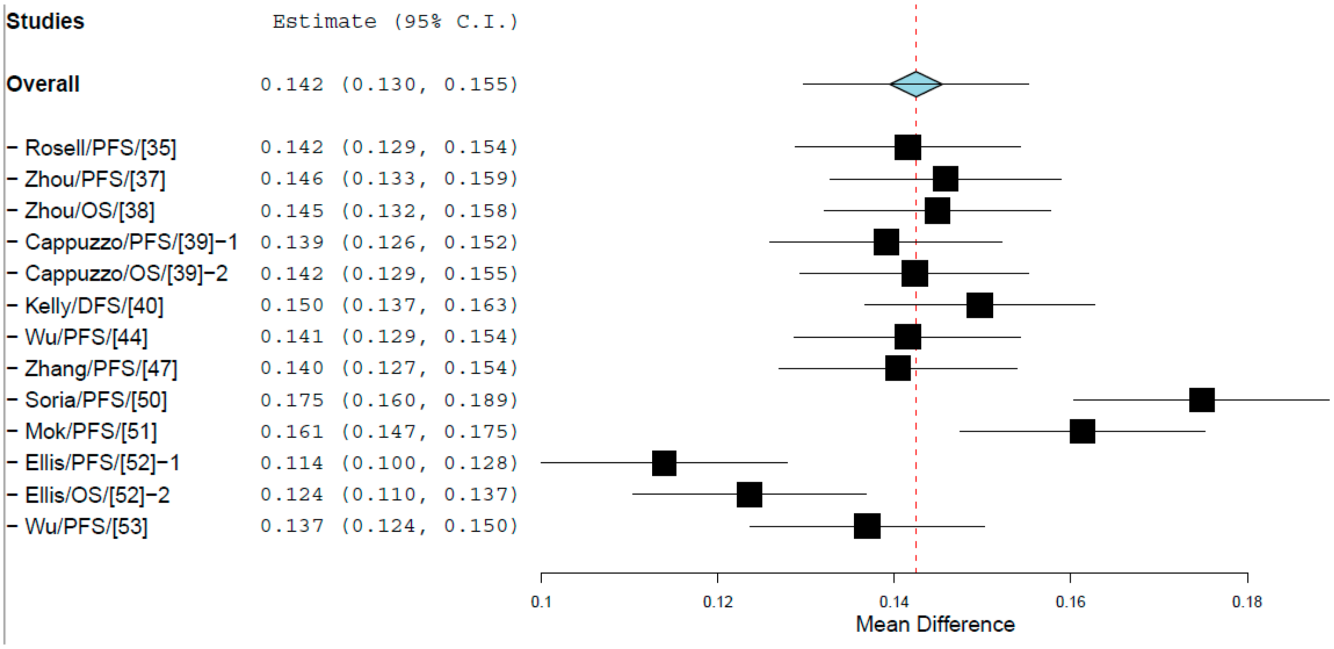

Supplemental Figure s61. Smokers vs non-smokers in response to EGFR drugs as the first-line treatment with FE leaveoneout model

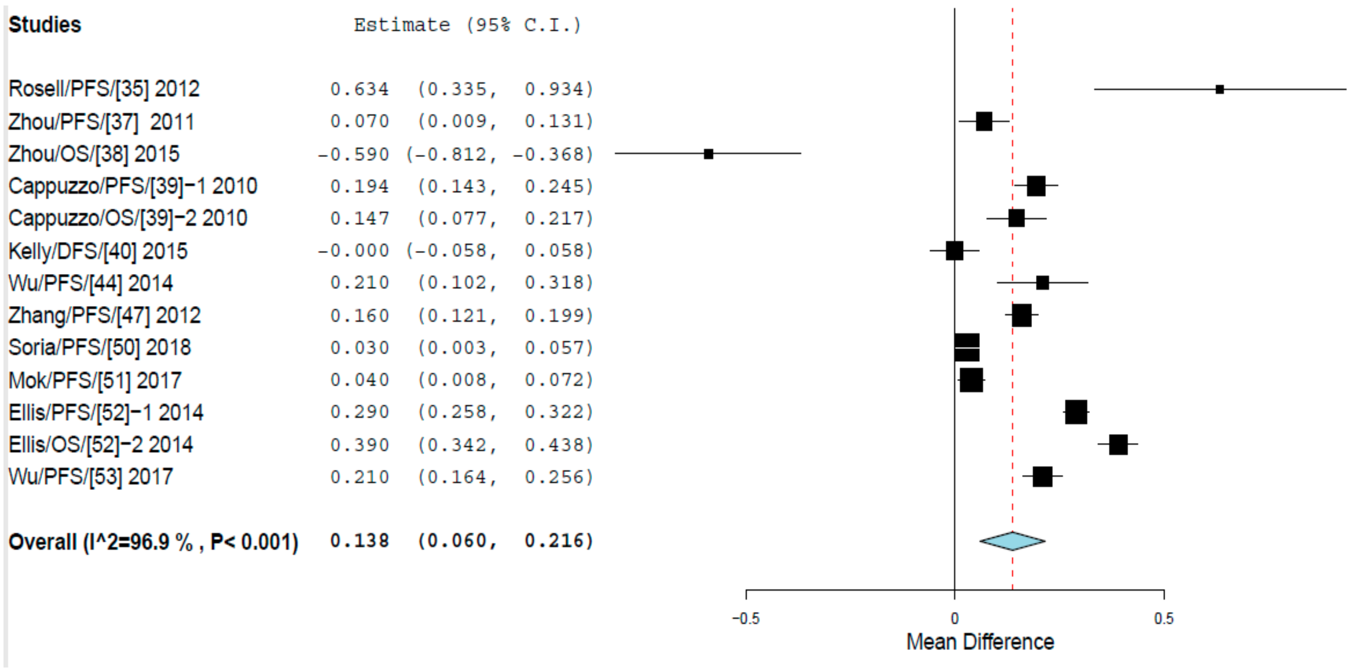

Supplemental Figure s62. Smokers vs non-smokers in response to EGFR drugs as the first-line treatment with random effect model (RE)

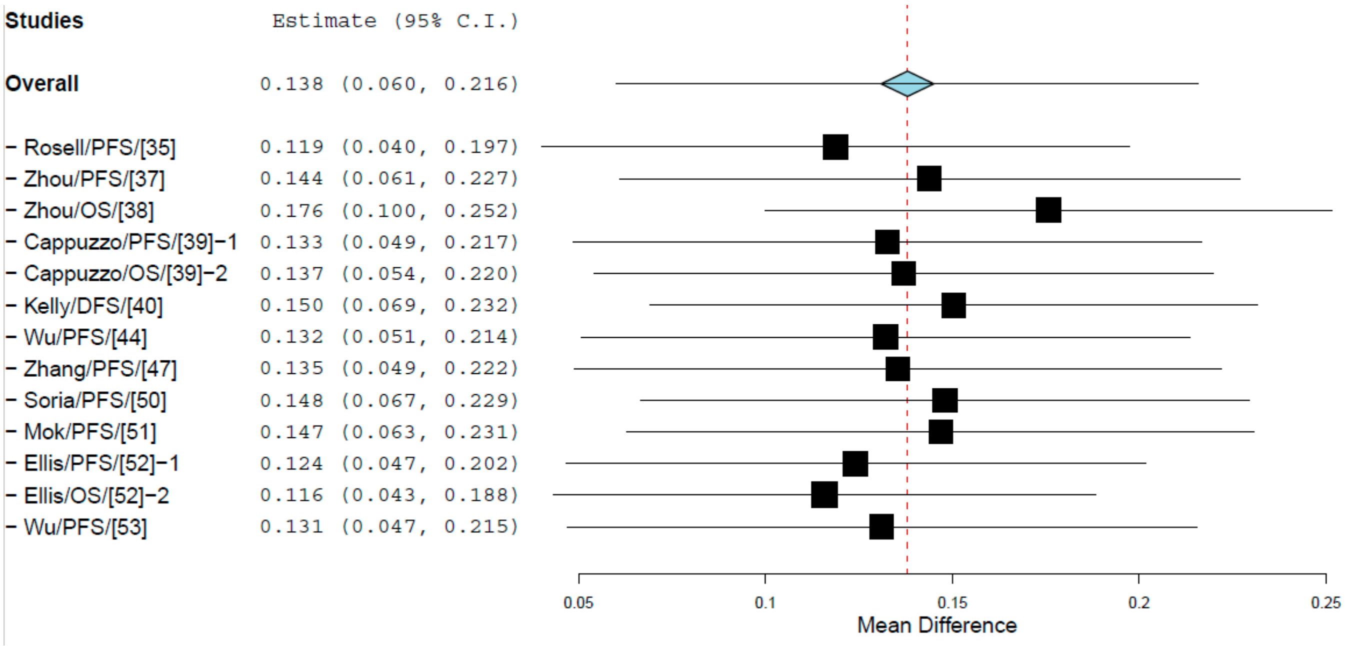

Supplemental Figure s63. Smokers vs non-smokers in response to EGFR drugs as the first-line treatment with RE leaveoneout model

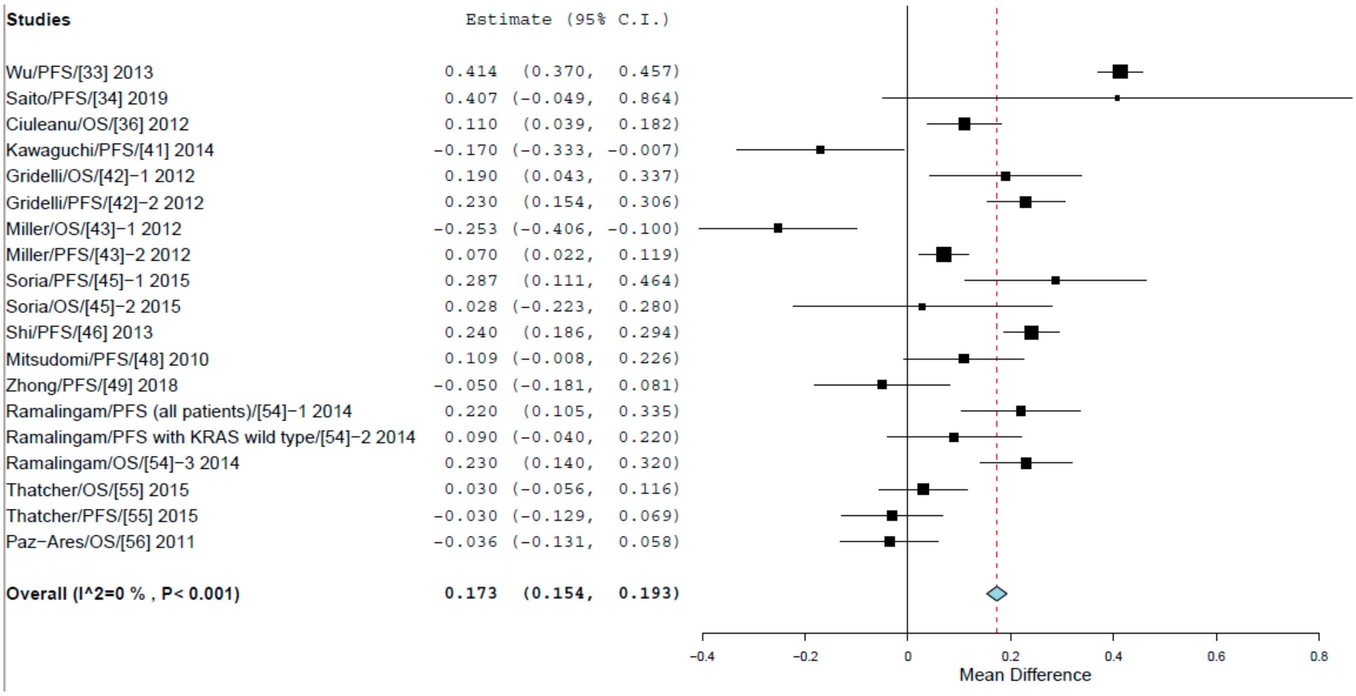

Supplemental Figure s64. Smokers vs non-smokers in response to EGFR drugs in multiple drug treatment with Fixed effect (FE) model

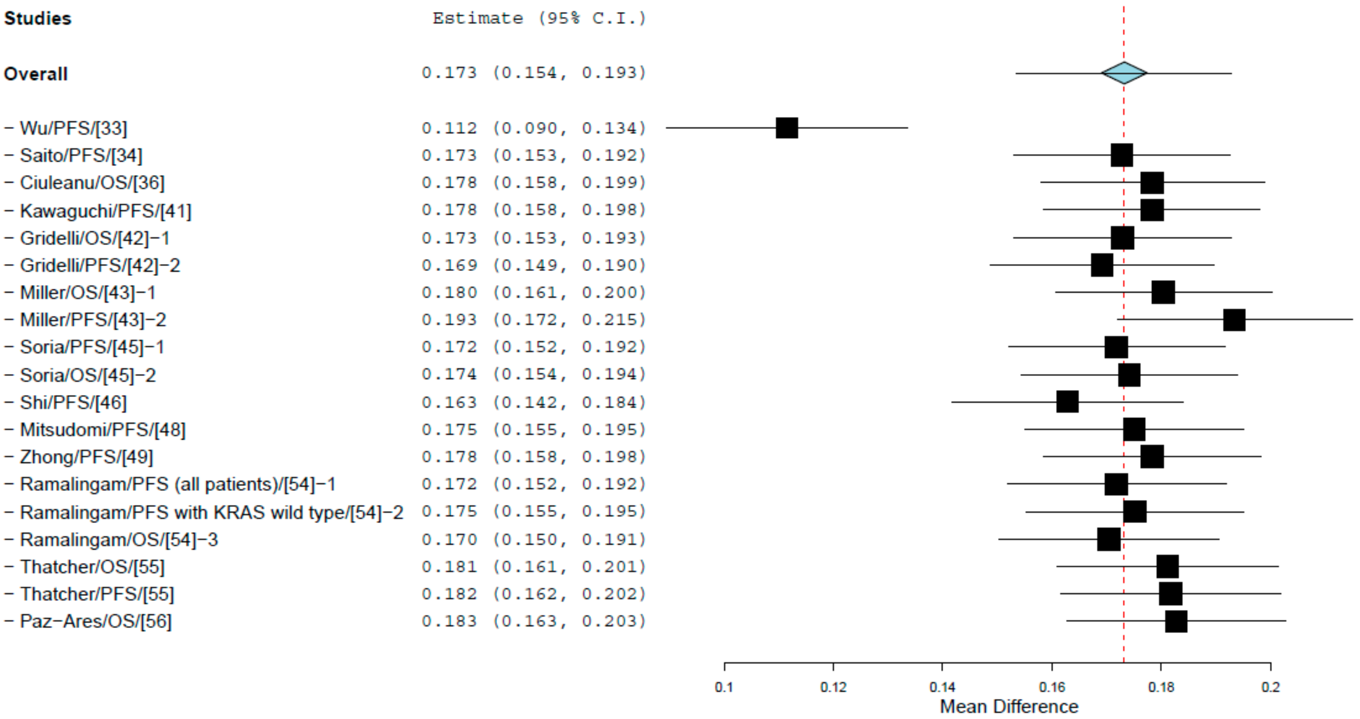

Supplemental Figure s65. Smokers vs non-smokers in response to EGFR drugs in multiple drug treatment with FE leaveoneout model

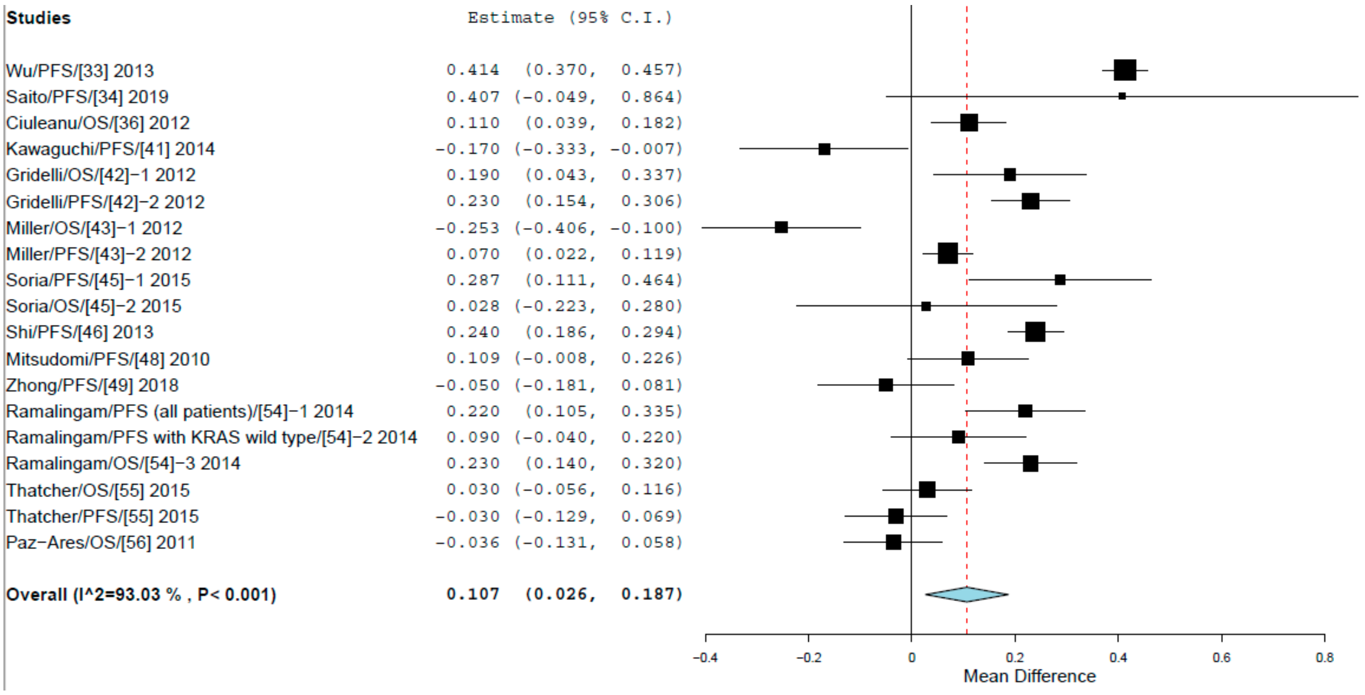

Supplemental Figure s66. Smokers vs non-smokers in response to EGFR drugs in multiple drug treatment with random effect (RE) model

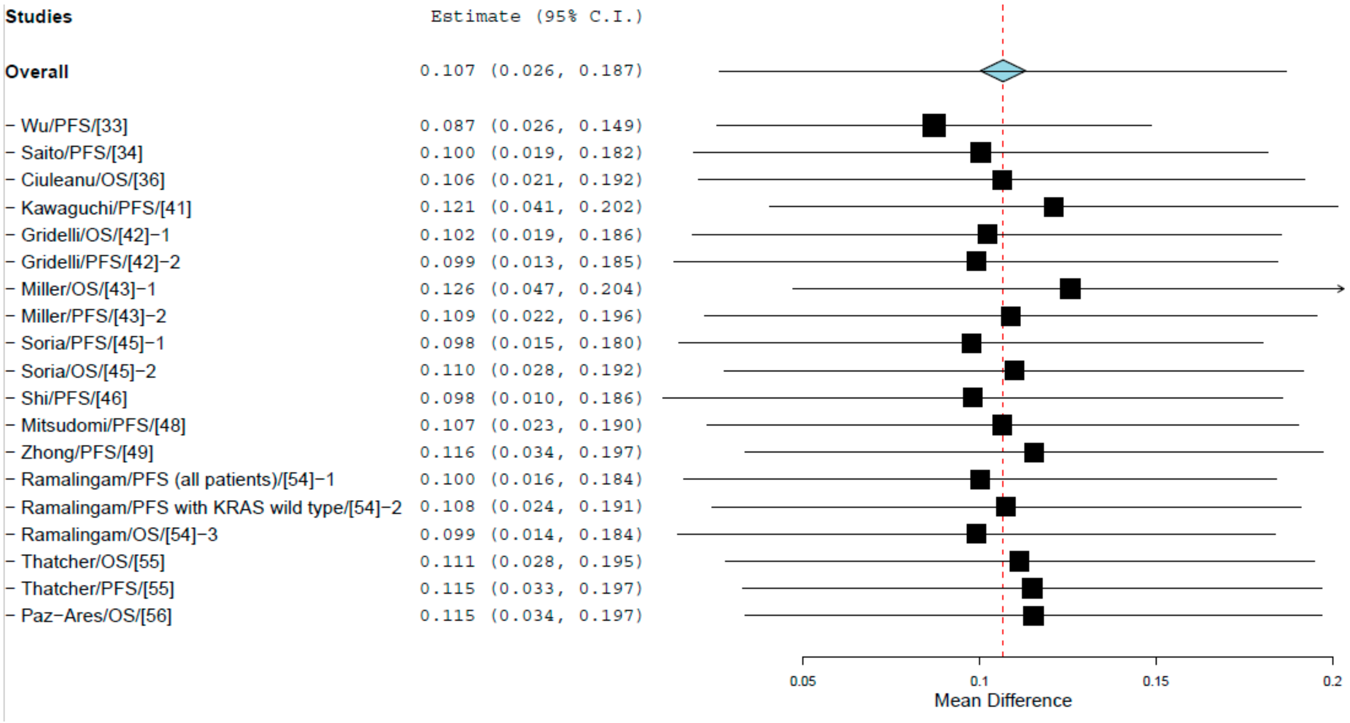

Supplemental Figure s67. Smokers vs non-smokers in response to EGFR drugs in multiple drug treatment with RE leaveoneout model

Meta-analysis of EGFR inhibitors used in NSCLC with VEGF gene mutations

We next analyzed separately the HR values from patients treated with VEGF (**Tabulation s6**). Together, the non-smokers had a mean HR of 0.027 which was lower

Supplemental Tabulation S6 HR of smokers and non-smokers of NSCLC patients treated with drugs targeting VEGF

Study (First Author)/Drug	VEGF Mutation	analytic Metrix	Current/# Patients	Former/# Patients	Never/#Patients	Overall/#Patients

Herbst/ bevacizumab plus erlotinib versus erlotinib/ second treatment [57]	~20%	OS	1.06 (0.87-1.29) /569		0.44 (0.21-0.94) /67	0.97 (0.80-1.18) /636
Wakelee/ chemotherapy with or without bevacizumab [58]	Not Found	OS	0.99 (0.82-1.19) /1344		0.84 (0.36-1.96) /155	0.99 (0.82-1.19) /1501
		Disease-free survival	0.99 (0.85-1.16) /1344		0.82 (0.46-1.45) /155	0.99 (0.86-1.15) /1501
Barlesi/ maintenance bevacizumab wi th or without pemetrexed [59]	Not found	PFS	0.57(0.41-0.80)/188		0.40(0.21-0.74)/64	0.48(0.35-0.66)/253
Johnson / bevacizumab with or without erlotinib/, after completion of chemotherapy, with bevacizumab for first-line [60]	*VEGF* *m*utation status was not required	PFS	0.74(0.54-1.01)/258	0.79(0.61-1.03)/358	0.34(0.19-0.61)/127	-

Part F. NSCLC Patients response to treatment by drugs that target tumor blood vessel growth (angiogenesis)

Two angiogenesis inhibitors, Bevacizumab (Avastin) and Ramucirumab (Cyramza), have been used in the treatment of NSCLC.

Bevacizumab is a monoclonal antibody that targets vascular endothelial growth factor (VEGF). A total of 86 publications were obtained by searching with key words “Bevacizumab phase-3” for clinical trials. Twelve of them studied NSCLC and three included analysis of smoking status. Searching with “Dacomitinib phase-III” for clinical trials resulted in 60 publications, however only one with smoking data was obtained. Among these four studies, the HR values of smokers were higher than those of non- smokers [57-60] (**Tabulation S7**).

Ramucirumab is a monoclonal antibody that targets a VEGF receptor. A total of 13 publications were obtained by searching with key words “Ramucirumab phase-3” for clinical trials. No data for NSCLC studies included smoking status. Searching with “Ramucirumab phase-III” for clinical trials resulted in 30 publications, but none included smoking status data.

Meta-analysis of drugs that target tumor blood vessel growth

We next analyzed HR values from patients treated with VEGF inhibitors (**Tabulation S7**). Together, the non-smokers had a mean HR of 0.148 compared to 0.226 for smokers. This result indicated that smokers responded worse than non-smokers (**Fig. S7**).

Tabulation S7. HR values of smokers and non-smokers of NSCLC patients treated with drugs targeting ALK gene

Drug	(First Author)/Method	analytic Metrix	Current/ #Patients	Former/# Patients	Never/#Patients	Overall/#Patients
Crizotinib	Wu/ versus Chemotherapy/east Asian [61]	PFS	0.622(0.338-1.147)57		0.323(0.216 -0.484)/150	0.402(0.286 -0.565)/207
	Solomon/ Versus Chemotherapy/ ALK-final	Cox proportional hazards regression model	0.689(0.422-1.127)/125		0.888(0.584 -1.350)/218	0.760(0.548 -1.053)/343

	Mutation-Positive [62]					
	Solomon/ versus chemotherapy /ALK-positive [63]	PFS	0.64(0.42-0.97)/125		0.41(0.29-0.58)/218	0.45(0.35-0.60)/343
	Shaw/ versus chemotherapy/ ALK-positive [64]	PFS	0.53(0.34-0.83)/127		0.45(0.32-0.63)/219	0.49(0.37-0.64)347
Ceritinib	Shaw/ versus chemotherapy/ ALK-rearranged/previous treated w/crizotinib [65]	PFS	0.68(0.40-1.14)/95		0.41(0.27-0.63)/132	0.49(0.36-0.67)/231
	Soria/ versus platinum-based/ ALK-rearranged [66]	PFS	0.48(0.30-0.77)/146		0.56(0.38-0.80)/230	0.55(0.42-0.73)/376
Alectinib	Peters/ versus Crizotinib/ Untreated ALK-Positive [67]	PFS	1.16(0.35-3.90)/17	0.42(0.23-0.77)/96	0.44(0.29-0.66)/190	0.48(0.35-0.66)/303
	Hida/ versus crizotinib/	PFS	0.18(0.08-0.42)/90		0.50(0.28-0.89)/117	0.34(0.21-0.54)/207

	ALK-positive [68]					
	Camidge/ Untreated ALK-Positive [69]	PFS	1.16(0.35-3.90)17	0.40(0.23-0.69)/96	0.40(0.27-0.59)/190	0.43(0.32-0.59)/303
Brigatinib	Camidge/versus Crizotinib in ALK-Positive [70]	PFS	0.51(0.27-0.97)/105		0.47(0.27-0.84)/159	0.49(0.33-0.74)/275

Results of meta-analysis.

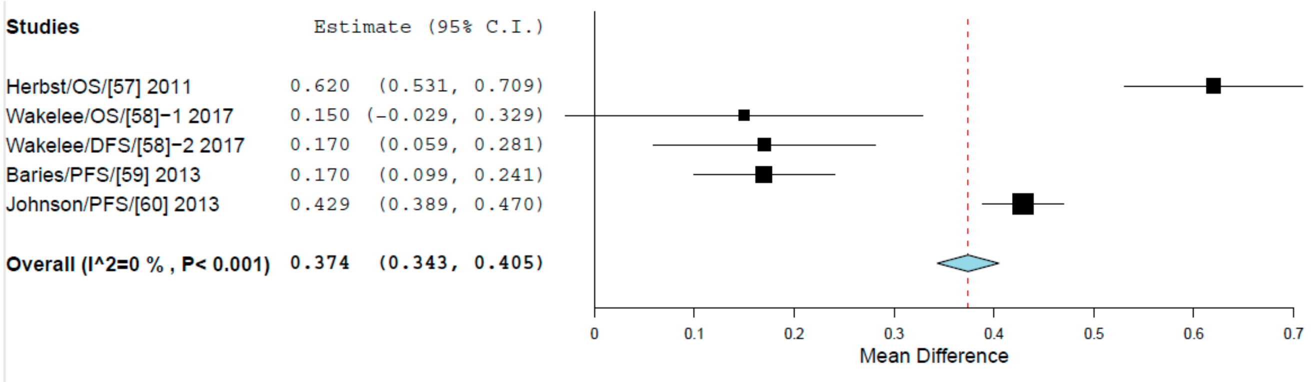

Supplemental Figure s68. Smokers vs non-smokers in response to treatment with drugs of angiogenesis with fixed effect model (FE)

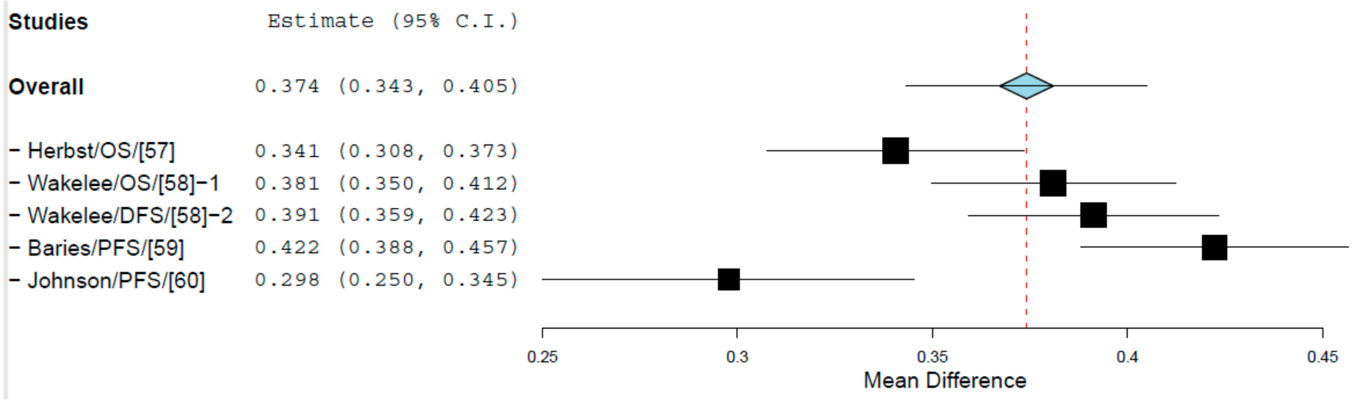

Supplemental Figure s69. Smokers vs non-smokers in response to treatment with drugs of angiogenesis with FE leaveoneout model

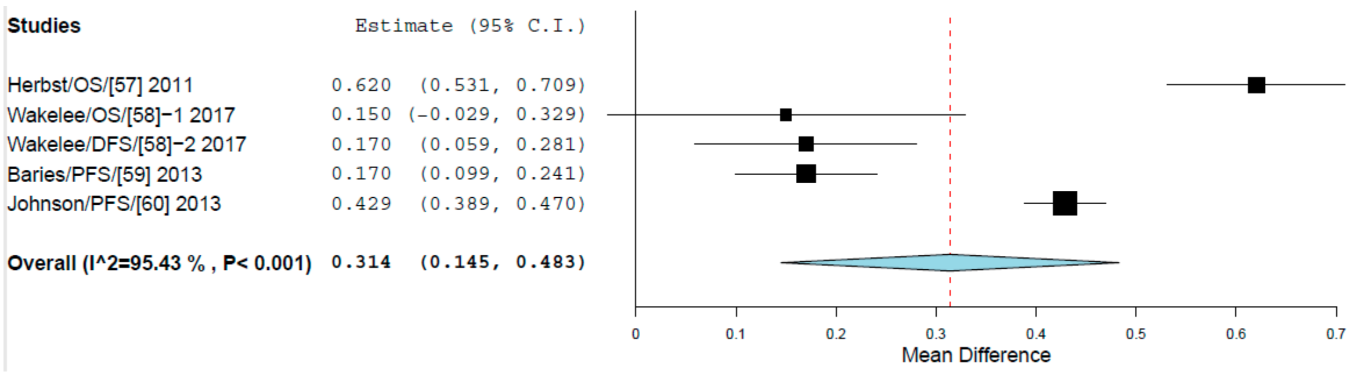

Supplemental Figure s70. Smokers vs non-smokers in response to treatment with drugs of angiogenesis with random effect model

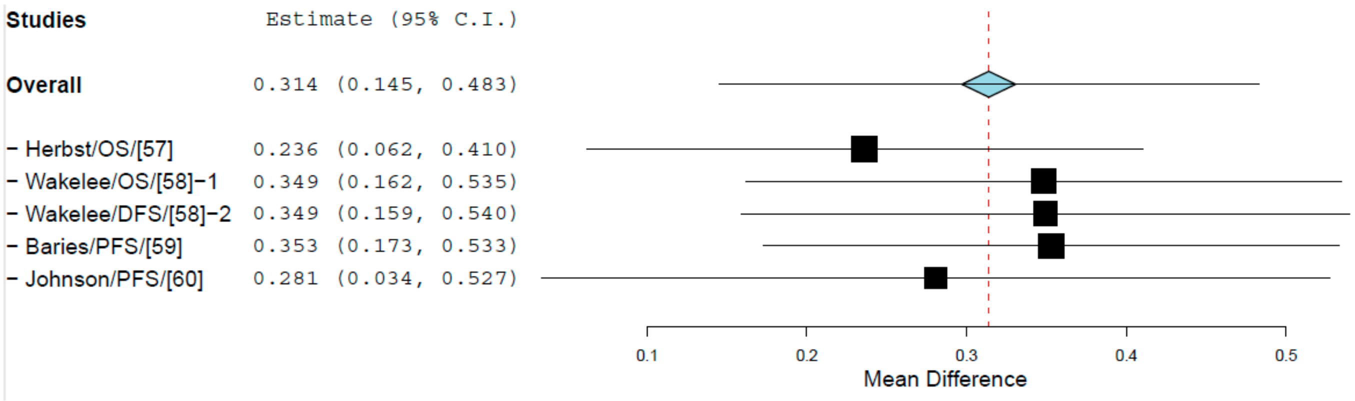

Supplemental Figure s71. Smokers vs non-smokers in response to treatment with drugs of angiogenesis with RE leaveoneout model

Part G. NSCLC Patients response to treatment by drugs targeting BRAF gene and ALK gene

G1. Drugs that target cells with BRAF gene changes

In some NSCLCs, affected cells show changes in the v-raf murine sarcoma viral oncogene homolog b1 (BRAF), Dabrafenib (Tafinlar) and Trametinib (Mekinist)

a.Dabrafenib (Tafinlar)

Thirty publications were found with key words "Dabrafenib phase-III" in the clinical trial category. Eight were found using f "Dabrafenib phase-3" in the clinical trial category. No data on smokers vs non-smokers were found.

b. Trametinib (Mekinist)

Sixteen publications were found with key words "Trametinib phase-III" in the clinical trial category, and nine using "Trametinib phase-3". No data on smokers vs non-smokers were found.

G2. Drugs that target cells with ALK gene changes

About 5% of NSCLCs show an alteration in the ALK gene. Ceritinib is a next-generation ALK inhibitor. It has shown anti-tumour efficacy with ALK-rearranged in NSCLC. Crizotinib was the first ALK-targeted therapy for patients with ALK-rearranged NSCLC, and it has become the standard of treatment in many areas.

a. Crizotinib

15 publication were found with keywords "Crizotinib phase-III" in the clinical trial category, andninefrom "Crizotinib phase-3" in theclinical trial category, which included four reporting HRdata for smokers and non-smokers. [61-64] In 3 of 4 studies, the HR value of never smokerswas lower than smokers in response to Crizotinib indicating a better response to treatment by non-smokers than non-smokers.

b. Ceritinib

Five publications were found with key words “Ceritinib phase-III” in and three from “Ceritinib phase-3” in the clinical trial category. Two included data for HR fromsmokers and non-smokers [65, 66] in **Tabulation S7**. Results from the two studies werecontradictory indicating the need for more data.

c. Alectinib

Six publications were found with key words“Alectinib phase-III” and two from “Alectinib phase-3” in the clinical trial category, including two comparing HR for smokersand non-smokers. [67, 68] An updated report was published in July 2019 [69] that reported contradictory results.

d. Brigatinib

Two publications were found with key words “Brigatinib phase-III” andone from “Brigatinib phase-3” in the clinical trial category. Onemeasured HR for smokers and non-smokers, reporting that never smokersshowed a lower HRin response to treatment than nonsmokers. [70]

e. Lorlatinib

No publications were found with key words“Lorlatinib phase-III” andonly one from “Lorlatinib phase-3” in the clinical trial category. No data on smokersvs non-smokerswasfound. Although there are few data on individual targeted drugs, no conclusive conclusions can be reached. But as we have summarized thus far, for most drugs used to treat NSCLC, non-smokers responded better than smokers.

Meta-analysis of NSCLC patients treated with drugs targeting ALK gene

We next analyzed separately the HR values from patients treated with drugs targeting the ALK gene (**Tabulation S8**). Together, non-smokers had a mean HR of 0.050 compared to 0.060 for smokers..

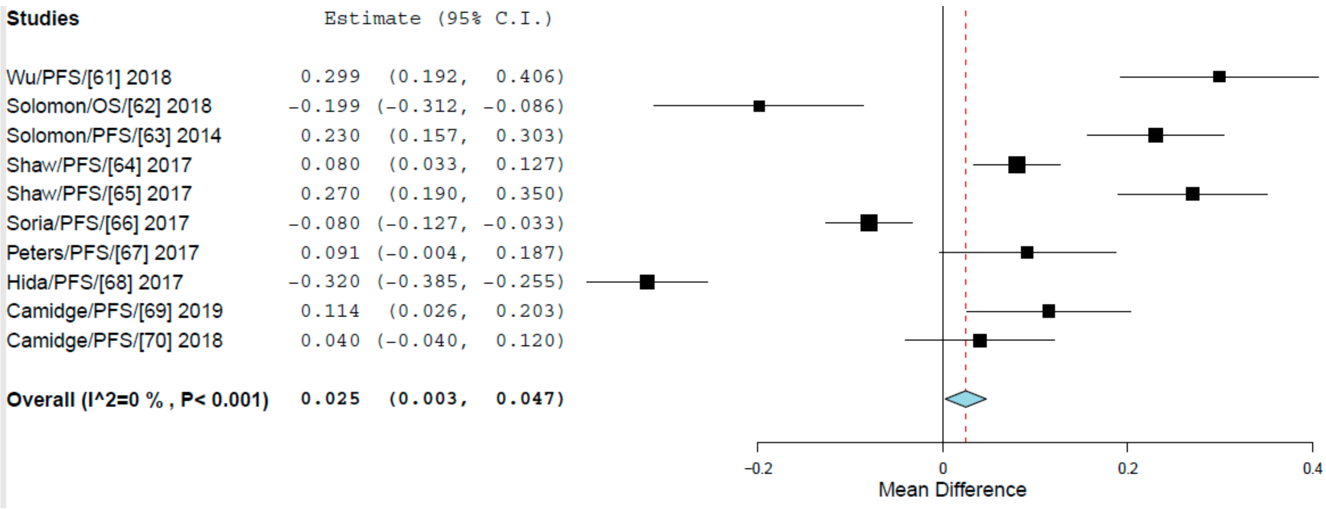

Supplemental Figure s72. Smokers vs non-smokers in response to treatment with drugs targeting ALK with fixed effect model (FE)

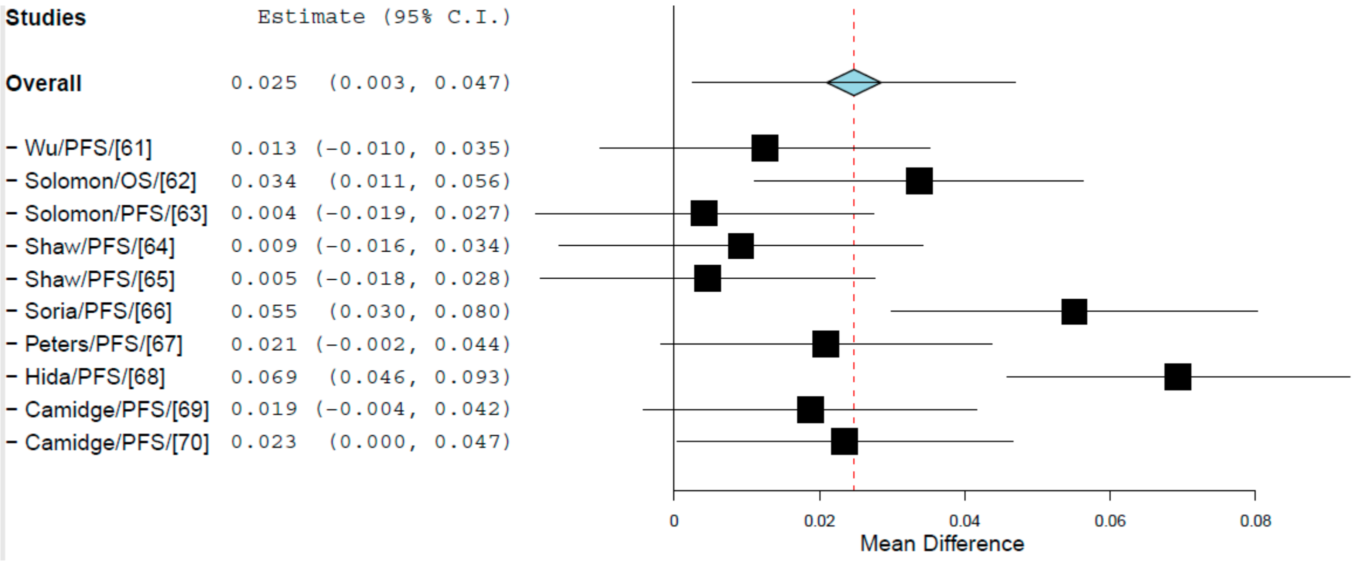

Supplemental Figure s73. Smokers vs non-smokers in response to treatment with drugs targeting ALK with FE leaveoneout model

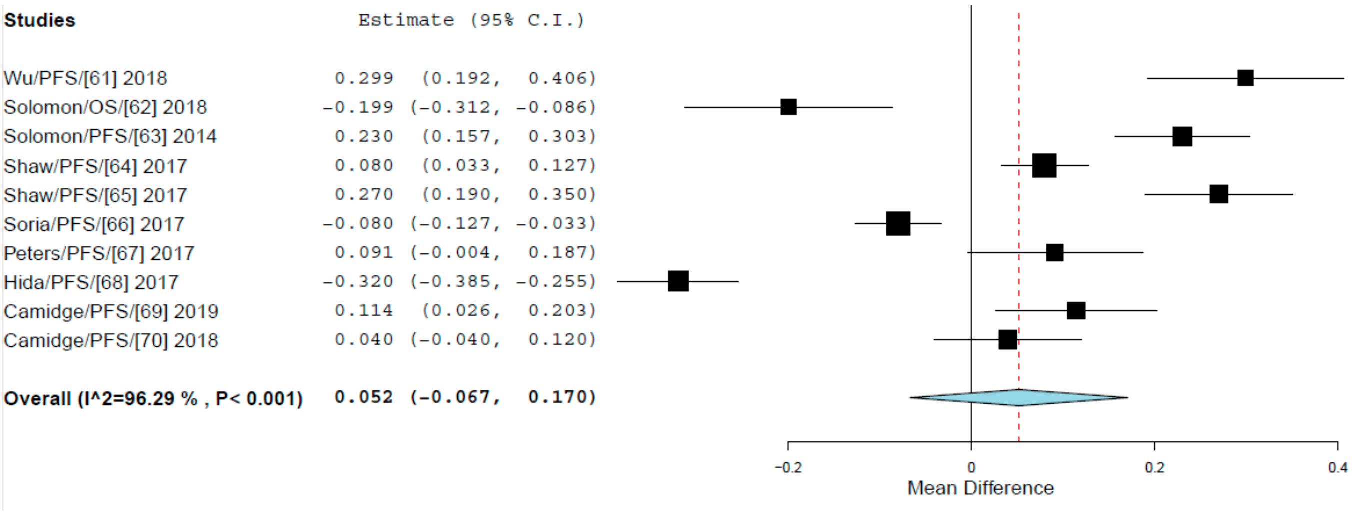

Supplemental Figure s74. Smokers vs non-smokers in response to treatment with drugs targeting ALK with random effect model (RE)

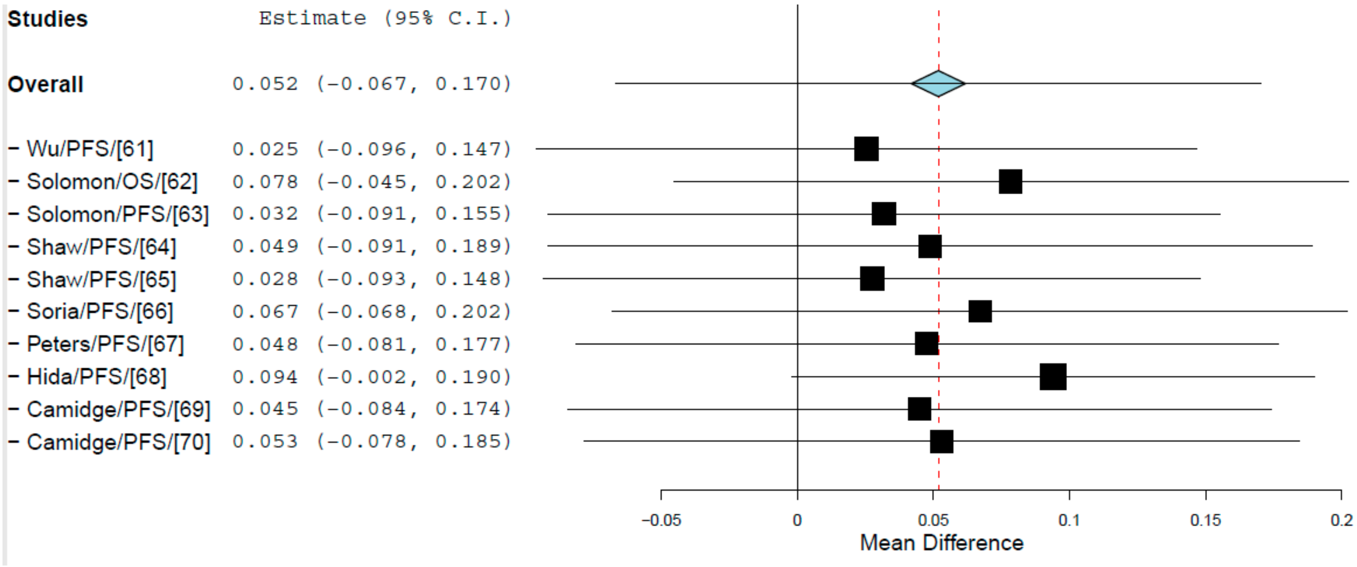

Supplemental Figure s75. Smokers vs non-smokers in response to treatment with drugs targeting ALK with RE leaveoneout model

PART H. Summary of meta-analysis on response to treatment of NSCLC patients to all other drugs

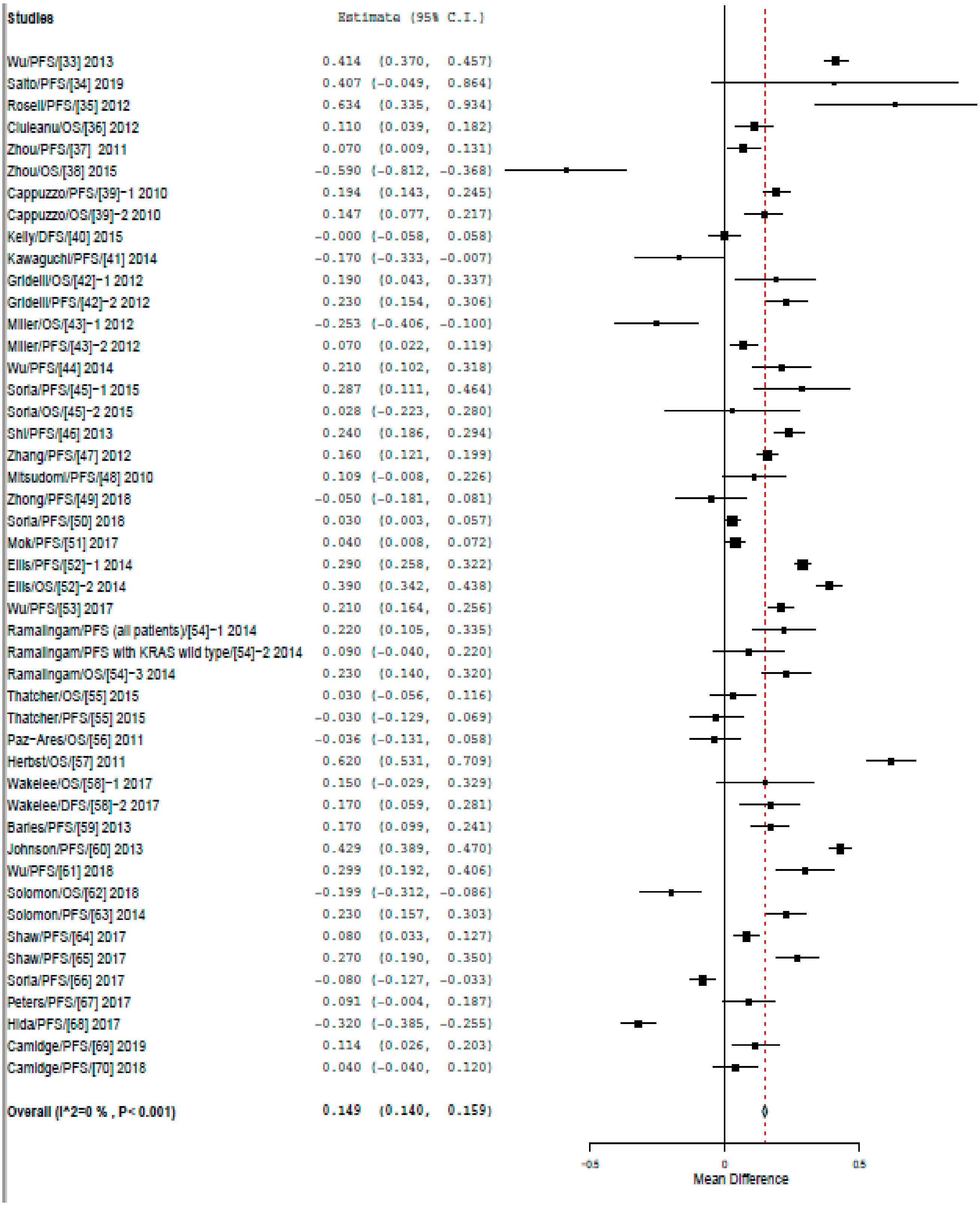

Supplemental Figure s76. Smokers vs non-smokers in response to treatment with all other drugs among NSCLC patients with fixed effect model (FE) of meta-analysis

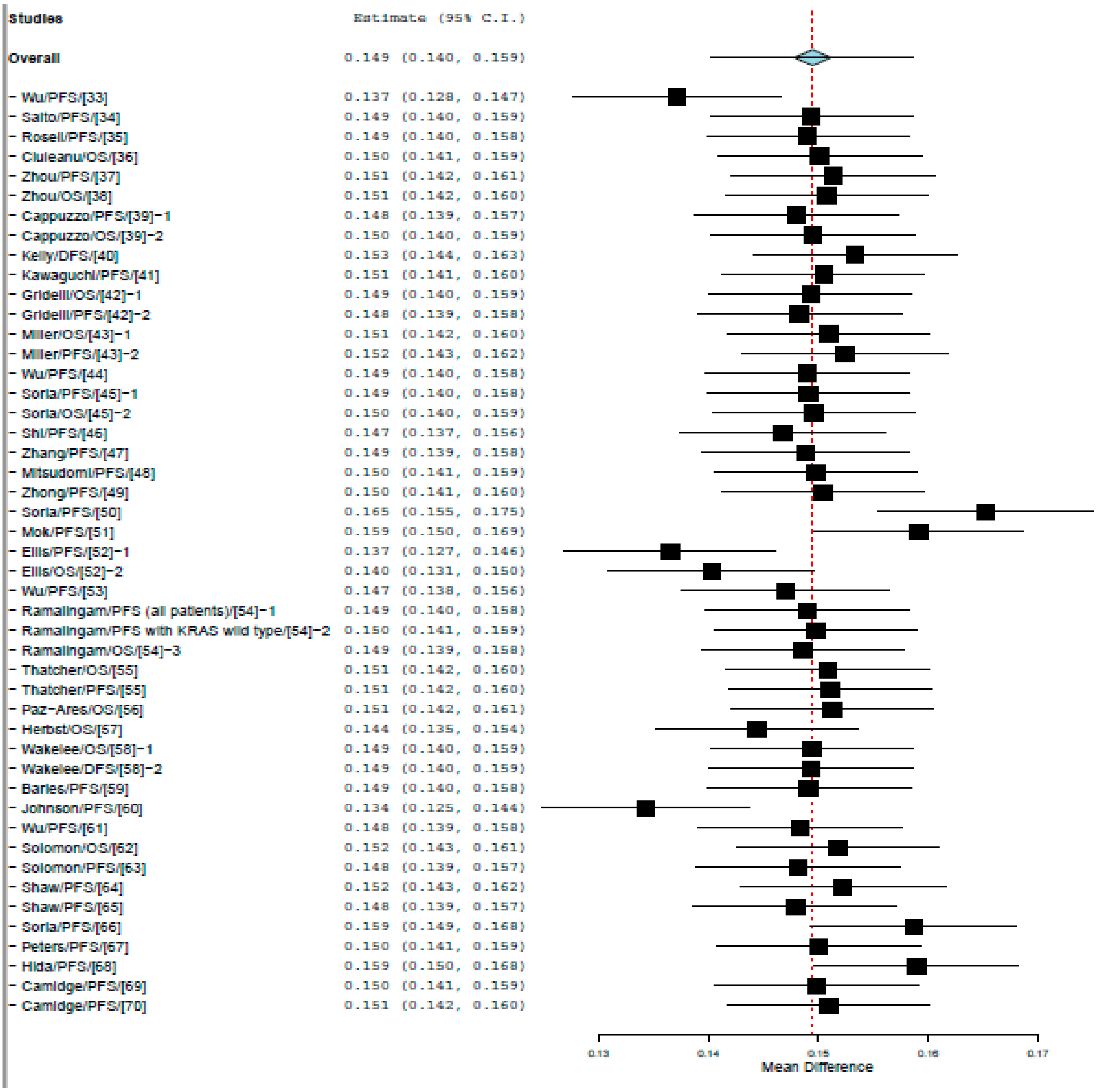

Supplemental Figure s77. Smokers vs non-smokers in response to treatment with all other drugs among NSCLC patients with FE leaveoneout model of meta-analysis

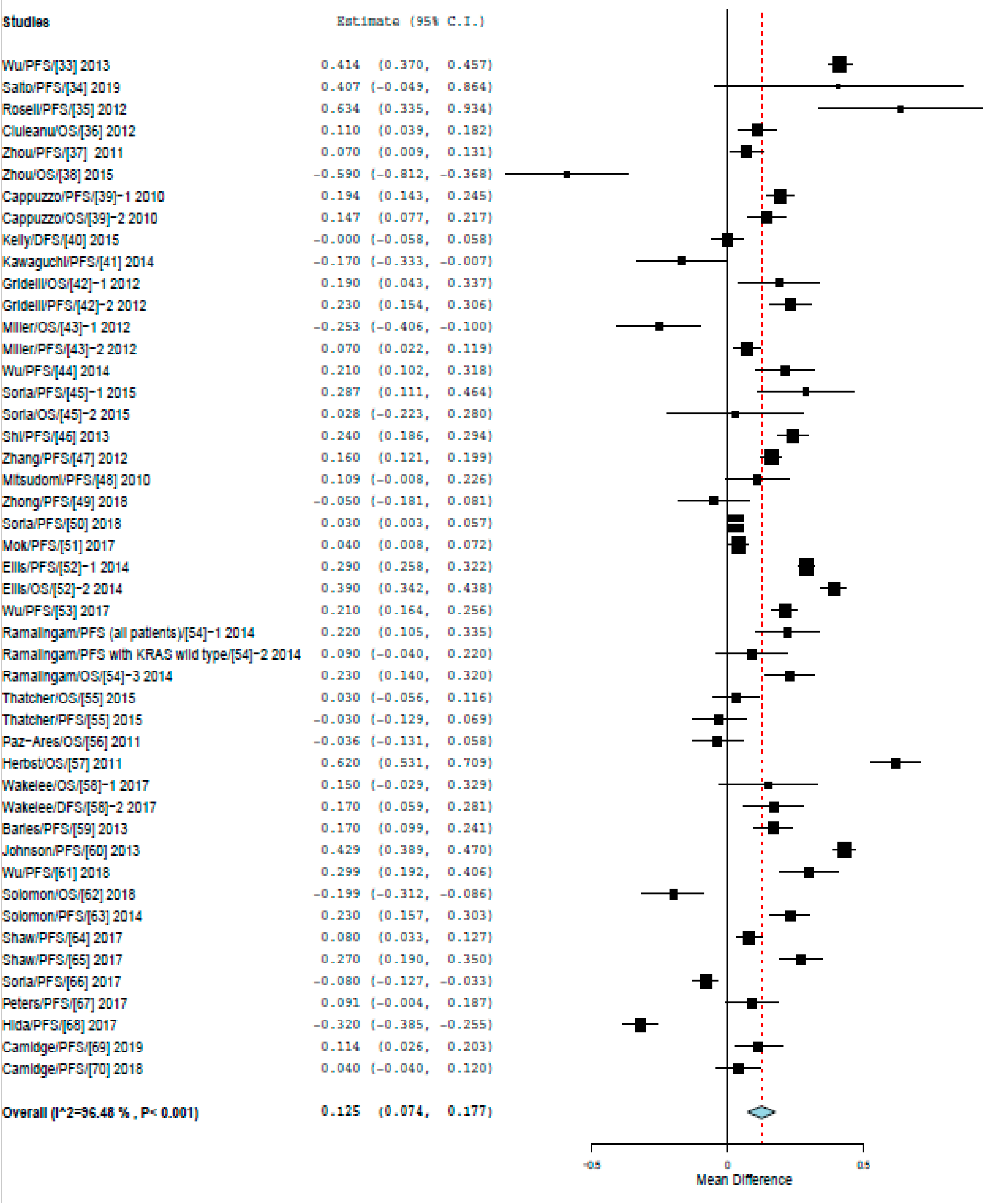

Supplemental Figure s78. Smokers vs non-smokers in response to treatment with all other drugs among NSCLC patients with random modle of meta-analysis

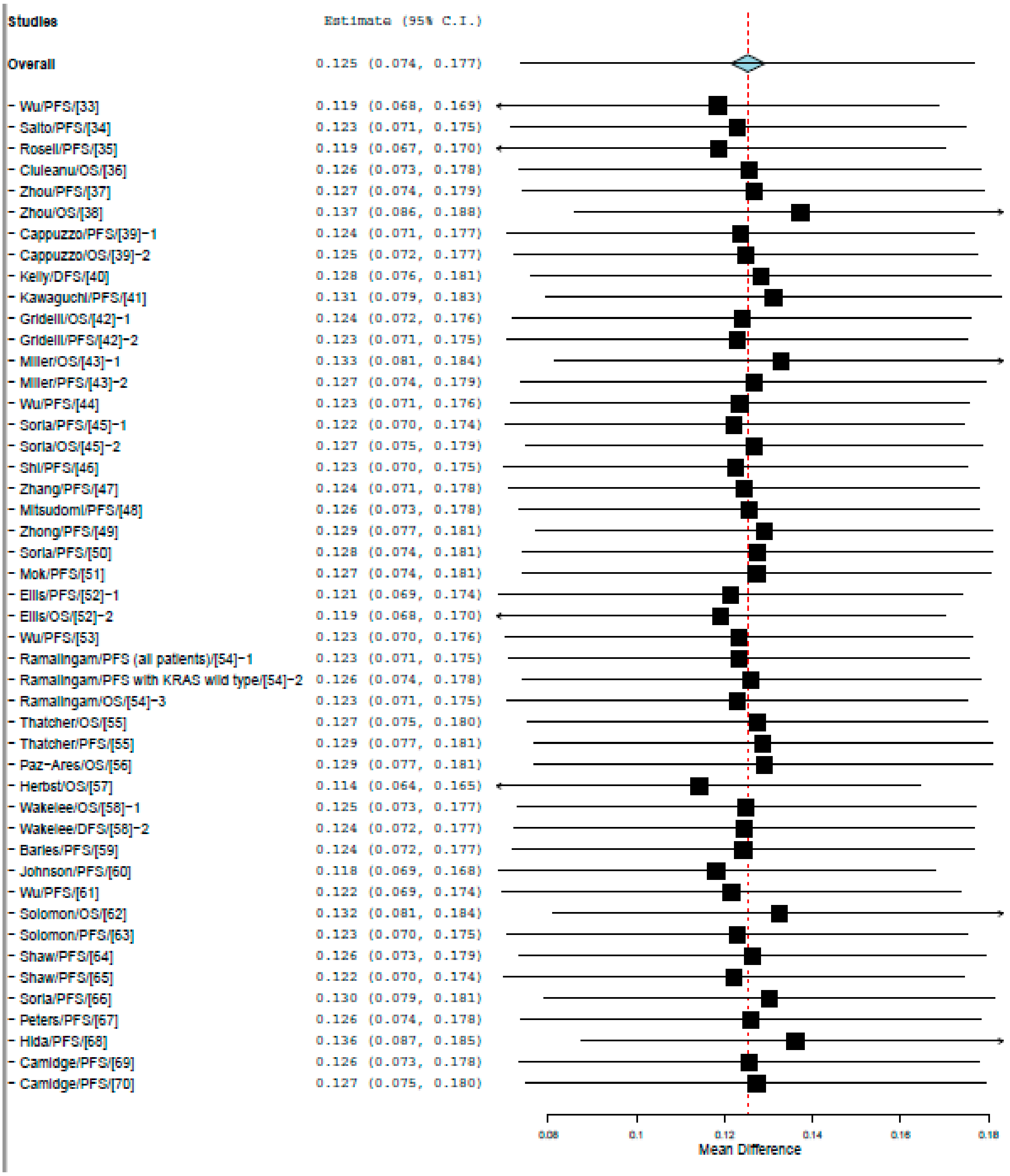

Supplemental Figure s79. Smokers vs non-smokers in response to treatment with all other drugs among NSCLC patients with RE leaveoneout model of meta-analysis

PART I. Response to treatment by smokers and nonsmokers treated with Anti-PD-1 PD-L1 drugs in other cancers

1. Response to treatment by pembrolizumab in other cancer patient populations

A total of 17 publications were obtained by searching with key words “Pembrolizumab phase-3” from the article type of “clinical trial”. [71,72] After excluding NSCLC, ten publications were obtained for clinical trials for six cancers, including renal (2), head-and-neck, gastric or gastro-esophageal, hepatocellular carcinoma, melanoma (3), and urothelial

2. Application of nivolumab in other cancers

A total of 23 publications were obtained by searching with key words “nivolumab phase-3” from the article type of “clinical trial”. Twelve publications were for four other types of cancers, melanoma (5), renal (4), gastric or gastro-oesophageal junction, and head and neck. Smoking status was reported in the study of head and neck cancer. Unlike the data from the pembrolizuma, the HR for OS in non-smokers was lower than that of smokers. [73, 74] These data seem to contradict previous results from other trials, however, the drug was used to treat recurrent or metastatic squamous-cell carcinoma of the head and neck after platinum chemotherapy. Thus, it was not used as the first line of treatment and was limited to a small proportion of PD-L1 positive patients (73 out of 361). In addition, because the investigators analyzed all the smokers together, it is not clear whether the current smokers have a lower HR than non-smokers.

A total of 37 publications were obtained by searching with key words “nivolumab phase-III” from the article type of “clinical trial”. One additional study was found for the treatment of melanoma that did not include smoking status data.

3. Application of atezolizumab in other cancers

A total of seven publications were obtained by searching with key words “atezolizumab phase-3” from the article type of “clinical trial”. Excluding NSCLC, one publication for breast cancer was identified but smoking status was not provided.

A total of eight publications were obtained by searching with key words "atezolizumab phase-III" from the article type of "clinical trial". One publication for urothelial carcinoma was identified reporting smoking status [75]. Again, current smokers had a lower mean HR than non-smokers (**Tabulation S7 in Supplementary**).

4. Application of avelumab in other cancers

A total of four publications were obtained by searching with key words "Avelumab phase-3" from the article type of "clinical trial". Two publications for renal-cell carcinoma were identified but only one reported smoking status. Again, mean HR of smokers was lower than that of non-smokers [76] .

A total of three publications were obtained by searching with key words "Avelumab phase-III" from the article type of "clinical trial". No additional qualified studies were found.

5. Application of durvalumab in other cancers

A total of three publications were obtained by searching with key words "Durvalumab phase-3" from the article type of "clinical trial". No qualifying study was found. Five publications were obtained by searching with key words "Durvalumab phase-III" from the article type of "clinical trial". Again, no qualifying study was found.

The available data from these four trials support the hypothesis that smokers, most likely current smokers, have a lower HR than non-smokers when cancer patients are treated anti PD-1 and PD-L1 drugs. Results for Durvalumab were not apparent. It is not clear when it was used alone or as a first-line of treatment in these studies, or whether smokers respond better than non-smokers. Surprisingly, smokers responded better than non-smokers among patients with other types of cancer when treated with anti-PD-1 Drugs and anti-PD-L1 drugs

Part J. Response to treatment by smokers and nonsmokers to other drugs in patients of other cancers

1.EGFR inhibitors used in other cancers with EGFR gene mutations

a. Erlotinib (Tarceva)

Among140 publications obtained by searching with key words "Erlotinib phase-III" for clinical trials, one study provided the HR values of smokers and non-smokers in the treatment of advanced hepatocellular carcinoma with sorafenib plus erlotinib [77]. Twenty-nine publications were identified when searching with key words "Erlotinib phase-3" (**Tabulation S8 in Supplementary**).

b. Afatinib (Gilotrif)

A total of 140 publications were obtained by searching with key words "Afatinib phase-3", for clinical trials, and 33 publications using Afatinib phase-III' [78] (**Tabulation S8 in Supplementary**).

c. Gefitinib (Iressa)

A total of 20 publications were obtained by searching with key words "Gefitinib phase-3" for clinical trials, and88 publications using key words "Gefitinib phase-III" [79] (**Tabulation S8 in Supplementary**).

d. Osimertinib (Tagrisso)

No data were found.

e. Necitumumab (Portrazza)

No data were identified.

2. Drugs that target tumor blood vessel growth (angiogenesis)

a. Bevacizumab (Avastin)

Among five publications obtained by searching with key words "Bevacizumab phase-III smoking", for clinical trials, only one disease that was not NSCLC (pleural mesothelioma) was reported [80] (**Tabulation S8 in**

Supplementary).

b. Ramucirumab (Cyramza)

Among 37 publications obtained by searching with key words "Ramucirumab phase-III", for clinical trials, none provided any data on smokers vs. non-smokers.

3. Drugs that cells with ALK gene changes

No data on cancers other than NSCLC.

Tabulation S8. HR of smokers and non-smokers of other cancers treated with other Drugs

**Drugs**	**Study (First Author)/drug/analytic Metrix**	**PD-L1 Positive HR**	**PD-L1 negative HR**	**Current/ #Patients**	**Former/ # Patients**	**Never/# Patients**	**Overall/ #Patients**
Erlotinib	Zhu/ OS/sorafenib plus erlotinib/ advanced hepatocellular carcinoma [77]			0.876(0.645-1.191)/241	0.985(0.724-1.334)/260	0.995(0.713-1.387)/219	0.929(0.781-1.106)/720
Afatinib	Machiels /Afatinib versus methotrexate as second-line / Head and Neck/PFS [78]			>=10 pack/year 0.71 (0.56-0.90) /381		<10 pack/year 1.05 (0.66-	0.80(065 - 0.98)/483

						1.70) /87	
Gefitinib	Argiris/OS / of docetaxel with or without gefitinib / head and neck cancer [79]			>40 packs/year/0.87(0.59-1.29)/103	<=40/0.96(0.64-1.44)/131		0.93(0.72-1.21)/239
Bevacizumab	Zalcman/OS/ pleural mesothelioma [80]	-		0.81(0.61-1.08)/254		0.73(0.53-1.02)/194	0.77(0.62-0.95)/348

Supplemental Tabulation s9. Reference in the supplemental materials

Ref. No.	First Author	References
2	Barlesi	Barlesi F, Vansteenkiste J, Spigel D, et al. Avelumab versus docetaxel in patients with platinum-treated advanced non-small-cell lung cancer (JAVELIN Lung 200): an open-label, randomised, phase 3 study. Lancet Oncol. 2018;19(11):1468-1479.
4	Mok	Mok TSK, Wu YL, Kudaba I, et al. Pembrolizumab versus chemotherapy for previously untreated, PD-L1-expressing, locally advanced or metastatic non-small-cell lung cancer (KEYNOTE-042): a randomised, open-label, controlled, phase 3 trial. Lancet. 2019;393(10183):1819-1830.
20	Reck	Reck M, Rodriguez-Abreu D, Robinson AG, et al. Pembrolizumab versus Chemotherapy for PD-L1-Positive Non-Small-Cell Lung Cancer. N Engl J Med. 2016;375(19):1823-1833.
21	Gandhi	Gandhi L, Rodriguez-Abreu D, Gadgeel S, et al. Pembrolizumab plus Chemotherapy in Metastatic Non-Small-Cell Lung Cancer. N Engl J Med. 2018;378(22):2078-2092.
24	Borghaei	Borghaei H, Paz-Ares L, Horn L, et al. Nivolumab versus Docetaxel in Advanced Nonsquamous Non-Small-Cell Lung Cancer. N Engl J Med. 2015;373(17):1627-1639.
28	Socinski	Socinski MA, Jotte RM, Cappuzzo F, et al. Atezolizumab for First-Line Treatment of Metastatic Nonsquamous NSCLC. N Engl J Med. 2018;378(24):2288-2301.

29	Rittmeyer	Rittmeyer A, Barlesi F, Waterkamp D, et al. Atezolizumab versus docetaxel in patients with previously treated non-small-cell lung cancer (OAK): a phase 3, open-label, multicentre randomised controlled trial. Lancet. 2017;389(10066):255-265.
30	West	West H, McCleod M, Hussein M, et al. Atezolizumab in combination with carboplatin plus nab-paclitaxel chemotherapy compared with chemotherapy alone as first-line treatment for metastatic non-squamous non-small-cell lung cancer (IMpower130): a multicentre, randomised, open-label, phase 3 trial. Lancet Oncol. 2019;20(7):924-937.
31	Antonia	Antonia SJ, Villegas A, Daniel D, et al. Durvalumab after Chemoradiotherapy in Stage III Non-Small-Cell Lung Cancer. N Engl J Med. 2017;377(20):1919-1929.
32	Antonia	Antonia SJ, Villegas A, Daniel D, et al. Overall Survival with Durvalumab after Chemoradiotherapy in Stage III NSCLC. N Engl J Med. 2018;379(24):2342-2350.
33	Wu	Wu YL, Lee JS, Thongprasert S, et al. Intercalated combination of chemotherapy and erlotinib for patients with advanced stage non-small-cell lung cancer (FASTACT-2): a randomised, double-blind trial. Lancet Oncol. 2013;14(8):777-786.
34	Saito	Saito H, Fukuhara T, Furuya N, et al. erlotinib plus bevacizumab versus erlotinib alone in patients with EGFR-positive advanced non-squamous non-small-cell lung cancer (NEJ026): interim analysis of an open-label, randomised, multicentre, phase 3 trial. Lancet Oncol. 2019;20(5):625-635.
35	Rosell	Rosell R, Carcereny E, Gervais R, et al. erlotinib versus standard chemotherapy as first-line treatment for European patients with advanced EGFR mutation-positive non-small-cell lung cancer (EURTAC): a multicentre, open-label, randomised phase 3 trial. The Lancet Oncology. 2012;13(3):239-246.
36	Ciuleanu	Ciuleanu T, Stelmakh L, Cicenas S, et al. Efficacy and safety of erlotinib versus chemotherapy in second-line treatment of patients with advanced, non-small-cell lung cancer with poor prognosis (TITAN): a randomised multicentre, open-label, phase 3 study. Lancet Oncol. 2012;13(3):300-308.
37	Zhou	Zhou C, Wu Y-L, Chen G, et al. erlotinib versus chemotherapy as first-line treatment for patients with advanced EGFR mutation-positive non-small-cell lung cancer (OPTIMAL, CTONG-0802): a multicentre, open-label, randomised, phase 3 study. The Lancet Oncology. 2011;12(8):735-742.
38	Zhou	Zhou C, Wu Y, Chen G, et al. Final overall survival results from a randomised, phase III study of erlotinib versus chemotherapy as first-line treatment of EGFR mutation-positive advanced non-small-cell lung cancer (OPTIMAL, CTONG-0802). Annals of Oncology. 2015;26(9):1877-1883.
39	Cappuzzo	Cappuzzo F, Ciuleanu T, Stelmakh L, et al. erlotinib as maintenance treatment in advanced non-small-cell lung cancer: a multicentre, randomised, placebo-controlled phase 3 study. The Lancet Oncology. 2010;11(6):521-529.
40	Kelly	Kelly K, Altorki NK, Eberhardt WE, et al. Adjuvant erlotinib Versus Placebo in Patients With Stage IB-IIIA Non-Small-Cell Lung Cancer (RADIANT): A Randomized, Double-Blind, Phase III Trial. J Clin Oncol. 2015;33(34):4007-4014.
41	Kawaguchi	Kawaguchi T, Ando M, Asami K, et al. Randomized phase III trial of erlotinib versus docetaxel as second- or third-line therapy in patients with advanced non-small-cell lung cancer: Docetaxel and erlotinib Lung Cancer Trial (DELTA). J Clin Oncol. 2014;32(18):1902-1908.
42	Gridelli	Gridelli C, Ciardiello F, Gallo C, et al. First-line erlotinib followed by second-line cisplatin-gemcitabine chemotherapy in advanced non-small-cell lung cancer: the TORCH randomized trial. J Clin Oncol. 2012;30(24):3002-3011.
43	Miller	Miller VA, Hirsh V, Cadranel J, et al. Afatinib versus placebo for patients with advanced, metastatic non-small-cell lung cancer after failure of erlotinib, Gefitinib, or both, and one or two lines of chemotherapy (LUX-Lung 1): a phase 2b/3 randomised trial. Lancet Oncol. 2012;13(5):528-538.

44	Wu	Wu YL, Zhou C, Hu CP, et al. Afatinib versus cisplatin plus gemcitabine for first-line treatment of Asian patients with advanced non-small-cell lung cancer harbouring EGFR mutations (LUX-Lung 6): an open-label, randomised phase 3 trial. Lancet Oncol. 2014;15(2):213-222.
45	Soria	Soria J-C, Felip E, Cobo M, et al. Afatinib versus erlotinib as second-line treatment of patients with advanced squamous cell carcinoma of the lung (LUX-Lung 8): an open-label randomised controlled phase 3 trial. The Lancet Oncology. 2015;16(8):897-907.
46	Shi	Shi Y, Zhang L, Liu X, et al. Icotinib versus Gefitinib in previously treated advanced non-small-cell lung cancer (ICOGEN): a randomised, double-blind phase 3 non-inferiority trial. Lancet Oncol. 2013;14(10):953-961.
47	Zhang	Zhang L, Ma S, Song X, et al. Gefitinib versus placebo as maintenance therapy in patients with locally advanced or metastatic non-small-cell lung cancer (INFORM; C-TONG 0804): a multicentre, double-blind randomised phase 3 trial. The Lancet Oncology. 2012;13(5):466-475.
48	Mitsudomi	Mitsudomi T, Morita S, Yatabe Y, et al. Gefitinib versus cisplatin plus docetaxel in patients with non-small-cell lung cancer harbouring mutations of the epidermal growth factor receptor (WJTOG3405): an open label, randomised phase 3 trial. Lancet Oncol. 2010;11(2):121-128.
49	Zhong	Zhong W-Z, Wang Q, Mao W-M, et al. Gefitinib versus vinorelbine plus cisplatin as adjuvant treatment for stage II–IIIA (N1–N2) EGFR-mutant NSCLC (ADJUVANT/CTONG1104): a randomised, open-label, phase 3 study. The Lancet Oncology. 2018;19(1):139-148.
50	Soria	Soria JC, Ohe Y, Vansteenkiste J, et al. Osimertinib in Untreated EGFR-Mutated Advanced Non-Small-Cell Lung Cancer. N Engl J Med. 2018;378(2):113-125.
51	Mok	Mok TS, Wu YL, Ahn MJ, et al. Osimertinib or Platinum-Pemetrexed in EGFR T790M-Positive Lung Cancer. N Engl J Med. 2017;376(7):629-640.
52	Ellis	Ellis PM, Shepherd FA, Millward M, et al. Dacomitinib compared with placebo in pretreated patients with advanced or metastatic non-small-cell lung cancer (NCIC CTG BR.26): a double-blind, randomised, phase 3 trial. Lancet Oncol. 2014;15(12):1379-1388.
53	Wu	Wu Y-L, Cheng Y, Zhou X, et al. Dacomitinib versus Gefitinib as first-line treatment for patients with EGFR-mutation-positive non-small-cell lung cancer (ARCHER 1050): a randomised, open-label, phase 3 trial. The Lancet Oncology. 2017;18(11):1454-1466.
54	Ramalingam	Ramalingam SS, Janne PA, Mok T, et al. Dacomitinib versus erlotinib in patients with advanced-stage, previously treated non-small-cell lung cancer (ARCHER 1009): a randomised, double-blind, phase 3 trial. Lancet Oncol. 2014;15(12):1369-1378.
55	Thather	Thatcher N, Hirsch FR, Luft AV, et al. Necitumumab plus gemcitabine and cisplatin versus gemcitabine and cisplatin alone as first-line therapy in patients with stage IV squamous non-small-cell lung cancer (SQUIRE): an open-label, randomised, controlled phase 3 trial. Lancet Oncol. 2015;16(7):763-774.
56	Paz-Ares	Paz-Ares L, Mezger J, Ciuleanu TE, et al. Necitumumab plus pemetrexed and cisplatin as first-line therapy in patients with stage IV non-squamous non-small-cell lung cancer (INSPIRE): an open-label, randomised, controlled phase 3 study. The Lancet Oncology. 2015;16(3):328-337.
57	Herbst	Herbst RS, Ansari R, Bustin F, et al. Efficacy of bevacizumab plus erlotinib versus erlotinib alone in advanced non-small-cell lung cancer after failure of standard first-line chemotherapy (BeTa): a double-blind, placebo-controlled, phase 3 trial. Lancet. 2011;377(9780):1846-1854.

58	Wakelee	Wakelee HA, Dahlberg SE, Keller SM, et al. Adjuvant chemotherapy with or without bevacizumab in patients with resected non-small-cell lung cancer (E1505): an open-label, multicentre, randomised, phase 3 trial. The Lancet Oncology. 2017;18(12):1610-1623.
59	Barlesi	Barlesi F, Scherpereel A, Rittmeyer A, et al. Randomized phase III trial of maintenance bevacizumab with or without pemetrexed after first-line induction with bevacizumab, cisplatin, and pemetrexed in advanced nonsquamous non-small-cell lung cancer: AVAPERL (MO22089). J Clin Oncol. 2013;31(24):3004-3011.
60	Johnson	Johnson BE, Kabbinavar F, Fehrenbacher L, et al. ATLAS: randomized, double-blind, placebo-controlled, phase IIIB trial comparing bevacizumab therapy with or without erlotinib, after completion of chemotherapy, with bevacizumab for first-line treatment of advanced non-small-cell lung cancer. J Clin Oncol. 2013;31(31):3926-3934.
61	Wu	Wu YL, Lu S, Lu Y, et al. Results of PROFILE 1029, a Phase III Comparison of First-Line Crizotinib versus Chemotherapy in East Asian Patients with ALK-Positive Advanced Non-Small Cell Lung Cancer. J Thorac Oncol. 2018;13(10):1539-1548.
62	Solomon	Solomon BJ, Kim D-W, Wu Y-L, et al. Final overall survival analysis from a study comparing first-line crizotinib versus chemotherapy in ALK-mutation-positive non–small-cell lung cancer. Journal of Clinical Oncology. 2018;36(22):2251-2258.
63	Solomon	Solomon BJ, Mok T, Kim DW, et al. First-line crizotinib versus chemotherapy in ALK-positive lung cancer. N Engl J Med. 2014;371(23):2167-2177.
64	Shaw	Shaw/ versus chemotherapy/ ALK-positive<br>Crizotinib
65	Shaw	Shaw AT, Kim TM, Crino L, et al. Ceritinib versus chemotherapy in patients with ALK-rearranged non-small-cell lung cancer previously given chemotherapy and crizotinib (ASCEND-5): a randomised, controlled, open-label, phase 3 trial. Lancet Oncol. 2017;18(7):874-886.
66	Soria	Soria JC, Tan DSW, Chiari R, et al. First-line ceritinib versus platinum-based chemotherapy in advanced ALK-rearranged non-small-cell lung cancer (ASCEND-4): a randomised, open-label, phase 3 study. Lancet. 2017;389(10072):917-929.
67	Peters	Peters S, Camidge DR, Shaw AT, et al. Alectinib versus Crizotinib in Untreated ALK-Positive Non-Small-Cell Lung Cancer. N Engl J Med. 2017;377(9):829-838.
68	Hida	Hida T, Nokihara H, Kondo M, et al. Alectinib versus crizotinib in patients with ALK-positive non-small-cell lung cancer (J-ALEX): an open-label, randomised phase 3 trial. Lancet. 2017;390(10089):29-39.
69	Camidge	Camidge DR, Dziadziuszko R, Peters S, et al. Updated Efficacy and Safety Data and Impact of the EML4-ALK Fusion Variant on the Efficacy of Alectinib in Untreated ALK-Positive Advanced Non-Small Cell Lung Cancer in the Global Phase III ALEX Study. J Thorac Oncol. 2019;14(7):1233-1243.
70	Camidge	Camidge DR, Kim HR, Ahn MJ, et al. Brigatinib versus Crizotinib in ALK-Positive Non-Small-Cell Lung Cancer. N Engl J Med. 2018;379(21):2027-2039.
71	Bellmunt	Bellmunt J, De Wit R, Vaughn DJ, et al. Pembrolizumab as second-line therapy for advanced urothelial carcinoma. New England Journal of Medicine. 2017;376(11):1015-1026.
72	Cohen	Cohen EEW, Soulieres D, Le Tourneau C, et al. Pembrolizumab versus methotrexate, docetaxel, or cetuximab for recurrent or metastatic head-and-neck squamous cell carcinoma (KEYNOTE-040): a randomised, open-label, phase 3 study. Lancet. 2019;393(10167):156-167.
73	Ferris	Ferris RL, Blumenschein G, Jr., Fayette J, et al. Nivolumab for Recurrent Squamous-Cell Carcinoma of the Head and Neck. N Engl J Med. 2016;375(19):1856-1867.

74	Escudier	Escudier B, Sharma P, McDermott DF, et al. CheckMate 025 Randomized Phase 3 Study: Outcomes by Key Baseline Factors and Prior Therapy for Nivolumab Versus Everolimus in Advanced Renal Cell Carcinoma. Eur Urol. 2017;72(6):962-971.
75	Powles	Powles T, Duran I, van der Heijden MS, et al. Atezolizumab versus chemotherapy in patients with platinum-treated locally advanced or metastatic urothelial carcinoma (IMvigor211): a multicentre, open-label, phase 3 randomised controlled trial. Lancet. 2018;391(10122):748-757.
76	Motzer	Motzer RJ, Penkov K, Haanen J, et al. Avelumab plus Axitinib versus Sunitinib for Advanced Renal-Cell Carcinoma. N Engl J Med. 2019;380(12):1103-1115.
77	Zhu	Zhu AX, Rosmorduc O, Evans T, et al. SEARCH: a phase III, randomized, double-blind, placebo-controlled trial of sorafenib plus erlotinib in patients with advanced hepatocellular carcinoma. Journal of Clinical Oncology, 2014, vol 33, num 6, p 559-566. 2014.
78	Machiels	Machiels J-PH, Haddad RI, Fayette J, et al. Afatinib versus methotrexate as second-line treatment in patients with recurrent or metastatic squamous-cell carcinoma of the head and neck progressing on or after platinum-based therapy (LUX-Head & Neck 1): an open-label, randomised phase 3 trial. The Lancet Oncology. 2015;16(5):583-594.
79	Argiris	Argiris A, Ghebremichael M, Gilbert J, et al. Phase III randomized, placebo-controlled trial of docetaxel with or without Gefitinib in recurrent or metastatic head and neck cancer: an eastern cooperative oncology group trial. Journal of clinical oncology. 2013;31(11):1405.
80	Zalcman	Zalcman G, Mazieres J, Margery J, et al. Bevacizumab for newly diagnosed pleural mesothelioma in the Mesothelioma Avastin Cisplatin Pemetrexed Study (MAPS): a randomised, controlled, open-label, phase 3 trial. Lancet. 2016;387(10026):1405-1414.
